# The Qigong of Prolong Life With Nine Turn Method Relieve Fatigue, Sleep, Anxiety and Depression in Patients With Chronic Fatigue Syndrome: A Randomized Controlled Clinical Study

**DOI:** 10.3389/fmed.2022.828414

**Published:** 2022-06-30

**Authors:** Fangfang Xie, Yanli You, Chong Guan, Jiatuo Xu, Fei Yao

**Affiliations:** ^1^Department of Acupuncture and Massage Rehabilitation Center, Shanghai Municipal Hospital of Traditional Chinese Medicine, Shanghai University of Traditional Chinese Medicine, Shanghai, China; ^2^Department of School of Basic Medicine, Shanghai University of Traditional Chinese Medicine, Shanghai, China; ^3^Department of Traditional Chinese Medicine, ChangHai Hospital, Naval Medical University, Shanghai, China

**Keywords:** qigong, prolong life with nine turn method, chronic fatigue syndrome, fatigue, sleep quality, depression and anxiety

## Abstract

**Background:**

Chronic fatigue syndrome (CFS) is a complex disease of unknown etiology and mechanism. The purpose of this study was to investigate the effect of Prolong Life with Nine Turn Method (PLWNT) Qigong exercise on CFS focusing on fatigue, sleep quality, depression, and anxiety.

**Methods:**

A total of 90 participants diagnosed with CFS were randomly assigned into two parallel groups: PLWNT and cognitive behavioral therapy (CBT). The participants in the PLWNT or CBT group participated in qigong exercise or cognitive behavior education program, respectively, once a week in-person and were supervised online during the remaining 6 days at home, over 12 consecutive weeks. The primary outcome was fatigue (Multi-dimensional Fatigue Inventory 20 [MFI-20]), and secondary outcomes were sleep quality (Pittsburgh Sleep Quality Index [PSQI]), anxiety, depression (Hospital Anxiety and Depression Scale [HADS]), and changes in the Neuropeptide Y (NPY) of peripheral blood.

**Results:**

The within-group comparisons of the PLWNT and CBT groups revealed significant improvement in both groups in MFI-20, PSQI, and HADS scores (*P* < 0.05). No significant difference were found between the PLWNT and CBT groups, even though the effective rate of the PLWNT group was 62.22%, which is slightly than 50.00% of the CBT group. The fatigue scores in the PLWNT group were positively correlated with sleep degree (*r* = 0.315) and anxiety degree (*r* = 0.333), only anxiety degree (*r* = 0.332) was found to be positively correlated with fatigue in the CBT group. The analysis of peripheral blood showed that NPY decreased after PLWNT intervention but increased significantly in the CBT.

**Conclusion:**

The PLWNT qigong exercise has potential to be an effective rehabilitation method for CFS symptoms including fatigue, sleep disturbance, anxiety, and depression. Future studies should expand study sample size for in-depth investigation to determine the optimal frequency and intensity of PLWNT qigong intervention in CFS patients. The study was registered in the ClinicalTrials.gov database on April 12, 2018, with registration number NCT03496961.

## Introduction

Chronic fatigue syndrome, also called myalgic encephalomyelitis, is a complex multisystem disease commonly characterized by severe fatigue, cognitive dysfunction, sleep problems, autonomic dysfunction, and post-exertional malaise severely impairing activities of daily living (1). According to global statistics, approximately 1% of the worldwide population (17–24 million people) suffer from CFS, along with significant incidence rates of comorbidities including psychiatric disorder and cognitive symptoms (2). Patients with CFS are forced to reduce 50% of their daily activities (3), and 87% to 95% of them have related daytime dysfunction (4, 5). A large sample of 1,409 CFS patients showed that 95% of people with higher education and full-time work had sleep disorders, while 68 to 80% suffered from anxiety and depression symptoms (6), further exacerbating the decline in quality of life (7, 8). In addition, fatigue can occur independently of other symptoms of CFS, but it is usually related to sleep and mood disorders (9, 10). A study reported that fatigue has a negative impact on all areas of quality of life, including physical and emotional health, activity ability, and activities of daytime living (11). The increasing incidence of CFS means that it not only threatens the personal wellbeing of more people but also brings a heavy burden to families and society.

Since the etiology and pathology of CFS are unknown, symptomatic treatments are used clinically, including drugs, graded exercise therapy (GET), and cognitive–behavioral therapy (CBT), to attempt to relieve CFS symptoms and improve quality of life. The CFS treatment guidelines emphasize a change in the overall treatment attitude of CFS—for instance, drug therapy as the main rule for the management of patients with CFS should be revised and non-pharmaceutical therapies should been recommended in CFS patients (12). Both the GET and CBT interventions were built on a behavior/disadaptation model of CFS. The GET was designed to help CFS patients overcome this purported fear of exercise and intense symptom-focusing through graded exposure to exercise, and thereby reversing any deconditioning that had occurred (13). The CBT had similar aims, but addressed the fear of activity, maladaptive disease beliefs and symptom focusing by combining CBT and practical activities (14). Several researchers have proposed that graded exercise therapy and CBT might be effective treatments for CFS to improve fatigue and poor mental health, including depression, anxiety, and schizophrenia (13, 15). However, evidence of persistent and sustained significant outcomes in CFS patients is not sufficient (16). The clinical manual published by the International Association for Chronic Fatigue Syndrome recommends traditional Chinese medicine (TCM) treatments as a complementary alternative therapy, including acupuncture and massage (17).

Qigong (pronounced “chee-gun”) is one of the TCM methods that has been used for thousands of years to optimize and restore the energy of the body, mind, and spirit (18). It is a regular, moderate-intensity aerobic exercise (19). Specifically, the term “qigong” involves two theories: “qi,” the righteous qi of the body, which represents the essence flowing in the human body, it is manifested in the constant movement of body energy and the constant alternation of inhalation and exhalation, supporting all life processes and connecting all vital organs of the body (20). “Gong,” the training or cultivation of qi. Qigong, such as Tai Chi, is a mind-body techniques, specific postures and movements based on breathing exercises to achieve a state of deep focus and relaxation, all of which aim to cultivate righteousness and achieve functional enhancement, thereby improving related symptoms (21). Acupuncture, Tuina and Qigong can all regulate Qi, and it has been clinically proven to be effective in the treatment of CFS (6, 22, 23).

Prolong Life with Nine Turn Method (PLWNT) is a type of qigong practice that uses external energy to strengthen the limbs and internal energy to reconcile the viscera. This practice sims to smooth the circulating qi and blood that was introduced by a centenarian named Kai Fang in the Qing Dynasty. It has been written into the college textbook of Tuina and Qigong, which includes eight kinds of massage manipulations of the abdomen and a kind of upper body shaking (24). The abdominal massage techniques included in PLWNT act on the movement of the pelvic and abdominal muscles, coordinated with diaphragmatic breathing. It may trigger the contraction of the intestinal and rectal muscles (25), which can train the function of the intestines (26), but also have an impact on the nervous system, including reducing the excitability of the sympathetic nerve and enhancing the excitability of the parasympathetic nerve to reduce anxiety when rubbing the internal organs (27, 28). Specifically, abdominal massage manipulation included in PLWNT therapy may relieve muscle tension and nerve rhythm to relieve sleep disorders, fatigue, and depression symptoms of fibromyalgia syndrome (FMS) similar to CFS patients (29), and fatigue is a coexisting symptom of FMS and CFS, with up to 80% of CFS patients reported a history of clinician-diagnosed FMS (30, 31). Previous studies have proved the efficacy of PLWNT in the treatment of patients with gastrointestinal diseases (32, 33), but it is not clear whether PLWNT has an effect on fatigue, sleep and mood. Our published protocol for this project has predicted that PLWNT qigong exercise can improve fatigue, sleep disorders, and depression in CFS patients (34), therefore, this study compares the effects of PLWNT and CBT therapies to verify the effective methods to improve fatigue, sleep, anxiety and depression in CFS patients.

The current study was to evaluate the effects of PLWNT. We hypothesized that PLWNT would help mitigate related fatigue, sleep, and depression symptoms of CFS (primary outcomes) and better than CBT. We present the following article/case in accordance with the Consolidated Standards of Reporting Trials reporting checklist.

## Materials and Methods

### Study Design

The present study was a randomized controlled trial involving the following two parallel groups: PLWNT and CBT. All participants were recruited from December 2018 to September 2019 at the Shanghai University of Traditional Chinese Medicine and Yueyang Hospital of Integrated Traditional Chinese and Western Medicine in Shanghai, China. A statistician who did not participate in the recruitment randomly placed eligible CFS patients into the two groups using sealed envelope randomization using a computer software program (Strategic Applications Software, version 9.1.3; SAS Institute Inc., Cary, NC, USA) to create a random number table, then compiled a set of sealed envelopes on the basis of the random sequence and put the patient's information, treatment method, time, and location in an opaque envelope according to the random numbers. Finally, they handed the envelopes over to the research team. The study was conducted in accordance with the Declaration of Helsinki and the International Code of Ethics for Biomedical Research Involving Human Subjects, was approved by the ethics committee of Yueyang Hospital of Integrated Traditional Chinese and Western Medicine (ethics approval no. 2018-043), and was registered in the ClinicalTrials.gov database run by the United States National Library of Medicine on April 12, 2018, under registration number NCT03496961.

### Sample Size Calculation

According to our recently published protocol (34), the efficacy of the PLWNT group was assumed to be better than that of the CBT group. With reference to studies on the efficacy of CFS on the FSS scale (35, 36), it was calculated that the final difference between the two groups in terms of FSS average scores is 0.915 and the standard deviation is 1.147. The Bonferroni conservative comparison method was used, and the sample size of this trial was calculated using the following formula (37):


(1) $$\begin{array}{l} n=\frac{2\times {\left(Z_{\alpha /4}+{\text{Z}}_{\beta }\right)}^{2}\times \sigma^{2}}{\delta^{2}} \end{array}$$

(2) $$\begin{array}{l} =\frac{2\times {\left(2.2414\;+\;1.282\right)}^{2}\times 1.1479^{2}}{0.915^{2}}=39.08\approx 40 \end{array}$$

Considering the allowable 10% dropout rate, the sample size of each group in this experiment was set at 45. Therefore, this randomized controlled trial needed to recruit 90 participants in total.

### Subjects

A total of 90 participants were recruited via WeChat (Tencent co., Ltd., China) or posters positioned at the Shanghai University of Traditional Chinese Medicine and Yueyang Hospital of Integrated Traditional Chinese and Western Medicine in Shanghai, China. Hospitalized patients were also included with a preliminary diagnosis of CFS, according to the latest guidelines for the treatment of CFS revised in 2021 (38).

The study inclusion criteria were as follows: (1) age between 20 and 60 years; male or female; (2) severe chronic fatigue lasting at least 6 months, unexplained after clinical evaluation, not caused by work performed during the trial, and unable to be alleviated after rest; and (3) at least four of eight specific symptoms (memory or concentration decline, failure to regain energy after sleep, sore throat, headache, lymph node tenderness, muscle pain, multiple joint pain, and myalgia after exertion for more than 24 h). Separately, the study exclusion criteria were as follows: severe cardiovascular and cerebrovascular diseases, endocrine system diseases, motor system diseases, autoimmune diseases, infectious diseases, and use of medications that may affect the judgment of the results.

Patients who met the inclusion and exclusion criteria underwent baseline measurements (T_0_) and were randomly assigned to the PLWNT group or CBT group. The clinical scale evaluation was conducted at the end of the intervention (T_1_). All of the patients involved in this study signed an informed consent form. More detailed fundamental information of CFS patients is provided in our previously published protocol (34).

### Intervention

#### PLWNT Group

**Figure 1:**
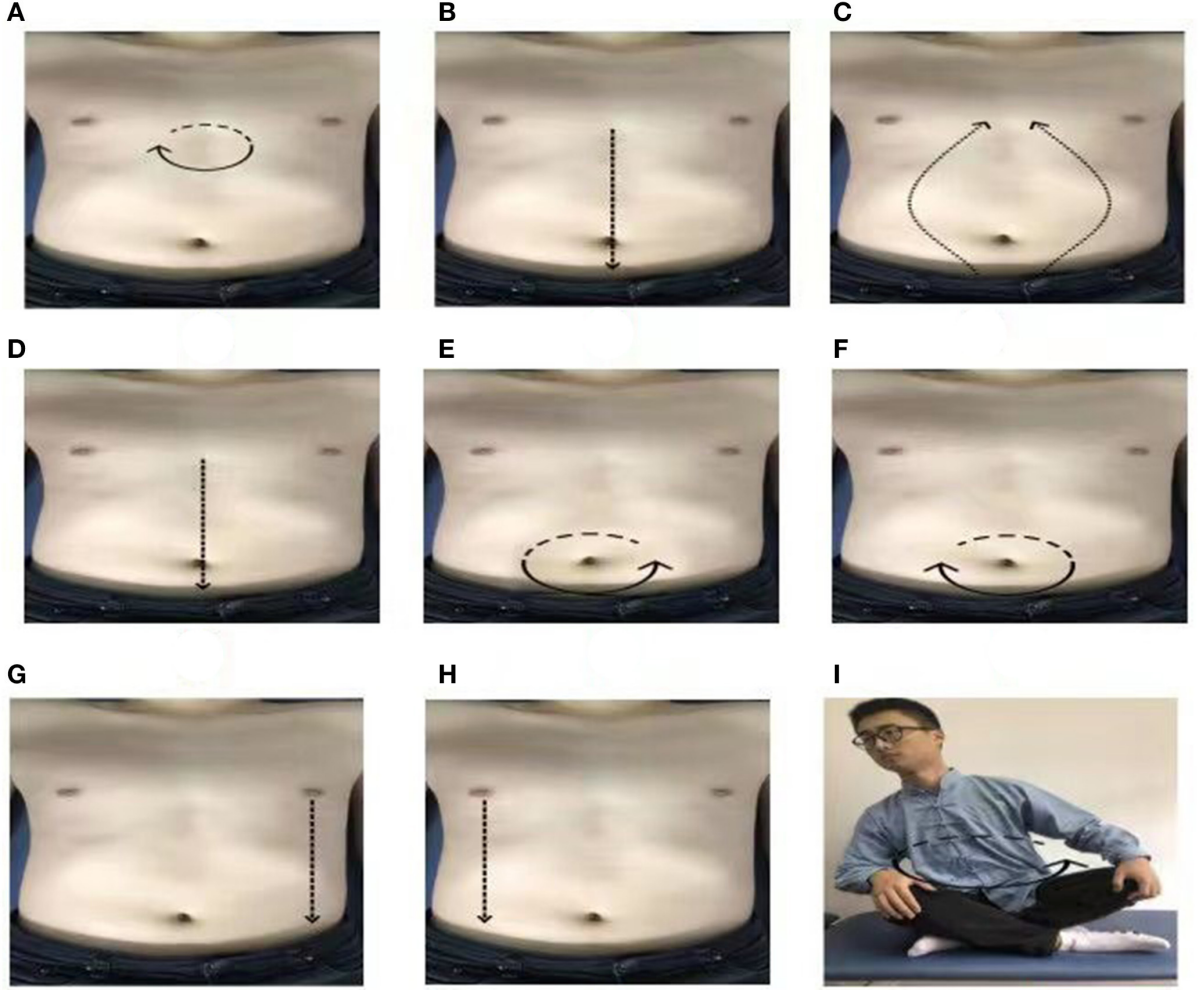
The postures of PLWNT (34). **(A)** Press and knead acupoint in Danzhong. **(B)** Rubbing from Danzhong Acupoint to Pubic Symphysis. **(C)** Rubbing from Pubic Symphysis to Danzhong Acupoint. **(D)** Pushing from Danzhong Acupoint to Pubic Symphysis. **(E)** The right hand massages the abdomen by the left circle. **(F)** The left hand massages the abdomen by the right circle. **(G)** Pushing with the right hand from the left breast to the groin. **(H)** Pushing with the left hand from the right breast to the groin. **(I)** Turn left and right. Every movements will be carried out 21 times. PLWNT, prolong life with nine turn method.

The PLWNT intervention program and operating standards refer to the Chinese general higher TCM compiled college textbook of Tuina and Qigong. Experienced qigong teachers at Shanghai University of TCM, who have been teaching qigong for at least 5 years, were placed in charge of the supervision of the exercise and corrected participants' exercise postures during the entire intervention period for 1 h every Sunday. The first 10 min of each session were for stretching and relaxation exercises as well as movement introductions and demonstrations. In addition, precautions were mentioned and participants' questions were answered. The subsequent 20 min were allotted for individual guidance and correction of actions. Finally, all of the participants practiced PLWNT for 30 min together. For the remaining 6 days of the week, all participants had to practice by themselves for 30 minat 6 o'clock every day at home, under the remote supervision of one of the directors. Their practice videos were required to be posted in a WeChat that it is similar to WhatsApp of all participants. If some of the participants found it inconvenient, videos could be sent privately to the study investigators. All participants were also asked to write down their feelings in the practice recording notebook after every exercise. Before we did the exercises, we gave the patient a 3-day training in the amount of abdominal stimulation, During the period, we let patients wear manual stimulation data gloves, the average amount of abdominal stimulation for the first eight rubbings was 0.5 ± 0.1 kg, and monitor the strength of the manual in real time in the LABVIEW2017 software, so that patients can feel the amount of stimulation. The entire practice process lasted for 12 weeks. The content of PLWNT qigong intervention was the same as in our previous research (34). The nine specific forms of manipulations are shown in Figure 1. The following are the three steps of qigong.

#### Step 1. Preparatory Position

During this step, the participant should relax their whole body, concentrate their thoughts, breathe evenly, place their tongue against the upper jaw, hold their Dantian with their mind, and progress through the exercise step by step.

#### Step 2. PLWNT's First Eight Types of Abdominal Massage

1. Press the Danzhong acupoint (under the xiphoid process) with the middle three fingers in both hands and make a circle 21 times from the left, within3 min.
2. With three fingers of both hands, rub down from the Danzhong acupoint and move to the pubic symphysis below the umbilicus. Repeat 21 times within 3 min.
3. With three fingers in both hands, rub up from the pubic symphysis from two sides back to the Danzhong acupoint until the hands are overlapped. Repeat this 21 times within 3 minutes.
4. With three fingers of both hands, push down from the Danzhong acupoint and push it straight to the pubic symphysis. Repeat 21 times within 3 min.
5. Rub the abdomen with the right hand from the left 21 times within 3 min.
6. Rub the abdomen with the left hand from the right 21 times within 3 min.
7. Place the left hand on the left side of the lower waist and kidney, with the thumb forward, and, using the four fingers supporting the back, gently pinch it; meanwhile, with three fingers of the right hand, push straight from the bottom of the left breast to the groin, and repeat this 21 times in 3 min.
8. Place the right hand on the right side of the lower waist and kidney, with the thumb forward, and, using the four fingers supporting the back, gently pinch it; additionally, with three fingers on the left hand, push straight from under the right breast to the groin, and repeat this 21 times in 3 min.

#### Step 3. Seated Rocking Method

Sit cross-legged, the participant should hold their hands up slightly and press them on the knees. The toes of both feet should be slightly bent. The participant should revolve the upper body clockwise 21 times and then counterclockwise 21 times.

#### CBT Group

Qualified CBT therapists [e.g., those with a diploma in CBT or other professionally accredited qualifications involving CBT as a major part of training (e.g., a clinical or counseling psychologist degree)] were invited to conduct CBT by giving lectures or psychological consultations on the prevention and treatment of CFS for 1 h each week. On the remaining 6 days of the week, all participants were required to listen to lectures on WeChat for 30 min every day. If some of the participants found it inconvenient, they were allowed to learn at their own pace using provided PowerPoints. Each participant was asked to write down their feelings in the practice recording notebook after each online session to ensure that the other conditions were the same as those of the PLWNT group. The entire practice process lasted for 12 weeks. Detailed information is available in the previously published protocol (34).

### Outcomes

Outcome evaluations included the basic characteristics of personal information, the detection of peripheral blood of CFS, the quality of sleep, mental and physical fatigue, and anxiety and depression symptoms. The patient's basic information was evaluated at baseline using relevant self-assessment scale and peripheral blood concentration to assess the primary and secondary outcomes after 12 weeks of intervention, including the Multi-dimensional Fatigue Inventory 20 (MFI-20), Pittsburgh Sleep Quality Index (PSQI), and Hospital Anxiety and Depression Scale (HADS).

### Primary Outcomes

#### MFI-20

The MFI-20 is widely used for CFS measurement of mental and physical fatigue (39), including a total of 20 items, including five dimensions of general fatigue, physical fatigue, mental fatigue, reduced activity, and reduced motivation. Each item can be scored on a scale of zero to five points, and the total possible score is 100 points. The higher the score is, the more severe the fatigue is. The MFI-20 was found to have good internal consistency (Cronbach's alpha = 0.89) and reliability (Pearson correlation of the total score = 0.73) (40).

### Secondary Outcomes

#### Overall Efficacy Evaluation

The overall efficacy evaluation was formulated with reference to the efficacy standard established by the “Discussion on the Curative Effect Standards for Diagnosis and Treatment of Chronic Fatigue Syndrome” (41, 42) and combined with the MFI-20 score. Full recovery was defined as the complete disappearance of the main clinical symptoms and concurrent symptoms and a reduction in MFI-20 score of more than 95%. A markedly effective result was defined as the disappearance of more than two-thirds of the main clinical symptoms and concurrent symptoms and a reduction in MFI-20 score of more than 70%. An effective result was defined as the disappearance of more than one-third of the main clinical symptoms and concurrent symptoms and a reduction in MFI-20 score of more than 30%. An ineffective result was defined as the disappearance of less than one-third of the main clinical symptoms and concurrent symptoms and a reduction in MFI-20 score of <30%. The total effective rate was the sum of the recovery rate, the markedly effective rate, and the effective rate.

#### PSQI

PSQI is a self-assessment questionnaire used to evaluate sleep quality. The scale consists of 24 items, with 19 self-reported items and five additional items rated by the director but not scored. The 19 items belong to one of the following seven subcategories: subjective sleep quality, sleep latency, sleep duration, habitual sleep efficiency, sleep disturbances, use of sleeping medication, and daytime dysfunction (43). The score range for each dimension is from zero to three points, and the total possible score is 21 points. The higher the score is, the worse the sleep quality is. The Cronbach's α coefficient of the PSQI was 0.68, and it increased to 0.78 after two components (medication use and daytime dysfunction) were removed. The PSQI has sufficient internal consistency (Cronbach alpha = 0.79), and test-retest reliability and validity (44, 45).

#### HADS

The HADS is used to evaluate the degree of anxiety and depression of patients. The scale consists of 14 items, including seven items that assess anxiety and seven items that assess depression. Scores for each item range from zero (nothing at all) to three points (the extreme form of each symptom) (46). The higher the total score is, the more severe the degrees of anxiety and depression are. The Cronbach's alpha reliability statistic of 0.70 for HADS is considered as the minimum acceptable criterion of instrument internal reliability (47, 48).

#### Peripheral Blood Biomarkers

NPY is an objective blood indicator of peripheral biomarkers for detecting sleep, anxiety, and depression in CFS (49). After the patients were grouped, non-fasting blood samples were collected. All blood samples in this study were sent to the clinical immunology laboratory of Yueyang Hospital of Integrated Traditional Chinese and Western Medicine, where the plasma was separated from the cells within 2 hafter collection, aliquoted into cryovials, and stored at −80°C until laboratory testing. The sample was only thawed once.

### Correlations

Possible determinants of the total scores of fatigue, sleep quality, and anxiety and depression at the end of treatment (T_1_) were investigated. The linear relationship among them was further explored.

### Adverse Events

We did our best to prevent and treat damage that may have been caused by this research. If an adverse event occurred during the clinical trial, including any discomfort, new changes in the condition, or any unexpected situation, details would be sent to the nearby Yueyang Hospital of Integrated Traditional Chinese and Western Medicine for review, where a medical expert committee determined whether the event was related to the study treatment or not. If the expert committee determined that the adverse event was related to treatment, the cost of treatment and corresponding financial compensation were provided to the participant. All expected and unexpected reactions reported by each participant were recorded on an adverse event reporting form, and all adverse events were followed up with until they were resolved. A score range of one to four points (1, definitely not related; 2, probably not related; 3, probably related, 4, definitely related) was used to indicate the degree of relationship between PLWNT treatment and adverse events.

### Statistical Analysis

The Statistical Package for the Social Sciences version 25.0 (IBM Corporation, Armonk, NY, USA) was used for statistical analysis. For measurement data, such as age and scale score, average value ± standard deviation ($\bar{X}\pm$ S) values were used. For measurement data conforming to normal distribution and homogeneity of variance test, paired-samples *t*-tests were used for comparisons before and after the test. For non-normally distributed measurement data, the Wilcoxon paired non-parametric test were used before and after the test, and *P* < 0.05 indicated that the difference was statistically significant. The Spearman correlation analysis was used to study the possible relationship between the MFI-20 and the clinical features of the PSQI and HADS scale scores.

## Results

**Figure 2:**
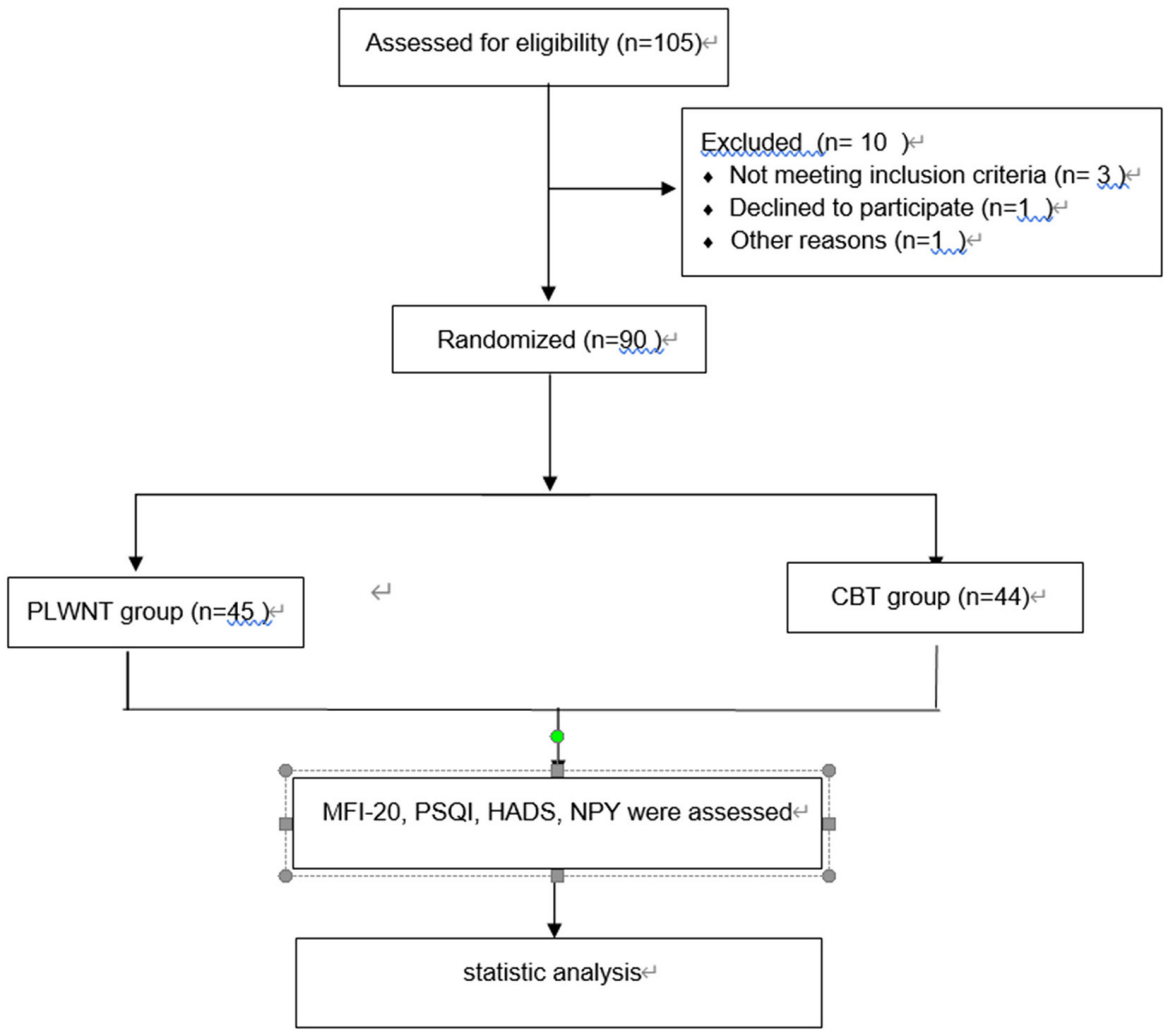
Recruitment flowchart.

A total of 90 participants who met the criteria were recruited in this study. They were randomly divided into a PLWNT group and CBT group, with 45 people placed in each group. Among them, one case in the CBT group withdrew due to shoulder fracture, which was a lost case. In the end, 89 participants completed the entire treatment plan. The process is shown in a flowchart (Figure 2).

### Demographic and Clinical Characteristics

The baseline characteristics of the PLWNT group and CBT group are shown in Table 1, which reveals that no difference existed in aspects such as the ratio of men to women, age, height, and education level between the two groups. Fatigue, sleep, and depression scale scores between the PLWNT and CBT groups were comparable, with *P*-values were all 0.05. These results indicated that the baseline values were relatively uniform. The participants were between 20 and 50 years of age. Most were female students in high schools or universities. Nearly half were married. They joined in the trial mainly due to referral by the hospital. In terms of MFI-20, PSQI, and HADS scores, patients who scored higher suffer more severe symptoms of fatigue, sleep disorders, and depression.

**Table 1:** Demographic and clinical characteristics of the patients.

	**PLWNT (*n* = 45)**	**Control (*n* = 44)**	***P*-vaule**
**Age (year)**	37.943 ± 11.344	37.343 ± 9.864	>0.05
**Weight (kg)**	59.804 ± 10.893	61.943 ± 12.061	>0.05
**Height (cm)** **Gender(M\F)** **Education(year)** **Married** **Single** **Divorce** **Clinical score** **MFI-20** General fatigue Physical fatigue Reduced activity Reduced- Motivation Mental fatigue **PSQI** Subjective sleep Sleep latency Sleep duration Habitual sleep Sleep disturbances Use of medicine Daytime function Total score **HADS** Anxiety Depression	163.514 ± 6.679 17\28 11.823 ± 3.25 21 23 1 10.556 ± 2.896 9.067 ± 3.962 8.622 ± 3.6,200 8.200 ± 4.257 10.022 ± 4.104 1.444 ± 0.724 1.333 ± 1.066 0.911 ± 0.821 0.644 ± 1.171 1.467 ± 0.625 0.200 ± 0.757 1.022 ± 0.657 6.756 ± 3.523 7.000 ± 3.855 6.844 ± 4.033	165.000 ± 7.376 19\25 11.232 ± 2.86 20 22 2 10.932 ± 2.872 10.614 ± 3.674 9.818 ± 3.611 8.091 ± 3.672 9.886 ± 3.357 1.545 ± 0.697 1.705 ± 0.904 1.023 ± 0.792 0.455 ± 0.730 1.614 ± 0.618 0.091 ± 0.473 1.273 ± 0.624 7.705 ± 2.953 7.682 ± 3.969 6.705 ± 3.927	>0.05 >0.05 >0.05 >0.05 >0.05 >0.05 >0.05 >0.05 >0.05 >0.05 >0.05 >0.05 >0.05 >0.05 >0.05 >0.05 >0.05 >0.05 >0.05 >0.05 >0.05

### Outcome Measurements

Table 2 suggests the total effective rate of the PLWNT and CBT groups. The results showed that the total effective rate of the PLWNT group was 62.22%, in which zero cases were cured, seven cases had markedly effective results, 21 cases had effective results, and 17 cases had ineffective results. The total effective rate of the CBT group was 50%, in which zero cases were cured, zero cases had markedly effective results, 22 cases had effective results, and 22 cases had ineffective results. There was a significant difference between the two groups (*P* < 0.05), indicating that the treatment effectiveness of the PLWNT group in the management of CFS was greater than that of the CBT group.

**Table 2:** Efficacy evaluation of PLWNT group and CBT group after 12 weeks of intervention (Cases/effective rate).

**Group**	**Cases**	**Cases/cured (%)**	**Cases/markedly effective (%)**	**Cases/effective (%)**	**Cases/no effective (%)**	**Total effective (%)**
PLWNT	45	0/0	7/15.56	21/46.67	17/37.78	62.22
CBT	44	0/0	0/0	22/50	22/50	50

Table 3 shows the changes in the primary and secondary scores of the MFI-20, PSQI, and HADS scales from T_0_ to T_1_ in the two groups. The results indicate that the primary outcome of MFI-20 significantly decreased after 12 weeks of intervention in the PLWNT and CBT groups, which show a statistically significant difference (*P* < 0.001). Thus, in both groups, conditions improved in terms of general fatigue, physical fatigue, reduced activity, reduced motivation, and mental fatigue. As for the secondary outcomes, the overall scores of the PSQI and HADS scales had also improved when comparing T_1_ to T_0_ in the PLWNT and CBT groups. The comparison of HADS scores within the groups showed statistical significance after PLWNT and CBT intervention. According to the PSQI results, significant improvements in overall sleep quality, subject sleep quality, sleep latency, sleep duration, sleep disturbance, and daytime dysfunction after intervention were noted (*P* < 0.05). However, in terms of habitual sleep efficiency and sleep medicine usage, there was no statistically significant difference between the groups (*P* > 0.05). The total scores and the average value of each score considering the changes of MFI-20, PSQI, and HAS scales in the PLWNT group were greater than those in the CBT group, although there was no statistical significance between the groups.

**Table 3:** Shows the changes primary and secondary outcomes in fatigue, sleep, anxiety and depression scores measured by MFI-20, PSQI, and HADS scales from T0 to T1.

	**CBT (*****n*** **=** **44)**			**PLWNT (*****n*** **=** **45)**			***PLWNT vs. CBT***
	**T_**0**_**	**T_**1**_**	***Within***	**T_**0**_**	**T_**1**_**	***Within***	***Between***
	**($\overset{-}{X}$ ±S)**	**($\overset{-}{X}$ ±S)**	***group p***	**($\overset{-}{X}$ ±S)**	**($\overset{-}{X}$ ±S)**	***group P***	***group P***
**Primary outcome: MFI-20**							
General fatigue	10.932 ± 2.872	7.341± 3.403	0.000*	10.556 ± 2.896	6.511 ± 2.897	0.000*	0.516
Physical fatigue	10.614 ± 3.674	6.727± 3.172	0.000*	9.067± 3.962	4.511 ± 2.785	0.000*	0.435
Reduced activity	9.818 ± 3.611	6.545± 3.238	0.000*	8.622± 3.6, 200	4.0.911 ± 2.502	0.000*	0.593
Reduced motivation	8.091 ± 3.672	5.114± 2.508	0.000*	8.200± 4.257	4.644 ± 2.854	0.000*	0.482
Mental fatigue	9.886 ± 3.357	6.318± 2.752	0.000*	10.022 ± 4.104	5.578 ± 2.759	0.000*	0.282
**Secondary outcome: PSQI**							
Total score	7.705 ± 2.953	5.295± 2.378	0.000*	6.756± 3.523	4.200 ± 2.085	0.000*	0.828
Subject sleep quality	1.545 ± 0.697	1.159± 0.079	0.002*	1.444± 0.724	0.956 ± 0.424	0.000*	0.532
Sleep latency	1.705 ± 0.904	1.386± 0.813	0.021*	1.333± 1.066	0.933 ± 0.720	0.011*	0.685
Sleep duration	1.023 ± 0.792	0.409± 0.622	0.000*	0.911± 0.821	0.200 ± 0.405	0.000*	0.609
Habitual sleep efficiency	0.455 ± 0.730	0.364± 0.718	0.439	0.644± 1.171	0.378 ± 0.912	0.209	0.142
Sleep disturbance	1.614 ± 0.618	1.205± 0.461	0.000*	1.467± 0.625	1.022 ± 0.452	0.000*	0.783
Sleep medicine using	0.091 ± 0.473	0.091± 0.291	1.000	0.200± 0.757	0.089 ± 0.468	0.168	0.326
Daytime dysfunction	1.273 ± 0.624	0.682± 0.601	0.000*	1.022± 0.657	0.356 ± 0.609	0.000*	0.593
**Secondary outcome: HADS**							
Anxiety	7.682 ± 3.969	5.727± 3.083	0.000*	7.000± 3.855	4.111 ± 2.113	0.000*	0.198
Depression	6.705 ± 3.927	4.705± 3.069	0.001*	6.844± 4.033	3.444 ± 2.563	0.000*	0.105

* *P < 0.05*.

The distribution of plasma NPY values between the two groups is shown in Table 4. The analysis showed that NPY decreased after PLWNT intervention (126.17 ± 16.88 vs. 123.55 ± 17.14) but increased significantly after CBT treatment (142.99 ± 17.86 vs. 201.01 ± 22.83). Compared to the CBT group (201.01 ± 22.83), the plasma NPY of CFS patients in the PLWNT group (123.55 ± 17.14) was decreased, and there was a statistical difference between the groups (*P* = 0.008).

**Table 4:** The distribution of plasma NPY values of the three groups.

	**PLWNT group (*n* = 45)**	**CBT group (*n* = 44)**	***P***
T0	126.17 ± 16.88	142.99 ± 17.86	0.498
T1	123.55 ± 17.14	201.01 ± 22.83	0.008*
*d*-value	−2.62 ± 23.77	58.03 ± 28.98	0.110

* *P < 0.05*.

### Correlations

**Figure 3:**
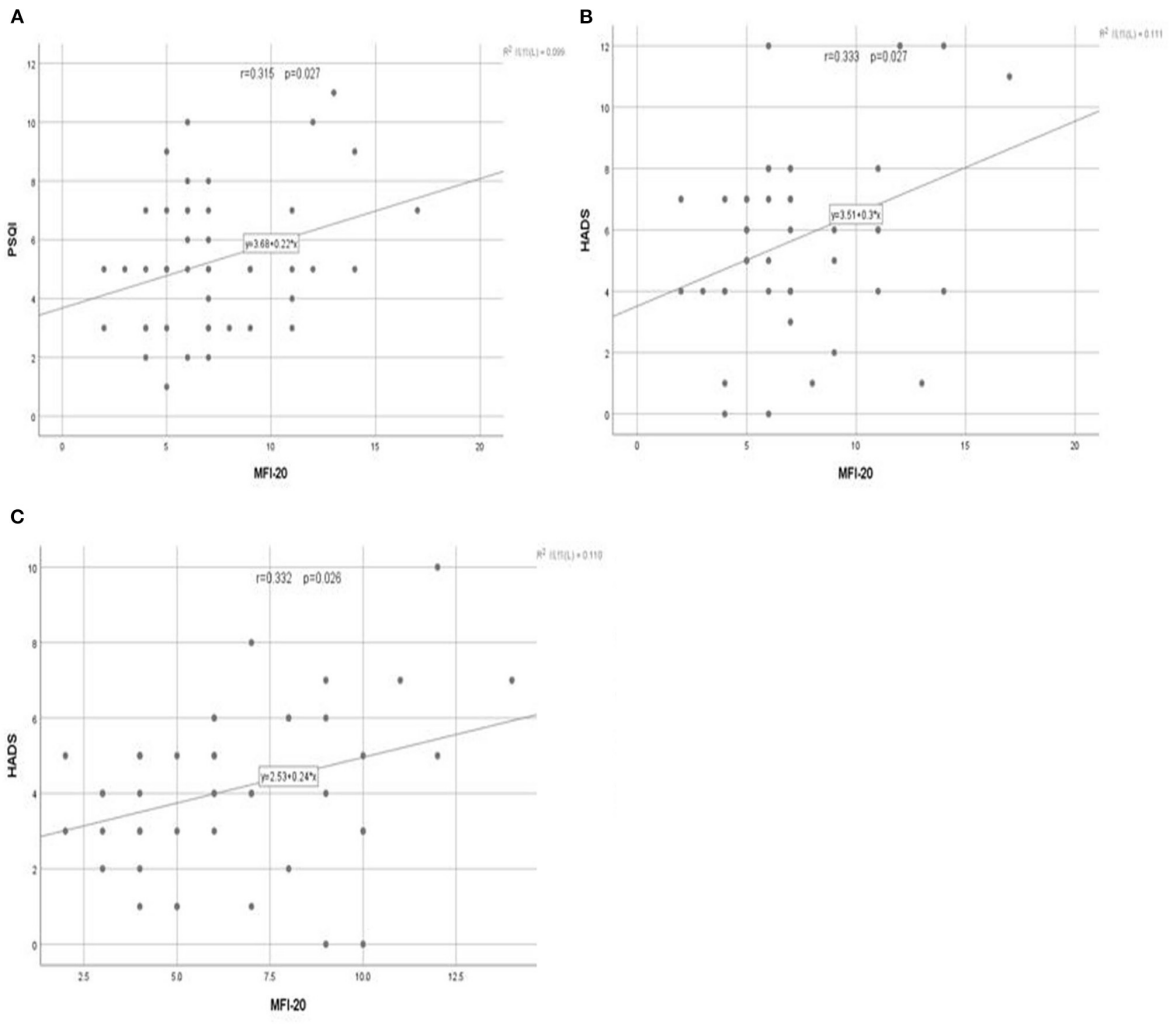
The linear relationship between the total score of fatigue and the total score of sleep and anxiety at the end of treatment. The relationship between the **(A)** total score of fatigue and the total score of sleep after PLWNT exercise. **(B)** total score of fatigue and the total score of anxiety after PLWNT exercise. **(C)** total score of fatigue and the total score of anxiety after CBT intervention.

Fatigue was the primary result of the study, and the treatment groups underwent additional correlation analysis to compare between the primary and secondary outcomes. Pearson's correlation coefficient was used to explore the relationship between fatigue and sleep quality as well as fatigue and anxiety. In the PLWNT group, fatigue level was positively correlated with sleep quality (*r* = 0.315) and anxiety level (*r* = 0.333) (*P* < 0.05). However, fatigue level was only related to anxiety level (*r* = 0.332) (*P* < 0.05) and showed no correlation with sleep quality in the CBT group (*P* > 0.05). The specific results are shown in Figure 3.

### Adverse Events

Table 5 displays data from a total of six participants who reported six adverse events in our study. Among them, two cases were determined to be definitely or probably related to the exercises, with mild symptoms caused by improper massage manipulations on the abdomen. Except for one patient who chose to quit the study due to shoulder fracture, all other participants continued with treatment after their adverse events had been properly dealt with. There was no significant difference in AE statistics between the two groups (*p* = 0.096).

**Table 5:** Adverse event.

**Symptom**	**Group**	**Patients**	**Start date**	**End date**	**Relationship**	**Treatment**	**Action related**	**Outcome**
		**numbers**					**to intervention**	
Leg pain	PLWNT	1	2019/02/24	2019/03/03	Probably	None	No change	Cured
Shoulder	CBT	1	2019/03/09	2019/03/10	Definitely not	Medication	Withdraw	Unknown
Dizziness	PLWNT	1	2019/05/12	2019/05/12	Probably not	Measure blood pressure	No change	Cured
fracture						and eat breakfast		
Left thumb	PLWNT	1	2019/07/21	2019/08/04	Definitely not	None	No change	Cured
pain								
Diarrhea	PLWNT	1	2019/08/11	2019/08/12	Probably not	Catch cold	No change	Cured
Chest pain	PLWNT	1	2019/08/15	2019/08/29	Definitely	Relieve acupoint	No change	Cured
						stimulation		

## Discussion

This study was performed to evaluate the efficacy of PLWNT on fatigue, sleep disorders, and depression symptoms in CFS patients and to discern the linear correlation between fatigue and sleep quality as well as fatigue and depression levels. As the results show, the levels of fatigue, anxiety, and depression as well as sleep quality of CFS patients were significantly improved after PLWNT or CBT intervention, and the overall efficacy of the two groups was effective after intervention with no significant difference between the groups. The self-reported sleep time increased to the minimum of 7 h after PLWNT intervention, which is the amount recommended in many guidelines. However, no improvement was reported from T_0_ to T_1_ in terms of the habitual sleep efficiency or sleep medicine usage. These findings must be interpreted with caution, because the levels of fatigue, anxiety, depression and sleep quality did not have significant differences between the PLWNT group and the CBT group due to the relatively small between-group effect size.

CFS is characterized by long-term, unexplainable fatigue (50), and it can be treated by PLWNT qigong. Previous studies have indicated the efficacy of moxibustion, auricular acupoint pressure, Chinese medicine, and other therapies for the alleviation of fatigue symptoms in CFS patients (23, 51, 52). Patients who suffer from fatigue cannot carry out or maintain a physiological activity. If not treated properly and promptly, the symptoms can deteriorate into chronic fatigue of a certain degree (53, 54). Studies have confirmed that the body's ability to scavenge oxygen free radicals decreases when it is in a fatigue state, and, if the free radicals in the body cannot be eliminated in due time, the fatigue will worsen (55, 56). PLWNT qigong is an effective treatment for CFS. It incorporates eight kinds of massage manipulations on the abdomen and a kind of upper body shaking method. The active mechanisms that relieve fatigue symptoms may be to render the whole-body skeletal muscles, especially those of the upper limbs, in a state of relaxing limb activity; enhancing the body's antioxidant enzyme activity; removing oxygen free radicals; and stabilizing the body's environment (57). It has been reported in a previous study (58) that traditional qigong exercises can increase the activity of the diaphragm and abdominal muscles, strengthen peripheral skeletal muscle function, and improve fatigue symptoms. These may be speculated that the main reason for the improvements in reduced activity and reduced motivation shown in the MFI-20 scores of CFS patients after PLWNT intervention in this study. In addition, patients with CFS fatigue often have problems with immune dysfunction, which may be one of the causes of persistent fatigue (59, 60). A previous study has reported that abdominal massage manipulations can enhance the body's immunity by clearing lactic acid from the blood after fatigue, improving the patient's autonomic nerve function, and bringing the sports center into a benign state of excitement (61). This may be the main mechanism by which PLWNT relieves mental fatigue and physical fatigue.

Sleep quality, subjective sleep quality, sleep latency, sleep duration, sleep disturbance, and daytime dysfunction of CFS patients were significantly improved after PLWNT intervention. These findings are consistent with the results of a previous study results following the conduct on Baduanjin qigong, which improved the sleep latency and sleep duration in CFS patients but did not improve subjective sleep quality or sleep disturbance (62). In this study, the effect of PLWNT qigong on the multi-dimensional improvement of sleep quality may also be related to the abdominal massage manipulations. Studies have indicated that abdominal massage manipulations can regulate nerve conduction connections through the brain–gut axis, stimulate nerve-conduction pathways and nerve-emotional pathways through internal organs, and strengthen the connection between the abdomen and cranial nerves to regulate fatigue and sleep in CFS patients (63, 64). It is worth noting that no improvement was found in terms of the habitual sleep efficiency or the use of sleep drugs by the participants in this study. These findings are consistent with several previous studies on CFS, which reported that CFS patients are more likely to be awakened and have a longer sleep latency than healthy people (65, 66). Therefore, it is speculated that the lack of improvement in habitual sleep efficiency may be caused by sleep interruptions or a prolonged sleep latency, leading to more time spent in bed. However, the fact that the present study excluded all subjects taking medications may be why the difference in the use of sleep drugs was not significant.

In our study, the anxiety and depression levels of CFS patients decreased significantly after PLWNT intervention. Baduanjin qigong exercise has been reported to have antidepressive effects in women with CFS-like diseases (67). The limbic system of the human brain is responsible for emotional regulation. Therefore, anxiety and depression may be correlated with dysfunction of the brain network connection the limbic system and cortex (68). In some studies, antidepressants are used to increase serotonin levels and reduce cortisol secretion in the brain to deal with the anxiety and depression of CFS patients, but these drugs have side effects, such as headache, sleep disturbance, changes in cardiovascular function, and bone loss (69). It has been reported that rubbing the abdomen can increase serotonin and endorphins to activate the spinal cord and subcortical nucleus activity to reduce the levels of anxiety and depression (70). PLWNT may be used to relieve anxiety and depression symptoms through abdominal pressing and rubbing.

Improvements in fatigue, sleep, and anxiety symptoms were directly related to the amount of qigong exercises, which was consistent with previous studies of other types of qigong (71, 72). In this study, Pearson's correlation coefficient indicated that the fatigue level was positively correlated with the anxiety level and sleep quality. A new study conducted by Russell et al., in which the sleep quality of 27 adult CFS patients (73) was evaluated using a sleep–wake diary and activity recorder, had similar results, indicating that sleep disorders induce a more serious level of fatigue. Similarly, in the case of perception of external stress and fatigue, the hypothalamic–pituitary–adrenal axis is activated and overactive, and a large amount of glucocorticoids will be released into the body to perceive anxiety (74). This shows that fatigue is closely related to sleep and anxiety symptoms, so maybe we need to increase the time of PLWNT exercise or make it as a daily exercise to relieve fatigue so as to gain more benefits.

Enzyme-linked immunosorbent assay blood indicators were used to objectively show the relationship between NPY and CFS symptoms of fatigue, sleep, anxiety, and depression after PLWNT intervention, and the findings were consistent with those of some previous studies (75, 76), which showed that different forms of exercise can reduce the level of expression of NPY and inhibit the development of the disease. Another study of NPY comparing CFS patients with HC patients showed that NPY was significantly increased in CFS patients, which means that the reduction of NPY level leads to the relieve of anxiety and depression (49). This finding is consistent with our results that the relief of anxiety and depressive symptoms after PLWNT intervention may be associated with significantly lower levels of NPY in our study. However, there are many factors affecting NPY, and the content of NPY in different diseases is different, one study showed no overall difference in NPY concentrations before and after the intervention (77). So the blood level of NPY is whether dependent to other diseases/parameters is still unclear. In addition, the cause of the increased NPY results after CBT treatment cannot be determined at present, and it may be related to the intervention method.

In the future, we will conduct more in-depth studies to further explore the possible mechanisms by which NPY levels affect CFS-related symptoms after PLWNT and CBT interventions.

This study did not report adverse events due to exercise, and only one case withdrew due to her own reasons. This shows that PLWNT exercises guided by professionals can be safely adopted by CFS patients. In general, our research indicates that PLWNT represents an alternative and more acceptable form of exercise for CFS patients. This has great significance in health care for CFS patients, who may not be able to perform traditional exercises due to physical limitations or comorbidities. PLWNT is a mild, low-intensity form of exercise, usually accepted by CFS patients, and it is an advantageous method for them to comply with the World Health Organization's physical activity recommendations.

However, there are some limitations in this study. First of all, the study's experimental design itself had potential limitations. Ideally, participants should remain uninformed about the intervention; however, it is difficult to do this in non-drug trials. Second, patients over 60 years old are excluded in this study. Therefore, the results should not be generalized to the elderly over 60 years old. Future research will be performed with interventions controlling all non-specific factors so as to better understand the specific efficacy of PLWNT. Despite these limitations, to our knowledge, this study is the first large-scale randomized controlled trial to prove the beneficial effects of PLWNT on CFS. Another obvious advantage is that this study reports all the adverse events in detail. We described the adverse symptoms, the time of appearance and disappearance, the relationship between the adverse events and the trial, and the related treatments and results. During the clinical trial, no one quit due to an adverse event. The safety of qigong treatment has been reported on before (78–80) and has now been demonstrated in our study.

## Conclusion

In summary, PLWNT has a positive effect in the treatment of fatigue, sleep disorders, and anxiety and depression symptoms of CFS patients, and fatigue level is positively correlated with sleep quality as well as anxiety and depression levels. PLWNT can be considered as a treatment option for CFS patients, but more rigorous research is needed to provide clear evidence. Future studies will be carried out using a larger sample size for further in-depth research to determine the effective frequency and intensity of PLWNT qigong intervention in the treatment of CFS. This study has significant translational significance because our findings will support the inclusion of PLWNT in the global physical activity guidelines for CFS patients.

## Data Availability Statement

The raw data supporting the conclusions of this article will be made available by the authors, without undue reservation.

## Ethics Statement

The studies involving human participants were reviewed and approved by Yueyang Hospital of Integrated Traditional Chinese and Western Medicine (Ethics Approval No. 2018-043). The patients/participants provided their written informed consent to participate in this study. Written informed consent was obtained from the individual(s) for the publication of any potentially identifiable images or data included in this article.

## Author Contributions

FX, YY, FY, and JX contributed to data analysis and interpretation. FX, YY, CG, JX, and FY drafted the revised manuscript and conceived and received the project. All authors contributed to the article and approved the submitted version.

## Funding

This work was supported by the National Natural Science Foundation of China under Grants 81774443 and 82105038 and the National Key Technology R&D Program of China Grant 2017YFC1703301.

## Conflict of Interest

The authors declare that the research was conducted in the absence of any commercial or financial relationships that could be construed as a potential conflict of interest.

## Publisher's Note

All claims expressed in this article are solely those of the authors and do not necessarily represent those of their affiliated organizations, or those of the publisher, the editors and the reviewers. Any product that may be evaluated in this article, or claim that may be made by its manufacturer, is not guaranteed or endorsed by the publisher.

## Abbreviations

CFS: Chronic fatigue syndrome
PLWNT: Prolong life with nine turn method
IACFS: International association of chronic fatigue syndrome
CBT: Cognitive behavioral therapy
TCM: Traditional chinese medicine
MFI-20: Multidimensional Fatigue Inventory-20
PSQI: Pittsburgh sleep quality index
HADS: Hospital anxiety and depression scale
HPA: Hypothalamic-pituitary-adrenal.
