# Supplementary material for: The Qigong of Prolong Life With Nine Turn Method Relieve Fatigue, Sleep, Anxiety and Depression in Patients With Chronic Fatigue Syndrome: A Randomized Controlled Clinical Study
Source: Front Med (Lausanne). 2022 Jun 30;9:828414. doi: 10.3389/fmed.2022.828414 (PMC9280429; doi:10.3389/fmed.2022.828414)
Supplement: Supplementary file 1 [file Data_Sheet_1.PDF]

Chronic Fatigue Syndrome
Myalgic Encephalomyelitis

Primer for Clinical Practitioners
2014 Edition

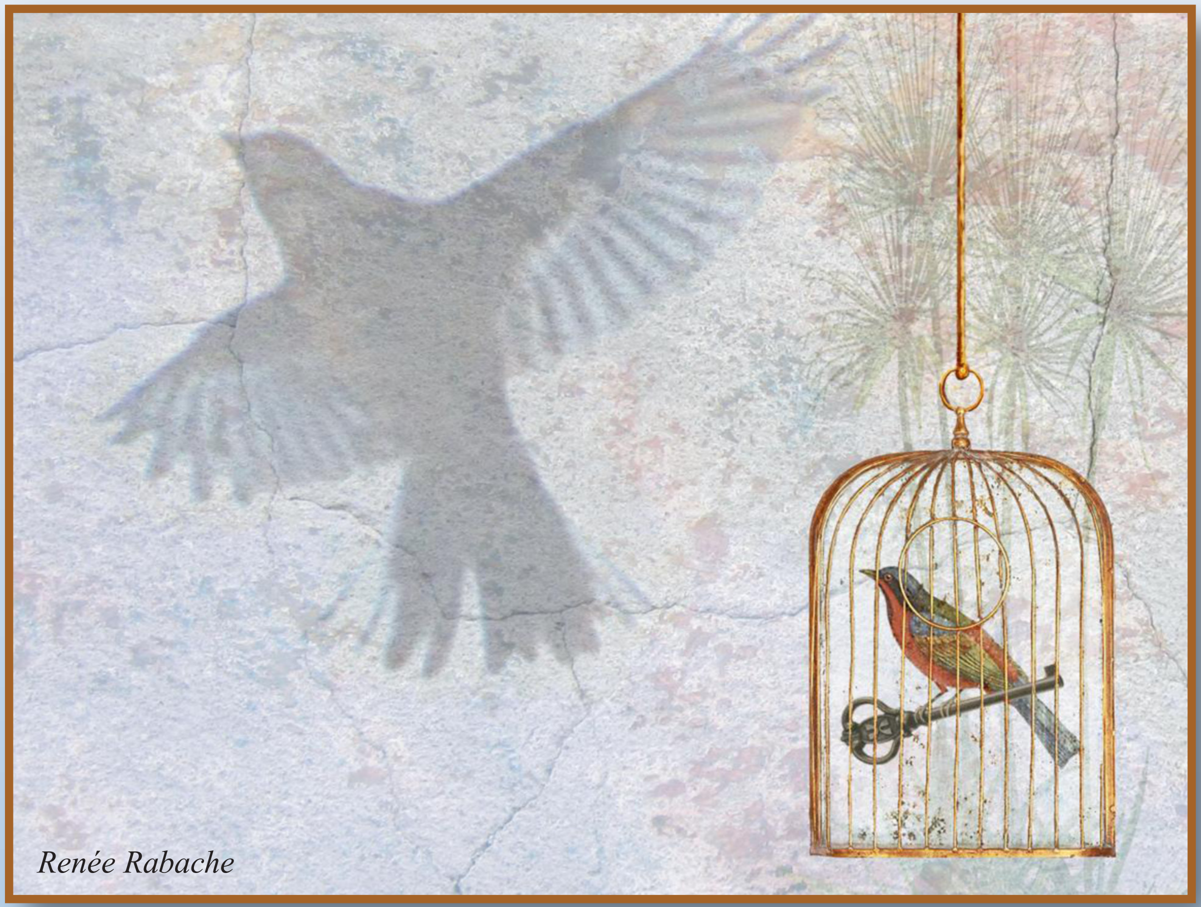

Renée Rabache

International Association for
Chronic Fatigue Syndrome/Myalgic Encephalomyelitis
IACFS/ME

ME/CFS: A Primer for Clinical Practitioners

Members of the IACFS/ME Primer Writing Committee

Fred Friedberg, Ph.D.
Chairperson
Stony Brook, New York, USA

Lucinda Bateman, B.S., M.D.
General Internal Medicine
Salt Lake City, Utah, USA

Alison C. Bested, M.D. F.R.C.P.C.
Haematological Pathologist
Vancouver, BC. Canada

Todd Davenport, D.P.T., O.C.S.
Physical Therapy
Stockton, California, USA

Kenneth J. Friedman, Ph.D.
Physiology/ Natural Sciences
Castleton, Vermont, USA

Alan Gurwitt, M.D.
Psychiatry
Newton Highlands, Massachusetts, USA

Leonard A. Jason, Ph.D.
Clinical-Community Psychology
Chicago, Illinois, USA

Charles W. Lapp, M.D.
Primary Care
Charlotte, North Carolina, USA

Staci R. Stevens, M.A.
Exercise Physiology
Ripon, California, USA

Rosemary A. Underhill, M.B., B.S.
Physician, Researcher
Palm Coast, Florida, USA

Rosamund Vallings, M.B., B.S.
Primary Care
Howick, New Zealand

Acknowledgements

We gratefully acknowledge the generous support of Chase Community Giving and Hemispherx Biopharma in the production of this primer. The primer committee also wishes to express its gratitude to: Lily Chu, M.D., Eulalia Benejam Cobb, Barbara B. Comerford Esq., Lucy Dechene, Ph.D., Pat Fero, Mary Ann Fletcher Ph.D., Nancy G. Klimas, M.D., Susan M Levine, MD., Denise Lopez-Majano, The Massachusetts CFIDS/ME & FM Association, Lydia Neilson, James M. Oleske M.D., Ellen V. Piro, and Eleanor Stein, M.D. for their thoughtful reviews of an earlier draft of this primer. We also thank Renée Rabache for donating the cover art for this primer.

Contact information

IACFS/ME, 27 N. Wacker Drive, Suite 416, Chicago, IL 60606 www.iacfsme.org Email: Admin@iacfsme.org

Revised July 2014

ME/CFS: A Primer for Clinical Practitioners

Conflicts of interest statement

The IACFS/ME received a $10,000 donation from Hemispherx, the maker of Ampligen® (a possible treatment for ME/CFS), which supported this primer. Charles Lapp is a Hemispherx principal investigator in Ampligen® studies and has a small amount of stock in the company. Lucinda Bateman has been a principal investigator in Hemispherx Ampligen® studies for 10 years. All other authors declared no conflicts of interest.

Disclaimer

This primer was developed by consensus among members of the primer committee who have made considerable effort to ensure that the information is accurate and up to date. Statements, opinions and study results published in this primer are those of the individual authors and the studies cited, and do not necessarily reflect the policy or position of the IACFS/ME. The IACFS/ME provides no warranty, express or implied, as to the accuracy or reliability of all the contents of this primer. The recommendations contained in any part of this primer do not indicate an exclusive course of treatment or course of action. Nothing contained in this primer should serve as a substitute for the medical judgment of a treating provider.

ME/CFS: A Primer for Clinical Practitioners

FOREWORD

About 25 years ago, modern medicine began to seriously study the illness we now call Chronic Fatigue Syndrome—also known as Myalgic Encephalomyelitis (ME/CFS).

In the United States, the National Institutes of Health and the Centers for Disease Control and Prevention have conducted research in their laboratories and funded research elsewhere. The International Association for CFS/ME (IACFS/ME) has organized eleven international conferences at which scientists from all over the world have presented thousands of research studies.

What has 25 years of research taught us? Twenty-five years ago we had no idea of the underlying pathophysiology of this illness. Worse than that, we did not even know if there *were* any underlying biological abnormalities in the illness. Indeed, some clinicians and scientists argued that the illness was probably psychological, and some even argued that it was a fabrication: patients were imagining symptoms that had no physiological basis.

For those of us who are practicing physicians, this was a frustrating situation. We had little knowledge, and no proven tools, with which to try to help patients who came to our office.

In my view, research of the past 25 years has identified many underlying biological abnormalities that are present more often in patients with ME/CFS than in healthy controls subjects or in subjects with other fatiguing illnesses, including depression, multiple sclerosis and Lyme disease.

Neurological abnormalities. Brain imaging studies with SPECT, PET and MRI have found abnormalities in both white and gray matter. Cognitive testing has confirmed problems that are independent of any coexisting psychological disorder. One group has reported a "signature" using EEG data that distinguishes patients with ME/CFS from patients with depression and from healthy subjects. Neuroendocrine studies have identified abnormalities in several hypothalamic endocrine releasing hormone axes, abnormalities that often are the opposite of what is seen in major depression. Studies of spinal fluid proteins have found unique patterns, and spinal fluid concentrations of lactic acid (and, hence, pH) are abnormal. Finally, many studies have identified abnormalities of the autonomic nervous system in patients with ME/CFS.

Energy metabolism. A growing body of evidence indicates that energy metabolism and mitochondrial function are impaired in many patients with ME/CFS. The basis for such abnormalities remains undetermined, but chronic viral infection and chronic immune activation are both proven causes of such abnormalities in other diseases.

Infectious triggers. Many (but not all) patients state that their illness began suddenly, with an infectious-like illness. There is good evidence that ME/CFS can follow in the wake of several different viral and bacterial infections. Indeed, it seems unlikely that a single novel infectious agent will prove to be a cause of the great majority of cases. Also, there is evidence that several viruses that produce latent, life-long infection in many humans may be reawakened or reactivated in ME/CFS, although it is unclear if this is the cause or the effect of the illness.

Immune activation. Many studies have found evidence of chronic T cell activation. A recent study of the drug rituximab provides indirect evidence for chronic B cell activation, as well.

Genetic component. Twin studies, studies of HLA antigens, and some gene sequencing studies indicate that ME/CFS—like most illnesses—has an underlying genetic component.

Implications for practice. Despite the substantial progress that has been made in understanding the underlying biology of ME/CFS, we still don't have a sufficiently accurate diagnostic test, or a proven treatment. What we can tell patients is that:
1) Research is uncovering what goes wrong in the body; 2) Many laboratories are working on developing diagnostic tests, and on testing treatments suggested by our growing understanding of how ME/CFS affects the body.
In this Primer, the collected wisdom of many experienced clinicians and clinician-scientists has

been gathered. Here, you'll find advice on how to diagnose ME/CFS, and on therapies that appear to be beneficial, although not curative. I think you will find it useful.

Anthony L. Komaroff, M.D.
The Simcox-Clifford-Higby Professor of Medicine, Harvard Medical School
Senior Physician, Brigham & Women's Hospital

ME/CFS: A Primer for Clinical Practitioners

Table of Contents

Preface 6
1 Introduction and Overview 6
1:1 Nomenclature 6
1:2 Epidemiology 6
1:3 Diagnosis 7
1:4 Presentation and course of Illness 7
1:5 The health practitioners role in diagnosis and management 7
2 Etiology 8
2:1 Predisposing factors 8
2:2 Precipitating and causal factors 8
3 Pathophysiology 8
3:1 Immune system abnormalities 8
3:2 Neuroendocrine dysregulation 9
3:3 Brain abnormalities 10
3:4 Cognitive impairment 10
3:5 Autonomic dysfunction/Cardiovascular abnormalities 10
3:6 Mitochondrial/energy production abnormalities 10
3:7 Gene studies 11
4 Clinical diagnosis 11
4:1 Patient history 11
Diagnostic worksheet 12
4:2 Physical examination 14
4:3 Laboratory tests 14
4:4 Differential diagnosis 14
4:5 Exclusionary medical conditions 15
4:6 Co-existing medical conditions 16
4:7 Differentiating between depressive/anxiety disorders and psychological reactions secondary to ME/CFS 16
5 Management/Treatment 17
5:1 Approach to treatment 18
5:2 Sleep 18
5:3 Pain 19
5:4 Fatigue and post-exertional malaise 20
Activity and exercise 20
Managing post-exertional symptoms, pacing and the energy envelope 21
5:5 Cognitive problems 22
5:6 Managing depression, anxiety and distress 23
5:7 Cognitive behavioral therapy 23
5:8 Management of related conditions 24
Orthostatic intolerance and cardiovascular symptoms 24
Gastrointestinal problems 24
Urinary symptoms 24
Allergies 24
Multiple chemical sensitivities 24
Infections and immunological factors 24
5:9 Dietary management 25
5:10 Alternative and complementary approaches 26
5:11 Prognosis 26
5:12 Follow-up 27
6 Clinical concerns 27
6:1 Lowest functioning patients 27
6:2 Pregnancy 28
6:3 Gynecological problems 29
6:4 Pediatric ME/CFS 29
6:5 Immunizations 30
6:6 Blood and tissue donation 30
6:7 Recommendations prior to surgery 30
7 References 31
Appendices
A 1994 International research case definition (Fukuda et al) worksheet 36
B Pediatric case definition worksheet 37
C Functional capacity scale 38
D Activity log 39
E Recommendations prior to surgery 41
F Fact sheet on ME/CFS 43
Index 45

ME/CFS: A Primer for Clinical Practitioners

PREFACE

This primer has been written for the clinical practitioner. Our goal is to provide the information necessary to understand, diagnose, and manage the symptoms of chronic fatigue syndrome — also known as myalgic encephalomyelitis (ME/CFS). The text was developed by consensus of the primer committee. The authors have made considerable efforts to ensure that the information provided is accurate and up to date. Since the extant literature does not adequately describe the nature and treatment of this illness, this document is written as a primer and is not "clinical practice guidelines" as recently redefined.* Where published studies are lacking, our recommendations are based on the clinical expertise of our experienced practitioners. Our hope is that you find the primer to be a useful adjunct to your practice and a worthy companion to your reference library.

Periodic updates will be available on our website: www.iacfsme.org

*http://www.guideline.gov/about/inclusion-criteria.aspx

---

1. INTRODUCTION & OVERVIEW

The terms chronic fatigue syndrome and myalgic encephalomyelitis (ME/CFS) describe a complex physical illness characterized by debilitating fatigue, post-exertional malaise, pain, cognitive problems, sleep dysfunction and an array of other immune, neurological and autonomic symptoms.[1] The key feature of the syndrome, post-exertional malaise, is the exacerbation of symptoms following minimal physical or mental activity, which can persist for hours, days or even weeks. Rest and sleep produce only modest relief of fatigue and the other symptoms. The illness is also characterized by substantially reduced physical and/or cognitive functioning.

Although ME/CFS is a physical illness, secondary psychological symptoms may be present as in many other chronic illnesses.

1:1 Nomenclature

The term myalgic encephalomyelitis (ME) was coined in 1956 to describe a well-documented cluster outbreak of a fatiguing illness in London, England. The name chronic fatigue syndrome (CFS) was proposed following the investigation of a cluster outbreak of a similar fatiguing illness in Nevada (USA) in 1984. CFS replaced the preliminary name, chronic Epstein-Barr virus syndrome, because clinical studies were unable to confirm Epstein-Barr virus as the putative cause. The name chronic fatigue syndrome has been criticized as being vague and trivializing of the illness.[2] CFS has also been confused with the common non-specific complaint of chronic fatigue. Other less common names for the illness are myalgic encephalopathy and chronic fatigue immune dysfunction syndrome (CFIDS). The World Health Organization classifies myalgic encephalomyelitis as a disease of the central nervous system (G93.3.).[3] A similar illness, post-viral fatigue syndrome (PVFS), describes the lingering of fatigue subsequent to a viral infection.

The name ME is more commonly used in Europe and Canada, while the CFS term is more often used in the USA and Australia. Different but overlapping case definitions have been published for each of the two terms. Most research studies use "CFS" because a specific case definition (Fukuda et al., 1994[4]) was written for this purpose. The acronyms ME/CFS and CFS/ME are increasingly being used worldwide.

1:2 Epidemiology

The majority of patients present as sporadic or isolated cases, although cluster outbreaks of ME/CFS have occurred in many widely dispersed locations[5] including: Iceland (1948), London, England (1955), New Zealand (1984), and the USA (Nevada, 1984; New York State and North Carolina, 1985). The illness affects all ages, races and socioeconomic groups. Onset usually occurs between the ages of 30 and 50 years, but may occur at almost any age. It has been estimated that 0.42% of the adult U.S. population have ME/CFS and 70% of patients are female.[6] Higher and lower prevalence estimates have been published for several countries outside

the U.S. The prevalence in adolescents and children is uncertain, but appears to be lower than in adults, with equal numbers of boys and girls affected.

1:3 Diagnosis

With no validated diagnostic test for the illness, diagnosis is based on patient-reported symptoms as described in several overlapping case definitions. [1,4,7] This primer will use the 2003 Canadian Clinical Case definition,[1] which is intended for clinical practice and better targets the key symptoms of ME/CFS (See ME/CFS clinical diagnostic criteria worksheet page 12). Although considerable media attention has been given to ME/CFS, most patients with the illness have not been diagnosed. [6,8]

1:4 Presentation and Course of Illness

Illness onset may be characterized by flu-like symptoms that arise suddenly. Gradual onset may also occur. The illness can vary from mild to severe, with symptoms that may fluctuate significantly from hour to hour and day to day. A substantial number of patients with ME/CFS are bedridden, housebound, or wheelchair dependent. Many of these patients are too impaired to travel to office visits. Others, if not housebound, may be unable to hold a job. Those least affected may work part-time or even full time if their occupations are not too exhausting or if suitable accommodations are made. Some may need to find less demanding employment in order to continue working. Yet these higher functioning patients are often so exhausted from working that they spend many of their non-working hours resting.

The illness usually follows a relapsing and remitting course. Factors that can worsen the illness include: physical or mental overexertion, new infections, sleep deprivation, immunizations, distress from multiple sources (e.g., financial and marital problems, childcare demands, illness stigma) and co-existing medical conditions. In some cases, illness exacerbating factors cannot be identified. Improvements are not uncommon, but restoration of full pre-morbid health is rare in adults.[10] The level of functioning over sustained periods (e.g., at least six months) is a better indicator of worsening or improvement than a potentially temporary change seen during a single medical visit.

1:5 Role of the Health Practitioner in Diagnosis and Management

Patients who appear to have ME/CFS should be evaluated by a physician because: (1) the diagnosis depends on the exclusion of other fatiguing illnesses; (2) a proportion of patients with an initial diagnosis of ME/CFS are later found to have a different, treatable illness; and (3) treatable comorbid conditions may be present.

Establishing the diagnosis of ME/CFS will usually give the patient much relief. Early diagnosis with timely support and intervention (e.g., careful avoidance of over-exertion) is important as it may help to avoid deterioration and facilitate improvement. The chronicity of the illness indicates the need for ongoing management and periodic re-evaluation. Regular monitoring may reveal a change in the symptoms of ME/CFS or the emergence of a new, co-existing illness that may worsen fatigue and other CFS symptoms.

Given the complexities of this illness, a multidisciplinary team approach to management is desirable but rarely available. That said, patients can be successfully treated in a primary care setting, with appropriate referral to other health practitioners as needed. Clinical care focuses on improving symptoms and functioning by:

- Educating the patient about the illness (e.g., using handouts, see appendix F)
- Providing guidance on activity management and diet
- Treating symptoms with non-pharmacological and pharmacological interventions
- Monitoring progress with ongoing vigilance for the emergence of other illnesses

The health practitioner may also be asked to provide medical documentation for patients' disability insurance applications which, given their often limited financial resources, may be fundamental to their quality of life. The required documentation of patient impairments varies from country to country and from state to state in the USA.

2. ETIOLOGY OF ME/CFS

Over the past three decades, notable progress has been made in advancing our understanding of ME/CFS. Yet basic research on identifying causal factors remains an ongoing challenge given the heterogeneity of the illness and an evolving case definition. Both predisposing and precipitating factors are thought to contribute to the development of the illness.

2:1 Predisposing Factors

Female gender is a predisposing factor in adults. In some cases, susceptibility to ME/CFS may be inherited or familial. Family studies have shown that 20 percent of patients with sporadic ME/CFS have relatives who also have the illness, and 70 percent of such relatives were not living with the patient.[12] In addition, twin studies have found a CFS-like illness in 55% of monozygotic twins as compared to 19% in dizygotic twins.[13] A recent report found excess relative risk for developing ME/CFS in first (2.7), second (2.3) and third (1.3) degree relatives.[14]

2:2 Precipitating and Causal Factors

ME/CFS may be preceded by: an acute or a chronic infection (viral, bacterial or parasitic); exposure to environmental toxins (e.g. organophosphate pesticides); a recent vaccination; or a significant physical or emotional trauma.[16] These factors may affect immune function. However in some patients, no preceding illness or trauma can be identified. Factors that perpetuate the illness long-term are as yet unidentified.

A high percentage of patients date the onset of their ME/CFS to a flu-like illness. Over time, immune system changes similar to those seen in various chronic viral infections may be found. In some cases, ME/CFS follows infection with a known virus. For instance, one prospective study reported that six months after an initial primary infection with Epstein-Barr virus or Q fever, 11% of cases met the diagnostic criteria of ME/CFS. The severity of the initial infection in this study predicted a sustained illness.[17]

A number of viruses and/or the antibodies against them have been found more frequently in patients with ME/CFS than in control populations[15] (e.g., human herpes viruses, enteroviruses). These studies suggest that virus(es) may play a causative role. Alternatively, the viruses may be opportunistic infections. To date, no specific infectious agent has been uniquely linked to ME/CFS. Reports of the presence of the gammaretrovirus, XMRV, in patients with ME/CFS have been linked to an artifact of laboratory contamination.[11]

---

3. PATHOPHYSIOLOGY OF ME/CFS

The pathophysiological consequences of ME/CFS are multi-systemic and may include: immune and neuroendocrine abnormalities; brain dysfunction and neurocognitive defects; cardiovascular abnormalities; autonomic dysfunction; abnormalities in energy production including mitochondrial dysfunction; and changes in the expression of certain genes. Figure 1 presents one possible model of ME/CFS as a multi-system disorder. Although results from different research studies are sometimes contradictory, the evidence for abnormalities is more consistent in recent studies that assess the effects of exertional challenges utilizing physical (exercise or orthostatic) or cognitive (mental) tasks. Importantly, these provocation studies may be more likely to generate the core symptom of post-exertional malaise.[18-23, 58] Future research that recognizes the importance of exertion on illness variables may increase our understanding of this multifaceted condition.

3: 1 Immune System Abnormalities

The immune system abnormalities in patients with ME/CFS tend to wax and wane over time and may be associated with symptom severity. However, identified immune system abnormalities are not consistently found nor are they unique to the illness.

Figure 1.

Multisystem Dysregulation in ME/CFS

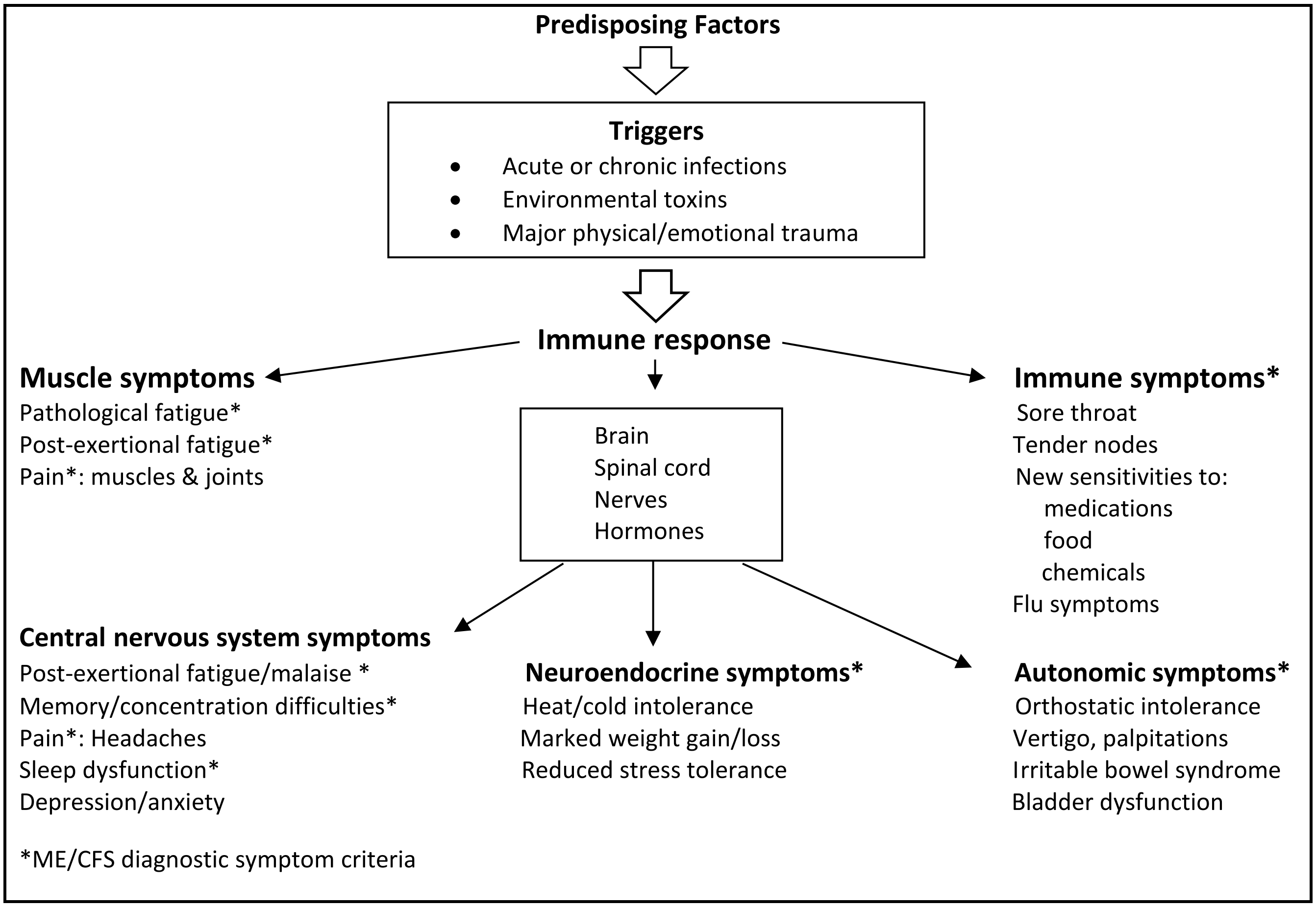

Immune system findings in patients with ME/CFS include:

- A shift towards a Th2 dominant immune response, with a preponderance of humoral over cell-mediated immunity [24]
- Immune activation with increased numbers of activated T lymphocytes, including cytotoxic T cells and elevated circulating cytokines[25]
- Poor cellular function with low natural killer cell cytotoxicity[26,120]
- A significant deficiency in plasma interleukin (IL) 15,[25] a cytokine necessary for NK cell activation and proliferation
- Dysregulation of the antiviral defense pathway 2-5A synthetase/RNase L, with an increase in low molecular weight 37kDa RNase L[27]
- The occasional finding of low levels of antinuclear antibodies, rheumatoid factor, thyroid antibodies and Lyme disease antibodies[28]

Fatigue and flu-like symptoms may be linked to elevated levels of various cytokines, including interferons and interleukins.[29] In addition lower cognitive function is significantly related to low NK cell function.[121] The dysregulation of the RNase L pathway supports the hypothesis that viral infection may play a role in the pathogenesis of the illness.

3:2 Neuroendocrine Dysregulation

One or more of the following neuroendocrine abnormalities has been found in studies of patients with ME/CFS:

- Mild hypocortisolism and attenuated diurnal variation of cortisol[30]
- Reduced function of the HPA axis, which can affect adrenal, gonad, and thyroid function[31]

- Blunted DHEA response to ACTH injection despite normal basal levels[32]
- Low IGF1 (somatomedin) levels and an exaggerated growth hormone response to pyridostigmine[33,34]
- Increased prolactin response to buspirone[35]
- A disturbance of fluid metabolism as evidenced by low baseline levels of arginine vasopressin[36]
- Relatively lower levels of aldosterone in patients compared with controls[37]
- Raised levels of neuropeptide Y (released in the brain and sympathetic nervous system following stress), possibly linked to the dysfunction of the HPA axis. Neuropeptide Y levels in plasma have been correlated with symptom severity [38]

3:3 Brain Abnormalities

Static and dynamic functional brain imaging techniques, EEG studies, and examination of the cerebrospinal fluid have revealed structural, functional, metabolic and behaviorally linked brain abnormalities in patients with ME/CFS. These abnormalities are not unique to the illness nor consistently found. However they can provide clues to illness pathophysiology. The findings include:

- Global reductions in gray matter[39] and punctuate areas of high signal intensity (white spots) in the white matter[40,41]
- Decreased brain perfusion and glucose metabolism[42,43]
- More areas of the brain recruited for processing incoming information as compared to controls[44]
- Slower cerebral activity in response to motor and visual imagery tasks than in controls[45]
- Increased ventricular lactate[46,47]
- Reduced slow wave sleep and prolonged sleep latency[48]
- Unique proteins found in cerebrospinal fluid[49]

3:4 Cognitive Impairment

Cognitive deficits are often the principal disabling feature of ME/CFS. Such deficits restrict the patient's ability to function, plan, and complete tasks in real world settings. Documented deficits include impaired working memory, slowed processing speed, poor learning of new information,[50,51] decreased concentration and attention span, difficulty with word retrieval, and increased distractibility[1,52]

Cognitive functioning may be disrupted by exercise,[53] over-sensitivity to noise and light, multiple stimuli and/or fast paced activity, and even routine social interactions. Standard neurocognitive testing batteries may not capture the cognitive difficulties experienced by patients in the real world. Individuals may be able to marshal their personal resources in the comparatively ideal conditions of the testing environment and the brief testing period. However, patients may be unable to sustain such efforts over prolonged periods where consistent performance (e.g., work, school) is required. Cognitive activity in itself can bring about diminished cognitive functioning as well as other post-exertional symptoms in a manner similar to that caused by physical exertion.

3:5 Autonomic Dysfunction/ Cardiovascular Abnormalities

Autonomic dysfunction can be seriously disabling. If present, it is manifested by an inability to maintain an upright posture or feeling faint or weak when standing (orthostatic intolerance) or sitting up. In such cases, tilt table testing may show neurally mediated hypotension (NMH) or postural orthostatic tachycardia syndrome (POTS).

Some patients with ME/CFS may complain of heart palpitations and show a persistent tachycardia at rest. Holter monitoring may reveal benign cardiac rhythm disturbances and non-specific T wave changes such as repetitive oscillating T-wave inversions and/or T-wave flattening.[54] Suspected diastolic dysfunction has been documented in some patients with ME/CFS using echocardiography. This diastolic dysfunction (improper ventricular filling) may be due to a lack of energy at the cellular level.[55] Low blood volume has also been found in some patients with ME/CFS.[56]

3:6 Mitochondrial/Energy Production Abnormalities

Recent studies suggest that mitochondrial dysfunction might be an important cause of the underlying energy deficit in patients with ME/CFS. One line of evidence indicates that aerobic energy production is impaired.[23, 57, 58] Because of this impairment, the

patient's exertions may exceed their anaerobic threshold. This results in a greater reliance on anaerobic metabolic pathways, which are far less efficient at producing energy. This process brings about the production of lactic acid and a disturbance of ATP/ADP metabolic cycling.[20, 57] However, the role of impaired aerobic metabolism in producing pathological fatigue, post-exertional malaise and a prolonged recovery time has not been fully elaborated.

Evidence for mitochondrial abnormalities includes: mitochondrial myopathy;[59] impaired oxygen consumption during exercise; activation of anaerobic metabolic pathways in the early stages of exercise;[19,20] and raised brain ventricular lactate levels.[46,47,60] With respect to exercise, a study of cardiopulmonary exercise testing, scheduled on two consecutive days showed an abnormal recovery response (decline in VO2max) on day two suggesting impaired metabolic function. By contrast, healthy control subjects were able to reproduce or slightly improve exercise performance over two consecutive days indicating that recovery from the initial exercise had occurred.[18,23]

3:7 Gene Studies

Gene studies in patients with ME/CFS suggest that the expression of certain genes may be altered.[22,61] These include altered expression of genes controlling immune modulation, oxidative stress and apoptosis. Several distinct genomic subtypes have been reported.[62] The presence of some of these subtypes has correlated with symptom severity.

In a recent controlled study,[22] two subgroups of patients with ME/CFS were identified with gene expression changes following exercise. The larger subgroup showed increases in mRNA for sensory and adrenergic receptors and a cytokine. The smaller subgroup contained most of the patients with orthostatic intolerance, and showed a post-exercise decrease in adrenergic α-2A receptor gene expression.

---

4. CLINICAL DIAGNOSIS

The diagnosis of ME/CFS is based on the patient's history, pattern of symptoms, and the exclusion of other fatiguing illnesses. A symptom-based diagnosis can be made with published criteria. This primer uses the 2003 Canadian clinical case definition for ME/CFS[1] (worksheet below), because of its emphasis on clearly described core symptoms of the illness. The 1994 Fukuda criteria for CFS[4] (Appendix A) are primarily used for research purposes, although they may be required for disability determinations in the US and elsewhere. The 2011 International Consensus Criteria for ME[7] are not yet in general use. No specific diagnostic laboratory test is currently available for ME/CFS, although potential biomarkers are under investigation.

The diagnostic criteria for the 2003 case definition are listed in the clinical worksheet on page 12 and can be copied and used for patient diagnosis. The second page of the worksheet includes diseases which must be excluded or fully treated before a diagnosis of ME/CFS can be established. A number of non-exclusionary co-morbid entities that commonly co-exist with ME/CFS are also listed. Patients with ME/CFS may have many symptoms in addition to those listed in the case definition.

4:1 Patient History

A thorough medical and social history is essential for accurate diagnosis. Obtaining a succinct and coherent history within one visit may not be possible given the cognitive difficulties in some patients. The information gathered should include pre-illness functioning (job/school performance, social and family relationships) and current living circumstances (daily activities, stressors, major life changes, and support sources). Assessment of functioning will reveal the significant life changes experienced by the patient as a result of the illness. A review of previous medical records, reports, and lab tests supplied by the patient may also provide useful information.

ME/CFS: A Primer for Clinical Practitioners

ME/CFS Clinical Diagnostic Criteria Worksheet[*]

Name __________________________________ Patient ID___________________

To diagnose ME/CFS, the patient must have the following:

- **Pathological fatigue, post-exertional malaise, sleep problems, pain, two neurocognitive symptoms, and at least one symptom from two of the following categories: autonomic, neuroendocrine, immune**
- **The fatigue and the other symptoms must persist, or be relapsing for at least six months in adults, or three months in children and adolescents. A provisional diagnosis may be possible earlier**
- **The symptoms cannot be explained by another illness.**

Improved diagnostic accuracy can be obtained by measuring the severity and frequency of the listed symptoms.†

Symptoms	Description of Symptoms
**Pathological fatigue**<br>**Yes [ ] No [ ]**	**A significant degree of new onset, unexplained, persistent or recurrent physical and/or mental fatigue that substantially reduces activity levels and which is not the result of ongoing exertion and is not relieved by rest**
**Post-exertional malaise & worsening of symptoms**<br>**Yes [ ] No [ ]**	**Mild exertion or even normal activity is followed by malaise, the loss of physical and mental stamina and/or worsening of other symptoms. Recovery is delayed, taking more than 24 hours**
**Sleep problems**<br>**Yes [ ] No [ ]**	**Sleep is un-refreshing:**<br>**disturbed quantity - daytime hypersomnia or nighttime insomnia**<br>**and/or disturbed rhythm - day/night reversal**<br>Rarely, there is no sleep problem
**Pain**<br>**Yes [ ] No [ ]**	**Pain is widespread, migratory or localized:**<br>**myalgia; arthralgia (without signs of inflammation); and/or**<br>**headache - a new type, pattern or severity**<br>Rarely, there is no pain
**Two Neurocognitive symptoms**<br>**Yes [ ] No [ ]**	**Impaired concentration, short term memory or word retrieval;**<br>**hypersensitivity to light, noise or emotional overload;**<br>**confusion; disorientation; slowness of thought; muscle weakness; ataxia**
**At least one symptom from two of these categories:**<br>**(a) Autonomic**<br>**Yes [ ] No [ ]**	**(a) Autonomic:**<br>**Orthostatic intolerance; neurally mediated hypotension (NMH);**<br>**postural orthostatic tachycardia (POTS); light-headedness;**<br>**extreme pallor; palpitations; exertional dyspnea;**<br>**urinary frequency; irritable bowel syndrome (IBS); nausea**
**(b) Neuroendocrine**<br>**Yes [ ] No [ ]**	**(b) Neuroendocrine:**<br>**Low body temperature; cold extremities; sweating;**<br>**intolerance to heat or cold; reduced tolerance for stress; other symptoms worsen with stress; weight change; abnormal appetite**
**(c) Immune**<br>**Yes [ ] No [ ]**	**(c) Immune:**<br>**Recurrent flu-like symptoms; sore throats; tender lymph nodes;**<br>**fevers; new sensitivities to food, medicines, odors or chemicals**

ME/CFS Clinical Diagnostic Criteria Worksheet (continued)

Symptom Characteristics:

- A sudden onset is most common, but the onset may be gradual
- Symptoms may vary from day to day or during the day
- Relapses and remissions are frequent
- Post-exertional symptom flare-ups may occur immediately or they can be delayed 24 hours or more
- If pain and/or sleep disorder are absent, ME/CFS can be diagnosed if the illness has an abrupt onset

Exclusionary illnesses:

Many other illnesses have symptoms that mimic ME/CFS symptoms. Active disease processes that could explain the major symptoms of fatigue, sleep disturbance, pain, and neurocognitive dysfunction must be ruled out by history, physical examination and medical testing. The following lists some more common, exclusionary conditions:

- **Anemias**
- **Autoimmune diseases** such as rheumatoid arthritis, lupus
- **Cardiac disease**
- **Endocrine disorders** such as diabetes, Addison's disease, thyroid disease, menopause
- **Infectious diseases** such as tuberculosis, HIV/AIDS, chronic hepatitis, Lyme disease
- **Intestinal diseases** such as celiac or Crohn's disease
- **Malignancies**
- **Neurological disorders** such as multiple sclerosis, Parkinson's disease, myasthenia gravis
- **Primary psychiatric disorders** and substance abuse (but not clinical depression)
- **Significant pulmonary disease**
- **Primary sleep disorders** such as sleep apnea.

Non-exclusionary conditions:

- **Some co-morbid entities commonly occur in association with ME/CFS. They include:** a**llergies, fibromyalgia (FM), irritable bowel syndrome (IBS) and multiple chemical sensitivities (MCS)**
- **Any medical condition that has been adequately treated and is under control**
- **Any isolated physical abnormality or laboratory test that is insufficient to diagnose an exclusionary condition.**

ME/CFS and FM are often closely associated and should be considered to be overlapping syndromes.
A co-morbid condition may precede the onset of ME/CFS by many years, but then become associated with it.

If the patient has unexplained, prolonged fatigue but has an **insufficient number of symptoms to meet the criteria for ME/CFS,** the illness should be classified as i**diopathic chronic fatigue**.

_________ Patient meets the criteria for ME/CFS

_________ Full criteria not met but patient should be monitored

Comments:

____________________________________ ________________

Provider's Signature **Date**

* Carruthers BM, et al. ME/CFS: Clinical Working Case Definition, Diagnostic and Treatment Protocols. *J CFS* 2003; 11(1):7-115.
†Jason LA, et al. The development of a revised Canadian Myalgic Encephalomyelitis-Chronic Fatigue Syndrome case definition. *American J Biochemistry Biotechnology* 2010; 6(2): 120-135.

4:2 Physical Examination

Physical findings are often subtle and may not be obvious. Patients may look pale and puffy with suborbital dark shadows or shiners. Examination of the patient's pharynx may show non-exudative pharyngitis (often referred to as "crimson crescents"). Cervical and axillary lymph nodes may be palpable and tender.

Some patients have demonstrable orthostatic intolerance with neurally mediated hypotension or postural orthostatic tachycardia syndrome, characterized by lowered blood pressure and/or a tachycardia on prolonged standing. This may be associated with dependent rubor in the feet and pallor of the hands.

A neurological examination may reveal a positive Romberg test or positive tandem stance test. If widespread pain is reported, a concurrent diagnosis of fibromyalgia should be considered and confirmed with a tender point examination.

4:3 Laboratory Tests

A basic laboratory investigation **(Table 1)** should be followed with more specific tests **(Table 2)** depending on particular symptoms. For example, an EKG/ECG should be performed if chest pain is present, a chest x-ray obtained for cough, and testing for celiac disease if gastrointestinal symptoms are reported. (An endoscopy is recommended if symptoms are severe).

Results of routine tests in patients with ME/CFS are usually within the normal range even during severe relapses. If abnormalities are found (e.g., elevated erythrocyte sedimentation rate [ESR]), other diagnoses may be considered.

Table 1
Investigation of ME/CFS: Routine Laboratory Testing

• Full blood count and differential<br>• Erythrocyte sedimentation rate<br>• Electrolytes: sodium, potassium, chloride, bicarbonate<br>• Calcium<br>• Phosphate<br>• Fasting glucose<br>• C-reactive protein<br>• Liver function: bilirubin, alkaline phosphatase (ALP), gamma glutamyl transaminase (GGT), alanine transaminase (ALT), aspartate transaminase (AST)	• Albumin/globulin ratio<br>• Renal function: urea, creatinine, glomerular filtration rate (eGFR)<br>• Thyroid function: thyroid stimulating hormone (TSH), free thyroxine (free T4)<br>• Iron studies: serum iron, iron-binding capacity, ferritin<br>• Vitamin B12 and serum folate<br>• Creatine kinase (CK)<br>• 25-hydroxy-cholecalciferol (Vitamin D)<br>• Urinalysis

Specific tests from Table 2 may show low morning cortisol, elevated antinuclear antibody (ANA), and/or immunoglobulin abnormalities. In addition, Vitamin D levels are often low,[63] which would suggest bone density testing for osteoporosis. Any abnormal finding warrants further investigation to exclude other diseases.

Research studies have reported a number of immune, neuroendocrine and brain abnormalities in patients with ME/CFS, but the clinical value of expensive and elaborate tests for these abnormalities has not been established.

4:4 Differential Diagnosis (Table 3)

Although the symptoms of a number of diseases can mimic ME/CFS, the presence of post-exertional malaise and symptom exacerbation, a key feature of the illness, increases the likelihood of ME/CFS as the correct diagnosis. **Table 3** lists a number of medical conditions that need to be considered in the differential diagnosis.

Table 2

Investigation of ME/CFS: Tests to be Considered Depending on Symptoms

- Cardiac: chest x-ray, electrocardiogram (EKG/ECG), tilt table test for autonomic function
- Endocrine/Metabolic: morning cortisol, short ACTH challenge test or cortisol stimulation test, prolactin, parathormone, rennin/aldosterone ratio, serum amylase, estradiol, follicle-stimulating hormone, testosterone
- Gastrointestinal: endoscopy: gastroscopy, colonoscopy; gastric emptying study, gliadin and endomysial antibodies
- Infectious Diseases: screen for HIV, hepatitis, Lyme disease, Q fever and microbiology of stools, throat, urine, sputum, genital as appropriate
- Immunology/Autoimmunity: antinuclear antibodies, rheumatoid factors, total and subclass immunoglobulins, functional antibodies and lymphocyte subsets
- Neurological: MRI if multiple sclerosis suspected
- Pulmonary: overnight polysomnogram and possibly multiple sleep latency test
- Urinary: cystoscopy

Table 3

Differential Diagnoses

**AUTOIMMUNE/RHEUMATOLOGY**<br>Polymyalgia rheumatica<br>Rheumatoid arthritis<br>Systemic lupus erythematosis	**HEMATOLOGICAL**<br>Anemias<br>Hemochromatosis<br>Leukemia or lymphoma<br>Myelodysplastic syndromes	**PSYCHIATRIC**<br>Bipolar disorder<br>Generalized anxiety disorder<br>Major depressive disorder<br>Post-traumatic stress disorder<br>Personality disorder
**CARDIOVASCULAR**<br>Cardiomyopathy<br>Claudication<br>Coronary artery disease<br>Heart valve disease<br>Patent foramen ovale<br>Pulmonary hypertension	**INFECTIONS**<br>Acute mononucleosis<br>Bornholm disease (Coxsackie)<br>Brucellosis<br>Giardia<br>Hepatitis B or C<br>HIV<br>Leptospirosis<br>Lyme disease<br>Parvovirus<br>Post-polio syndrome<br>Q fever<br>Toxoplasmosis<br>Tuberculosis	**RESPIRATORY**<br>Aspergillosis<br>Asthma or allergies<br>Sarcoidosis
**ENDOCRINE/METABOLIC**<br>Addison's disease<br>Hyper- and hypothyroidism<br>Hyper- and hypocalcemia<br>Male hypogonadism<br>Menopause<br>Metabolic syndrome<br>Pituitary tumors or disorders<br>Vitamin B12 or D deficiency	**NEUROMUSCULAR**<br>Multiple Sclerosis<br>Myasthenia gravis<br>Myopathies and neuropathies<br>Parkinson's disease	**SLEEP DISORDERS**<br>Central sleep apnea<br>Obstructive sleep apnea<br>Narcolepsy<br>Periodic leg movements
**GASTROINTESTINAL**<br>Celiac disease<br>Food allergy or intolerances<br>Inflammatory bowel diseases		**TOXIC SUBSTANCES**<br>Alcohol or drug abuse<br>Ciguatera poisoning<br>Lead, mercury or other heavy metal poisoning<br>Organophosphate pesticide poisoning<br>Reactions to prescribed drugs
**MALIGNANCY**<br>Primary and secondary cancers		**OTHER CONDITIONS**<br>Chiari 1 malformation<br>Gulf war syndrome

4:5 Exclusionary Medical Conditions

ME/CFS is not diagnosed if the patient has an identifiable medical or psychiatric condition that could plausibly account for the presenting symptoms. However, if ME/CFS symptoms persist after adequate treatment of the exclusionary illness, then a diagnosis of ME/CFS can subsequently be made.

4:6 Co-existing Medical Conditions (Table 4)

A number of (non-exclusionary) conditions may co-exist with ME/CFS. These conditions are listed in **Table 4** and include fibromyalgia, multiple chemical sensitivity, orthostatic intolerance, irritable bowel syndrome, irritable bladder syndrome, interstitial cystitis, sicca syndrome, temporo-mandibular joint syndrome, migraine headache, allergies, thyroiditis, Raynaud's phenomenon and prolapsed mitral valve. These conditions should be investigated in their own right and treated appropriately.

Table 4
Non-exclusionary Overlapping Conditions

**AUTOIMMUNE**<br>Sicca syndrome<br>Sjogren's syndrome<br>**CARDIOVASCULAR**<br>Autonomic dysfunction<br>• orthostatic intolerance<br>• neurally mediated hypotension (NMH)<br>• postural orthostatic tachycardia syndrome (POTS)<br>• syncope<br>Mitral valve prolapse<br>**DERMATOLOGICAL**<br>Acne rosacea<br>**ENDOCRINE/METABOLIC**<br>HPA axis dysregulation<br>• low normal cortisol<br>• hypogonadism<br>• premature menopause<br>Hypoglycemia<br>Insulin resistance<br>Metabolic syndrome<br>Multiple chemical sensitivities<br>Obesity (BMI< 35)	**GASTROINTESTINAL**<br>Food allergy and intolerances<br>• lactose<br>• gluten, celiac or sprue-like disorders<br>• milk protein<br>Gut motility disorder<br>• reflux, dysphagia, early satiety<br>• irritable bowel syndrome<br>**GYNECOLOGICAL**<br>Abdomino-pelvic pain<br>Endometriosis<br>Premenstrual syndrome<br>Premenstrual dysphoric disorder<br>Vulvodynia<br>Vulvar vestibulitis<br>**HEMATOLOGICAL**<br>Bruisability<br>**NEUROLOGICAL**<br>Hypersensitivities<br>light, sound, touch,<br>odors or chemicals<br>Visual midline shift syndrome (symptoms)<br>• dizziness/nausea<br>• poor balance	**RESPIRATORY**<br>Allergies<br>Bronchoconstriction<br>reactive airways or asthma<br>Rhinitis<br>• allergic<br>• vasomotor<br>• infectious<br>**RHEUMATOLOGICAL**<br>Costochondritis<br>Fibromyalgia<br>Myofascial pain syndrome<br>Ehlers-Danlos syndrome<br>• joint hyperlaxity<br>• hyperelasticity<br>Sacroiliac joint tenderness<br>Temporomandibular joint dysfunction (TMD)<br>**SLEEP DISORDERS**<br>Periodic limb movement disorder (PLMD)<br>Non-restorative sleep<br>Restless legs syndrome<br>**URINARY**<br>Interstitial cystitis<br>Overactive bladder<br>Prostatitis

4:7 Differentiating between Depressive/Anxiety Disorders and Psychological Reactions Secondary to ME/CFS

Similar to other chronic illnesses, emotional reactions in response to the realities of living with ME/CFS are common. Reactive emotions may include grief and anger over illness-related losses, frustration at the inability to do things, apprehension about negative changes such as potential job loss and generalized discouragement. These adjustment reactions do not ordinarily, rise to the level of diagnosable psychiatric disorder; but in some patients, clinically diagnosable major depressive disorder (MDD) or anxiety may co-exist with ME/CFS. Sometimes MDD and anxiety may precede the illness.

It is important to distinguish secondary psychological reactions to ME/CFS from MDD or anxiety dis-

order, without co-existing ME/CFS and also from MDD or anxiety disorder co-existing with ME/CFS. This may present a challenge[64,65] as many symptoms, including fatigue, change in activity levels, unrefreshing sleep, poor memory and concentration, and appetite/weight changes occur in both ME/CFS and in depressive and anxiety disorders.

Nevertheless, relatively distinct distinguishing features of ME/CFS, (listed in the case definition) can be identified. In particular, post exertional malaise is a characteristic symptom of ME/CFS that is not seen in primary MDD. In addition, the occurrence of recurrent flu-like symptoms, sore throats, tender lymph nodes, orthostatic intolerance and hypersensitivities to light, noise and medications may be helpful in distinguishing ME/CFS from primary MDD.

Symptoms typically seen in patients with MDD include very low mood, suicidal thoughts, a pervasive loss of interest, motivation and/or enjoyment (anhedonia) and a sense of worthlessness or guilt. These are not seen in unmixed ME/CFS.

Other distinguishing characteristics are as follows: (1) patients with major depression often feel better after increased activity, exercise or focused mental effort, whereas patients with ME/CFS often experience post-exertional malaise (PEM). For example a short walk may trigger a long-lasting symptom flare-up. (2) diurnal fluctuations in ME/CFS tend to show symptom-worsening in the afternoon whereas in major depressive disorder more severe symptoms often occur in the morning. Research studies have shown that there are biochemical differences between ME/CFS and MDD.[114,115,123,]

In some patients with ME/CFS, major depressive disorder develops and co-exists with the illness and suicide is a risk. If the depression features suicidal thoughts, the clinician should perform a suicide risk evaluation and consider referral for psychiatric evaluation.

Finally, patients with ME/CFS (with or without co-morbid depression) generally have a strong desire to be more active, but often cannot; while patients with major depression could be more active, but do not have the desire to do so.

Anxiety secondary to ME/CFS can arise with the crisis of illness onset and persist as the illness affects the ability to work and family relationships. Secondary anxiety may be distinguished from generalized anxiety disorder (GAD). GAD is characterized by excessive worry and assorted physical symptoms and panic disorder features unbidden panic attacks. By comparison, symptoms of ME/CFS not found in GAD and panic disorders include post-exertional malaise as well as autonomic, endocrine and/or immune symptoms (see Diagnostic Worksheet). In addition, patients with primary anxiety disorders generally feel better after exercise, whereas exercise worsens symptoms in patients with ME/CFS. Finally panic disorder is situational and each episode is short-lived, whereas ME/CFS persists for years.

---

5. MANAGEMENT/TREATMENT

The onset of ME/CFS impacts the individual's ability to work, to sustain family and social relationships, to provide basic self-care, and to maintain self-identity. These sudden losses may trigger confusion and crisis. Yet patients often receive little benefit from consultations with health practitioners due to (1) physician skepticism of individuals with ME/CFS who may not look ill and show normal findings on standard physical examinations and laboratory tests; and (2) the absence of a clear standard of care for these patients. These obstacles, in addition to significant illness limitations and unsupportive family and friends, may lead to patients feeling demoralized, frustrated and angry.

This chapter provides recommendations primarily for ambulatory patients who are able to attend office visits. Special considerations are offered in chapter 7 for the substantial number of patients with ME/CFS who are bedridden, house-bound, or wheelchair dependent.

5.1 Approach to Treatment

Given the absence of curative treatments, clinical care of ME/CFS involves treating symptoms and guiding patient self-management. The goal is symptom reduction and quality of life improvement based on a collaborative therapeutic relationship. Although not all patients will improve, the potential for improvement, which ranges from modest to substantial, should be clearly communicated to the patient.

Acknowledging that the patient's illness is real will facilitate a therapeutic alliance and the development of an effective management plan. Thus, patients may be greatly relieved to hear that their bewildering symptoms have a diagnostic label – an important validation of their concerns. The practitioner can also assure the patient that normal findings on diagnostic tests do not negate the reality of the illness.

Once the diagnosis is established, a systems review will reveal the patient's most troublesome symptoms and concerns. These may include several of the following: debilitating fatigue and activity limitations; sleep disturbance; pain; cognitive problems; emotional distress; orthostatic intolerance; gastro-intestinal or urological symptoms; gynecological problems.

The clinical management plan in this section focuses on both non-pharmacologic interventions and medications. Because many patients have short-term memory problems, written educational material (e.g., appendix F) can also be helpful. Many local, state and national ME/CFS patient organizations also distribute educational material.

To improve clinical management, we suggest the following:

- Recommend to patients that they bring a support person (family member or friend) to take down medical advice or make a recording of the visit for later patient review
- Obtaining a written list of the patient's most troublesome symptoms
- Agreement with the patient to focus on a limited number of selected symptom(s) in order to avoid overloading the patient.
- Medication doses that start low and go slow
- Ongoing assessments of the patient over multiple visits

The order of ME/CFS symptoms presented below starts with those considered most treatable.

5:2 Sleep

Patients with non-restorative sleep wake up feeling unrefreshed or feeling as tired as the night before. The unrefreshed feeling may be associated with morning stiffness or soreness and mental fogginess that may last for an hour or two. Disturbed sleep patterns include difficulty falling or staying asleep, frequent awakenings, or coma-like sleep. Hypersomnia may occur in the early stages of the illness. Many patients have a diagnosable sleep disorder that may require consultation with a sleep disorder specialist.

The following sleep hygiene suggestions may be helpful to patients: [66]

- An hour of relaxing wind-down activities prior to bed time
- Regular sleep and wake times
- Pacing activities during the day to avoid symptom exacerbation that may interfere with sleep
- Avoiding naps after 3 pm and substituting rest
- Spending some morning time under full spectrum light either outdoors, by a window, or artificial light[67]
- Reducing or eliminating caffeine-containing beverages and food
- Using earplugs or soundproofing for noise, or sleeping in a different bedroom without (a snoring) partner
- Ensuring the bedroom is very dark by using a sleep mask or black-out curtains
- If unable to sleep, getting up and moving to another room, and doing a quiet activity (reading, soft music, or relaxation tapes; not a computer, iPad, or TV) until sleepy
- Avoiding attempts to force sleep
- A carbohydrate snack at bedtime may be helpful[124]

Medications (Table 5). All sedating medications must be safe for long-term use and should be started at a low dose. The medication should be taken early enough so that sedation takes effect

around bed time. Patients may initially feel thick-headed in the morning, but this usually improves as benefits become apparent. The risk of side effects and drug combinations which can produce serotonin syndrome should be explained. In some patients, tolerance may develop with medications. Rotating medications may be more effective than using a single drug.

Table 5
Medications for Sleep

Medication	Dose	Comments
Tricyclics: Amitriptyline, Doxepin, Nortriptyline	5-100 mg	Take 1-2 hours before bedtime. May worsen dry mouth, constipation, orthostatic intolerance, or cause daytime sedation
Cyclobenzaprine	5-10 mg	Same comments as tricyclics above
Trazodone	12.5-200 mg	May be the least likely to lose effectiveness for sleep
Quetiapine	12.5-100 mg	May cause weight gain or extrapyramidal symptoms
Gabapentin	100-1500 mg	May help nocturnal pain and restless legs syndrome
Pregabalin	50-450 mg	Helpful for nocturnal pain, but very sedating for some
Antihistamines:<br>Promethazine<br>Diphenhydramine	 10 mg<br>50 mg	Anticholinergic side effects
Clonazepam	0.25-1 mg	For restless legs, muscle spasms or anxiety.
Orphenadrine	100 mg	For restless legs or muscle spasms (not available everywhere)
Ropinirole or Pramipexole	0.125-0.25 mg	For restless legs or muscle spasms (not available everywhere)
Melatonin	1-3 mg or more, 2-3 hours before bedtime	May help patients who have altered circadian rhythms
Zolpidem	2.5-10 mg	Short duration of action may lead to rebound insomnia
Zopiclone	7.5 mg	Short duration of action may lead to rebound insomnia
Mirtazapine	7.5-15 mg	May cause daytime sedation; tolerance may develop

5:3 Pain

Persistent pain in ME/CFS, whether widespread or localized, may range from mild to severe. In some cases the patient may feel pain from minimal stimulation such as a gentle touch. Headaches may be particularly troublesome and are often migrainous. If widespread pain is a major complaint, a fibromyalgia evaluation may be indicated.

Helpful non-pharmacologic interventions may include pacing of activity, physical therapy, stretches, massage, acupuncture, hydrotherapy, chiropractic, yoga, Tai Chi and meditation (relaxation response).[68,69] Also consider hot or cold packs, warm baths, muscle liniments, electrical massagers, TENS (transcutaneous electrical nerve stimulation), and rTMS (transcranial magnetic stimulation). These approaches can be effective singly or in various combinations. However, they may also be poorly tolerated, inaccessible, or prohibitively costly. It is important to treat localized pain, e.g., arthritis or migraine, because it can amplify the generalized pain of ME/CFS.

Medications (Table 6). For the treatment of pain in ME/CFS, the lowest effective dose should be prescribed and increased cautiously. Patients with severe pain may need the stronger analgesics and narcotics. Although opiates are discouraged for the treatment of chronic pain states, they may be beneficial in some cases. Their use requires a clear rationale with documentation. Providers should consider referring such patients to a pain specialist.

Table 6
Medications for Pain

Medication	Dose	Comments
Acetaminophen<br>Paracetamol	500-1000 mg prn 8 hrly	Often ineffective
Aspirin	300-600 mg prn 6-8 hrly	Often ineffective
NSAIDS:<br>Diclofenac<br>Naproxen	 75-100 mg daily<br>500-1000 mg daily	Often ineffective. May exacerbate gastritis or reduce renal function
Tricyclics		Helpful for most chronic pain, see sleep section
SNRIs:<br>Duloxetine<br>Milnacipran	 20-90 mg daily<br>25-100 mg bid	May increase sweating, blood pressure or heart rate
Narcotics:<br>Codeine phosphate<br>Opiates such as oxycodone, hydrocodone; morphine	 doses vary, consult guidelines	Constipation/habituation<br>Narcotics should be avoided if possible
Tramadol	50-100 mg, qd 6-8 hrly	Seizure risk and interaction with drugs that raise serotonin

5:4 Fatigue and Post-exertional Malaise

Patients with ME/CFS experience abnormal fatigue that is both more intense and qualitatively different from normal tiredness. The fatigue in ME/CFS may take several different forms:[70] post-exertional fatigue (abnormal exhaustion or muscle weakness following minor physical or cognitive activity), persistent flu-like feelings, brain fog (mental exhaustion from everyday cognitive effort), and wired fatigue (feeling over-stimulated when very tired).

The type of fatigue that is a core feature of ME/CFS is post-exertional malaise (PEM). PEM is the exacerbation of fatigue and other symptoms (e.g., cognitive difficulties, sore throat, insomnia) following minimal physical or mental activity that can persist for hours, days or even weeks. PEM may be related to abnormal energy metabolism.

Energy for physical activities is produced through two physiological systems: (1) Anaerobic metabolism is the predominant metabolic pathway during the first 90 seconds of exercise; (2) The aerobic/oxidative system is the primary source of energy during physical activities lasting longer than 90 seconds. Because most daily physical activities exceed 90 seconds, the aerobic system is typically utilized to produce the energy-releasing nucleotide adenosine triphosphate (ATP), at a steady rate in order to perform activities of daily living. In patients with ME/CFS, aerobic metabolism may be impaired.[23,57,58] Thus, any physical exertion exceeding 90 seconds may utilize a dysfunctional aerobic system, which leads to increased reliance on anaerobic metabolism. This imbalance may be linked to the prolonged symptoms and functional deficits associated with PEM.

Activity and exercise

Activities of daily living utilize energy and are in themselves, exercise. These activities may constitute an optimum amount of exercise for some patients or an excessive amount of exercise for others while for some patients, adding a suitable exercise program (exercise therapy) can improve function and quality of life. However, exercise has not been shown to produce a long term cure for ME/CFS and studies have not shown that exercise reverses the pathophysiological changes found in this illness. Care must be taken to ensure that an exercise program does not exceed the patient's energy envelope, because for many patients, adding an injudicious exercise program has caused post exertional malaise and deterioration of func-

tion. Any exercise program should not take priority over activities of daily living.

Managing post-exertional symptoms: Pacing and the energy envelope

Fatigue improvement can be facilitated by advising patients to pace or "spread out" activities so that ongoing exertion remains below the threshold of post-exertional symptom flare-ups[71] (Figure 2). For instance, rather than completing housework in one uninterrupted push, tasks may be divided into smaller pieces with rest intervals interspersed. Remaining as active as possible while avoiding fatigue-worsening over-exertion delineates an optimal zone of activity termed the "energy envelope."

An activity log[118] (Appendix D) may be helpful to identify personal activities that stay within or exceed that optimal range.

Figure 2

Fatigue Severity Declines When Patients Stay Within Energy Envelope[71]

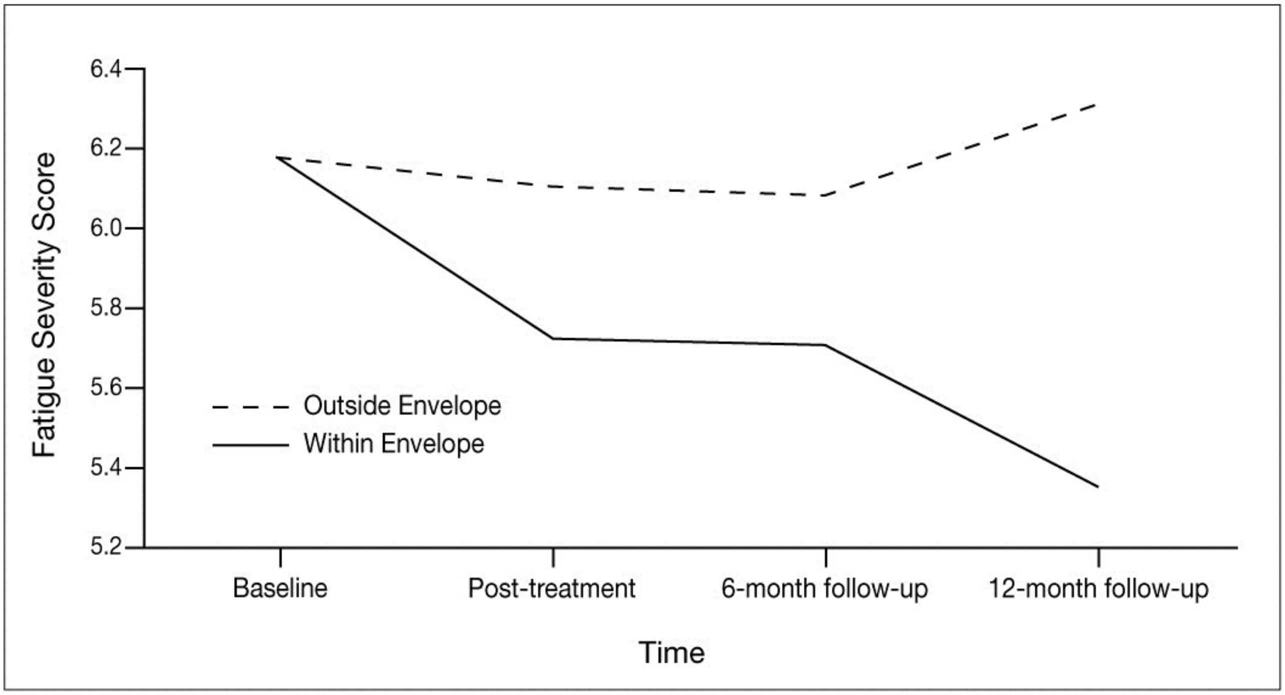

Pacing

To stay within the energy envelope, some patients need to decrease their activity while others need to carefully and selectively do more. Many individuals with ME/CFS mistakenly over-exercise in an attempt to reduce fatigue and other symptoms. In addition, well-meaning healthcare providers may recommend exercise for patients with ME/CFS using guidelines intended for healthy people. Such guidelines are generally inappropriate and often counterproductive in this illness. Thus, practitioners may push patients too hard and patients may push themselves into activities that worsen symptoms, and this discourages further exercise. Symptom-worsening after exercise may result from underlying aerobic impairment.[23,57,58] The optimal amount of individualized exercise is usually well below standard recommendations for healthy individuals, avoids post-exertional symptoms, and promotes improvement.

Activity recommendations. An individualized activity plan should be developed in collaboration with the patient.[72,73] Consultation with rehabilitation professionals knowledgeable about ME/CFS may also be desirable. Any exercise or activity program should seek to minimize the negative effects of exertion on impaired aerobic function.

Initially, the patient's degree of activity limitation can be estimated using a functional status rating

such as the Functional Capacity Scale (Appendix C). This 10 point scale ranges from 10, for symptom free individuals, to 0, for patients who are bedridden and unable to perform activities of daily living.

Severely ill patients, functional capacity rating 0-3 (Appendix C). See section 6:1.

Less severely ill patients. (functional capacity rating 3-5 (Appendix C). Exercise lying down should be advised when exercise standing or sitting is poorly tolerated. Initially, interval training exercise should begin with gentle stretching to improve mobility utilizing intervals of 90 seconds or less. The patient should rest between intervals until complete recovery has occurred. Additional intervals can be added when the stretching exercises do not trigger post-exertional symptoms. Then, resistance training can begin with elastic bands or light weights. If endurance improves, short-duration interval training such as leisurely-paced walking can be added.

Higher functioning patients, functional capacity rating 5-9 Appendix C). Interval training can begin with leisurely paced walking, swimming, or pedalling on an exercise cycle.[74] The initial duration may vary from 5-15 minutes a day depending on how much the patient can do without provoking symptom flares. These higher functioning patients may also benefit from adaptive yoga and Tai Chi.

Progressive improvement is not always obtained. Many patients reach a plateau, after which no further improvement occurs. Fluctuations in illness severity are also common and may require that patients modify their activities for a period of time.

Medications for fatigue and post-exertional symptoms (Table 7). Due to prescribing difficulties, cost, and limited effectiveness, medications for fatigue may need to be reserved for functional assistance at special, but potentially exhausting events in the patient's life (e.g., a wedding or a concert). If the medication is effective, careful monitoring of activity is recommended, patients should avoid exceeding their individual activity limit, as this can provoke symptom-worsening.

Table 7
Medications for Fatigue

Medication	Dose	Comments
Modafinil	100-200 mg qd	Unsuccessful in formal studies
Armodafinil	150-250 mg qd	
Methylphenidate	5 -20 mg tid	Moderate to marked benefit anecdotally but tolerance develops if used daily; may be habituating
Dexamphetamine	5-10 mg tid	Somewhat successful in a small trial; may be habituating. Tolerance may develop if used daily; may affect BP and HR
Caffeine		Patients often self-medicate with caffeine-containing products (e.g., drinks, supplements, tablets); may disturb sleep if taken late in the day

5:5 Cognitive Problems

The patient's cognitive difficulties can be managed to some extent with the following suggestions:

- Using a "memory book" to write things down in one place (and attempt not to lose the book)
- Developing habits such as leaving keys or glasses or always parking in the same spot
- When possible, avoiding situations involving multisensory bombardment and fast-paced activity
- Limiting the duration and intensity of cognitive efforts (a form of pacing (see section 5:4)
- Limiting or stopping cognitive efforts when cognitive symptoms flare up.

Medications for cognitive problems (Table 8) Stimulants seem to work best when the patient describes excessive daytime "sleepiness" as opposed to "tiredness." Sleepiness is suggested by a score of >10 on the Epworth sleepiness scale. This may warrant a workup for primary sleep disorders.

Table 8
Medications for Cognitive Problems

Medication	Dose	Comments
Methylphenidate	5-20 mg tid	May be habituating
Dexamphetamine	5-10 mg tid	May affect BP and HR; may be habituating
Amphetamine salts	5-20 mg tid	May affect BP and HR; may be habituating
Modafinil	100-200 mg qd	Start with a small dose and increase slowly to the most effective dose
Armodafanil	150-250 mg qd	Start with a small dose and increase slowly to the most effective dose
Caffeine		Patients often self-medicate with caffeine containing products, may disturb sleep if taken late in the day

5:6 Managing Depression, Anxiety and Distress with Education, Support and Coping Skills

To differentiate between symptoms of depression and anxiety secondary to ME/CFS and major depressive and anxiety disorders see section 4:7. If depression takes on the appearance of a major depressive disorder (MDD) and if suicidal thoughts are present, referral may be necessary.

Helpful interventions include:

- Educating the patient and family members about the illness, so the patient can understand what is happening to him/herself and so family members can provide useful assistance and support to the patient
- Helping the patient and family find useful and pleasurable low-effort activities (music, recorded relaxation, observing nature). Such activities may assist the patient in developing a sense that life can be rewarding and may also reduce symptoms of anxiety, depression and distress and lessen fatigue.[75,76]
- Developing coping skills such as strategies to reduce anger, worry, and catastrophizing and skills to improve tolerance of this difficult illness. Good resources are available to guide patients toward the development of effective coping skills.[64,77]
- Referral, if needed, to supportive counseling, preferably to a professional familiar with ME/CFS
- Referral to a ME/CFS support group or volunteer services. Successful support groups have effective leadership and positive programming that avoids simply exchanging complaints

Medications for depression. For patients who are clinically depressed, medication can sometimes improve mood and reduce fatigue. Medications should be started at a low dose. Improvement may take several weeks. Possible side effects of antidepressants, notably sedation and orthostatic hypotension, may worsen fatigue and autonomic lability occurs in some patients. Drug choice is often based on side effects profile and the patient's response.

5:7 Cognitive Behavioral Therapy (CBT)

CBT is a much publicized and debated psychotherapeutic intervention for ME/CFS that addresses the interactions between thinking, feeling and behavior. It focuses on current problems and follows a structured style of intervention that usually includes a graded activity program. CBT may improve coping strategies and/or assist in rehabilitation, but the premise that cognitive therapy (e.g., changing "illness beliefs") and graded activity can "reverse" or cure the illness is not supported by post-intervention outcome data.[78,79]

In routine medical practice, CBT has not yielded clinically significant outcomes for patients with ME/CFS.[80-82] Furthermore the lack of CBT providers who specialize in this illness (psychologist, social worker, or nurse) indicates that CBT may not be an option for many patients with ME/CFS. More detailed information on CBT protocols and the controversy surrounding its application in ME/CFS is presented elsewhere.[82,83]

5:8 Management of Related Conditions

Orthostatic intolerance (OI) and cardiovascular symptoms. Many patients have symptoms suggestive of OI, such as light-headedness, dizziness, palpitations and feeling faint. They are advised to rise slowly, particularly when getting up in the morning or during the night. Prolonged standing is to be avoided. The use of pressure stockings or elevating the legs while sitting may help to prevent pooling of blood in the limbs. In addition, recumbent exercise is often better tolerated (e.g., swimming, recumbent bicycle or exercise lying on the floor or bed).

Dietary management of OI is intended to increase blood volume. Extra salt or mixed electrolytes in the diet (salty foods, added table salt, salt tablets) along with increased oral fluid intake may help to overcome hypotension and postural tachycardia. This recommendation is equivalent to a pinch of plain salt every 2-3 hours throughout the day and a salty snack at bedtime. Salt and fluids should also be increased before and after exercise.

Fludrocortisone, 0.1-0.2 mg/day, can improve symptoms attributable to hypotension and hypovolemia in some patients, but it may not be effective long term. Blood pressure and electrolytes should be monitored regularly in patients taking fludrocortisone, with potassium supplementation if necessary. The risk of potassium depletion can be reduced by eating a banana or kiwifruit daily. Low dose beta-blockers, such as atenolol (25-50 mg) or propranolol (10-20 mg), can be useful in controlling tachycardia or palpitations associated with postural hypotension.

Gastrointestinal problems. Many patients with ME/CFS experience gastrointestinal symptoms including reflux, indigestion, nausea, vomiting, bloating, pain and irritable bowel syndrome. Slow gastric emptying may be present. In general, dietary management (see below) and conventional conservative symptomatic treatment are advised. A proportion of these patients will have gluten and/or lactose intolerance, fructose intolerance, other food sensitivities, or bacterial overgrowth of the small intestine. These possibilities should be excluded during the initial work up. Any change in gastrointestinal symptoms should be investigated.

Urinary symptoms. Many individuals with ME/CFS have urinary symptoms of frequency, dysuria and bladder pain. Once infection has been ruled out, other possibilities should be considered including interstitial cystitis, detruser instability, urethral syndrome or endometriosis. The treating physician may wish to refer the patient to a specialist for diagnosis and/or treatment.

Allergies. Many patients with ME/CFS suffer from allergies that may worsen symptoms during relapse. Treatment with nasal sprays, inhalers or topical skin applications may be adequate, but many will need to use an oral antihistamine. A non-sedating antihistamine can be used in the daytime and a sedating antihistamine at night. Allergy symptoms should not be confused with sensitivities or intolerances, which are not histamine-related.

Multiple chemical sensitivity (MCS). A number of patients with ME/CFS also have MCS. Rather than an allergic response, their sensitivity is to low levels of specific odors or chemicals, which cause an exacerbation of symptoms. For example, perfumes, cigarette smoke, cleaning products, paint, glue and many other odors may cause problems. These patients may need advice on how to avoid the environmental chemicals which trigger symptoms.[84] Patients with multiple food sensitivities who avoid food groups may need dietary counselling to rotate their foods to avoid malnutrition.

Infections and immunological factors. A number of viral, bacterial or parasitic infections have been found in some cases of ME/CFS (e.g., herpes viruses, enteroviruses, B. burgdorferi, mycoplasmas, G. lamblia).[15] Based on clinical observation, the use of long-term antibiotics, anti-parasitics or antiviral therapy may be beneficial in patients where the presence of pathogens has been confirmed.

Although initial results of some new drug therapies for various viral infections in ME/CFS appear promising,[85-89] treatment protocols are often complex and remain untested in controlled trials. In

addition, adverse reactions, the development of drug resistance, and costs are significant concerns. Referral to a specialist who has experience in testing and therapeutic interventions for these subgroups of patients may be helpful.

As immunological factors appear to play a role in ME/CFS, immune modulators such as isoprinosine (Imunovir®) may be helpful in selected patients. Specialist advice may be in order if clinical experience is limited. Based on two randomized trials,[90,91] the experimental drug rintatolimod (Ampligen®) has been shown to benefit patients who are more disabled. The drug is currently in Phase III clinical trials and not FDA approved. It is available to patients in the U.S. only through participation in an open-label, cost-recovery study and remains costly to patients who qualify. Finally, rituximab, an anti-CD20 monoclonal antibody primarily used as a cancer drug, has been found to be beneficial to patients with ME/CFS in a small randomized trial[117].

5:9 Dietary Management. Although no evidence-based special diet is available for ME/CFS, dietary programs are popular with many patients. Good nutrition with a balanced diet is advisable. Excesses of specific foods as well as rich, fatty foods, sugars and caffeine are best avoided. Eating small meals with snacking in between can be helpful. To help counteract the risk of osteoporosis from lack of vitamin D, dairy products should be incorporated in the diet if lactose intolerance or an allergic reaction to milk and milk products is not present. In addition, because alcohol intolerance (causing sedation) may be reported, alcohol use should be minimized or avoided.

Some individuals who attribute their ME/CFS to food intolerances will carefully avoid certain foods. Gluten and/or lactose intolerances, not uncommon in ME/CFS, require a gluten, or lactose-free diet. Provided that these intolerances have been excluded, a rotational approach, rather than absolute avoidance, may lessen possible negative reactions to food.

Although there is no evidence that patients with ME/CFS suffer from systemic candidiasis, diets intended to combat candidiasis and allergies are quite popular and many patients believe that they are helpful. Finally, some patients with gastrointestinal symptoms have reported benefit from a "leaky gut diet"[92] in combination with L-glutamine or butyrate.

Dietary supplements. Patients with ME/CFS need to ensure that they obtain at least the RDA of vitamins and minerals. This is not always possible using dietary sources. A suitable multivitamin and a separate multi-mineral preparation will ensure that at least the RDA of vitamins and minerals are obtained in the correct proportions.

Vitamin D. Because Vitamin D deficiency is often found in ME/CFS,[63] additional vitamin D may be necessary to achieve an optimal level, which may reduce the risk of osteoporosis, cancer, heart disease, stroke, and other illnesses.[93]

Vitamin B12 and B-Complex. Cerebrospinal fluid levels of vitamin B12 may be depleted in some patients with ME/CFS,[94] and a trial of a weekly injection of hydroxycobalamin 1000µg for six weeks (or perhaps longer) may be helpful. There are no reports of serious risk or side effects, despite the high blood levels achieved. A supplement of B-complex will avoid concurrent B vitamin deficiency.

Essential fatty acids. Essential fatty acids supplementation in ME/CFS has yielded symptom improvement and greater shifts towards normal levels of cell fatty acids concentration in treated patients in some studies.[95] Eicosapentanoic acid, an essential fatty acid, is a major component of omega-3 fish oil. This substance has been beneficial in reducing symptoms for some patients. Additional vitamin and mineral cofactors, including biotin, niacin, folic acid, vitamin B6, vitamin B12, vitamin C, selenium, zinc, and magnesium,[96] may be supportive in conjunction with essential fatty acids supplementation.

CoQ10. Research has shown that plasma CoQ10 is significantly lower in a substantial number of ME/CFS patients than in healthy controls and plasma levels are inversely related to the severity of ME/CFS symptoms.[122, 135] Clinical experience has found that some patients may show improvement after treatment with CoQ10 at a dosage of 100-400

mg daily. When effective, the supplement needs to be taken long term to maintain efficacy.

Zinc. Inadequate zinc intake may contribute to decreased function of natural killer cells and cell-mediated immune dysfunction.[97] A multi-mineral preparation may ensure the correct balance between zinc and copper.

Herbs. Patient use of herbal/natural remedies should be identified to reveal likely side effects and avoid potential conflicts with prescribed medications. Patients may not know that "natural" does not necessarily mean "better" or "safe." As with medication, small doses should be used initially with warnings about adverse reactions. Some herbs with pharmacological effects have been traditionally incorporated in the diet, e.g., herbal teas of peppermint, ginger or chamomile for gastrointestinal symptoms or for improving sleep.

Warnings are appropriate for several largely unregulated products. Glyco-nutrients, olive leaf and picnogenol (pine bark), have been touted as potential cures for ME/CFS, but neither clinical observation nor published evidence supports their use. Products claiming to be immune system boosters have not been shown, in the medical literature, to reduce symptoms in ME/CFS patients. Many of the so-called adrenal support concoctions contain steroids, which can have adverse effects in those who do not need them, especially when stopped suddenly. Steroids should only be prescribed by a physician.

5:10 Alternative and complementary approaches.
Some alternative and complementary treatments have been shown to be beneficial in some patients. Acupuncture, massage and chiropractic are relatively established treatments for pain, and are covered in the pain section. Unfortunately patients with ME/CFS often try costly, non-established and speculative treatments in hope of a cure. A review of studies of such non-established therapies revealed generally poor methodologies and little evidence for more than modest effects. Equivocal evidence was found for homeopathy and biofeedback. More detailed information may be found in recent reviews.[98,99]

5:11 Prognosis
The prognosis of ME/CFS is quite variable. The illness usually starts suddenly with "flu-like" symptoms, but in some patients, the onset is gradual and symptoms develop over months or years. Patients may be very ill at the onset of the illness, but the majority of patients report improvement, reaching a plateau, within five years of becoming ill. The severity of illness varies between the extremes of some patients who are completely bedbound and others who are able to go out to work. Remissions and relapses are common. Over time, many patients improve enough so that they no longer keep their ME/CFS diagnosis, but they also do not return to their premorbid level of functioning.[126,130] Restoration of full premorbid health is rare in adults, but more common in children.[130] Patients who do recover often need more rest than their contemporaries. Some patients may slowly get worse. Patients with ME/CFS who also have FM are less likely to improve than patients with ME/CFS alone.[129]

A review of 14 studies found on average that 5% of patients recovered (range 0–31%); 40% of patients improved during follow-up (range 8–63%); 8-30% returned to work; 5-20% of patients reported worsening[10]

Risk factors for severity of the illness are:
- The severity of the illness at onset[126]
- The standard of early management of the illness (e.g., late diagnosis or overexertion in the early stages of the illness are likely to lead to deterioration)[127,128]
- Having a mother with the illness[127]

Mortality. Death, in patients with ME/CFS, is usually certified as being caused by another co-existing illness and not as being caused by ME/CFS, so the mortality rate is difficult to determine. Preliminary data from one study found suicide, heart disease and cancer to be the leading causes of death in patients with ME/CFS and the mean ages of death from these causes were well below national averages.[132] Another study found that all-cause mortality rates of individuals with ME/CFS were not significantly different from standardized mortality rates.[131] The clinician and patient should remain

attentive to emergence of health concerns related to aging, immobility, other chronic illness and the potential of unknown outcomes of this illness that could lead to premature death.

5:12 Follow up

Patients with ME/CFS require regular reassessment and follow-up to manage their most disabling symptoms and to re-confirm or change the diagnosis. Although patients may assume that new symptoms are part of ME/CFS, other illnesses with symptoms not characteristic of ME/CFS can develop and should be investigated. Any patient who experiences a worsening of symptoms or the onset of new and/or additional symptoms should be encouraged to return to the physician's office. Additionally, an annual follow-up should be undertaken that includes a review of symptoms, a physical exam, a functional capacity evaluation, routine screening (Table 1), and a review of the patient's management/treatment plan.

6. RELATED CLINICAL CONCERNS

6:1 The Severely Ill/Lowest Functioning Patient: Special Considerations

Severely ill/very low functioning patients with ME/CFS (functional capacity 0-4, Appendix C) may be homebound, wheelchair dependent or bed bound for months or years. Some recover to varying degrees and are able to function outside their homes. Some functional patients may deteriorate and become homebound. Homebound patients are a distinct subgroup of patients with ME/CFS who require management specifically tailored to their needs.[125]

Little published data is available on the characteristics of this group because they can rarely make office visits, take phone calls, or answer research questionnaires. Although prevalence studies are not available for homebound patients, lay literature suggests that perhaps 25% of patients with ME/CFS are so disabled. This estimate is supported by a study using actigraphs in which 25% of a large sample of patients were found to have "pervasively passive activity patterns."[9]

Assessments of these patients reveal profound weakness and a high degree of symptom severity, especially: severe limitations to mental and physical activity; hypersensitivities to light, sound, touch, certain foods and medications. Other manifestations may include orthostatic intolerance; speech and swallowing difficulties; more comorbidities; atypical seizures (absence type) and myoclonus. Vitamin D deficiency may lead to osteopenia in housebound patients. Some of these patients may be very frightened and struggle with feelings of frustration, despair and anger. They may need to be helped to learn to adapt to their level of impairment.

Many homebound patients will require in-home assistance. Such care may be rendered by a family member or professional help. Home-based caregivers provide assistance with patient care, ongoing patient management and support of patient morale. Caregivers are often subject to considerable stress in serving the needs of the patients.

The most severely ill patients (0 on the Functional capacity Scale - Appendix C) may be bedbound and exhibit at least some of these symptoms and impairments:

- Almost constant pain that may worsen from even mild stimulation (e.g., light touch)
- Severe nausea and inability to feed themselves which may necessitate tube feeding
- Inability to walk to or use the toilet, possibly requiring use of a bottle, bedpan, diapers or catheter
- Inability to take a shower and difficulty tolerating a bed bath
- Hypersensitivity to light, sound and odors
- Difficulty communicating their needs to a caregiver, due to speech difficulties or exhaustion
- Inability to turn over in bed
- Episodes of limb immobility
- Short term memory impairment
- Inability at times to recognize their friends
- Poor tolerance of many medications at the usual dosage

Considerations that may be helpful for the severely ill include:

- A very quiet environment, that may include a darkened room or wearing eye pads
- Limiting mental activity (such as reading, writing, computing, or concentrating) because mental exertion is as exhausting as physical activity in many of these patients
- Minimizing medications and supplements to those absolutely necessary
- Prescribing medications in very low doses and titrating slowly, as tolerated
- Adjusting expectations for themselves and from others to very modest levels

In addition, housebound, low functioning patients may require more services and support with respect to:

- Pain management
- Follow up (perhaps via home visits and telephone or online contacts)
- Social support (including home health services and aides perhaps using a nurse manager)
- Respite care
- Stress management and grief/loss counseling (perhaps by telephone)
- Balanced nutrition and healthy foods (provided and prepared by caregivers)
- Physical /occupational therapy

Ampligen®/rintatolimod has been studied experimentally in the severely ill, and has shown promise in some patients (see section 5:8).

Activity management

Instructing severely ill patients to exercise usually leads to post-exertional worsening of symptoms. Nevertheless, activity management is important. For patients confined to bed, activity may be limited to whatever necessary actions are tolerated. For those who can tolerate touch, gentle, passive range-of-motion activity and gentle stretching can be provided by a knowledgeable physical therapist. Passive stretching for brief periods of time (1 minute at a time followed by a rest) can help to reduce stiffness, maintain joint range of motion, prevent contractures and combat deconditioning. The initial aim can be to gradually reach the goal of moving every muscle at least once daily. Movement or activity when lying flat is always easier. Some patients may improve to the point where they can progress to standing up and then minimal leisurely walking, for five minutes daily. Other patients continue be bedbound. The activity program should allow the severely ill ME/CFS patients to pace themselves and stay within their energy envelope - however small it is.

Irma Pinxterhuis, in her studies of the very severely ill,[100] remarked, “They needed above all peace of mind and a feeling that they and their families were taken care of, so that they could use all their energy on getting better.”

6:2 Pregnancy

Most mothers with ME/CFS have an uneventful pregnancy and deliver a normal child. During pregnancy, ME/CFS symptoms may improve for some, remain the same for some, and worsen for others. In many patients, symptoms return to pre-pregnancy levels within weeks of delivery. Pregnancy is not recommended in the early stages of ME/CFS, because the patient may be very ill and the diagnosis uncertain.

Some medications for ME/CFS can damage a growing fetus especially in the early stages of pregnancy. The effects of most herbal preparations on the fetus are unknown. Healthcare providers should advise which ongoing medications, given their risks to the fetus, should be stopped before a planned pregnancy. The patient can then determine if she can cope with possibly worsened ME/CFS symptoms without the medications. Some essential medications may need to be continued in smaller doses.

Obstetric problems, which may be more prevalent in women with ME/CFS, include lowered fertility, miscarriage, severe vomiting in pregnancy, exhaustion in labor, delayed post-partum recovery and post-partum depression.[101,102] During labor, adequate pain relief is important and prompt surgical delivery of the child is recommended before the mother becomes exhausted or if labor is prolonged.

Lactation is not contraindicated. The advantages and disadvantages of breast-feeding should be discussed with the mother. Milk can be expressed for night feedings, to allow the mother adequate rest.

Although the majority of children born to women with ME/CFS are healthy, they may have a higher risk of developing ME/CFS than the general population. One study showed a 5% risk of developing ME/CFS in childhood or early adult life.[12] Another small study suggests that the offspring also may have an increased risk of developmental delays and learning difficulties.[101] Child-rearing is often the biggest challenge for parents with ME/CFS and many require a good support network.

For further information on pregnancy in patients with ME/CFS, see references.[134, 136]

6:3 Gynecological Problems

ME/CFS and some common gynecological conditions such as pre-menstrual syndrome and menopause show a significant overlap of symptoms. These conditions also frequently exacerbate symptoms of ME/CFS and vice versa.

A small number of scientific studies suggest that several gynecological conditions occur more frequently in women with ME/CFS. Some of these conditions may pre-date the onset of the illness. These disorders include: premenstrual syndrome; anovulatory and oligo-ovulatory cycles; low estrogen levels leading to a multitude of CNS symptoms, loss of libido, and in later years, osteoporosis; dysmenorrhea; pelvic pain; endometriosis; interstitial cystitis; dyspareunia and vulvodynia; and a history of hysterectomy (for fibroids or ovarian cysts).[103,104,133] The investigation and treatment of these conditions should follow standard gynecological practice.

Sexual dysfunction is present in up to 20% of patients with ME/CFS.[28] Sexual activity in both women and men can be inhibited by fatigue, and post-exertional symptom exacerbation following sexual activity. In women sexual activity may also be inhibited by dyspareunia due to pelvic pain or vulvodynia and by loss of libido. If a low estrogen state is present, giving hormone replacement therapy orally or as a vaginal cream can be helpful.[134]

Many peri-menopausal and post-menopausal patients with ME/CFS may benefit from hormone replacement therapy (HRT). Premenopausal patients with ME/CFS and low estrogen levels may also be helped by HRT. Estrogen may improve cerebral circulation, benefit cognition, and provide significant relief from hot flashes, insomnia, and fatigue. HRT also reduces the risk of osteoporosis.[105]

Some women may be more responsive to a progesterone-only regimen such as a progesterone-only pill, or impregnated intra-uterine device. These approaches also address contraception, which may be vital for women with ME/CFS. Oral contraceptives may help patients who suffer from menstrual pain, particularly if bleeding is heavy.

Hormonal therapy should be limited in duration due to the increased risk of breast, ovarian and uterine cancer with HRT. Some women prefer to take "natural" hormones (e.g. phytoestrogens and wild yam products), but it should be pointed out that prospective randomized studies of their clinical effects and potential side effects have not been done.[106,107]

6:4 Pediatric ME/CFS

ME/CFS can occur at any age but it is difficult to diagnose under the age of ten. Children and adolescents sometimes do not report symptoms and assume their feeling of tiredness is normal. In addition, they are often misdiagnosed as lazy or having behavioral disorders, school phobia, ADHD or factitious disorder by proxy.[108,109] The diagnosis of ME/CFS is often overlooked or delayed, but it can be established using a specific pediatric case definition[110] (Appendix B), which is based on the Canadian case definition. The diagnosis in children and adolescents under the age of 18 can be made after 3 months of illness. The prevalence of ME/CFS in children and adolescents varies greatly in different studies, but overall, rates appear to be lower than in adults. The gender ratio is almost equal in children compared to a female preponderance in adults. Children often have more autonomic dys-

function than adults, with orthostatic intolerance being common.

Management and treatment of children with ME/CFS is similar to that described above for adults, but pediatric management can be especially challenging. Any medications should be prescribed with great caution. As with adults, many pediatric patients with ME/CFS respond to much lower than standard doses of medications.[134]

Most children with ME/CFS experience worsening of their school performance. In the USA, children and adolescents with cognitive deficits and physical limitations may qualify for special services. Patients with ME/CFS qualify for accommodations/ modifications under the category of "Other Health Impairment" under the (US) Individuals with Disabilities Education Act (IDEA), and/or Section 504 of the Rehabilitation Act, 1973. With physician documentation, eligible students can receive an individualized educational plan (IEP) or 504 plan, which require the school to provide home tutoring and/or reasonable accommodations.

Tutoring at home or online or home schooling allows students who are debilitated with ME/CFS to pace themselves and reduce symptom flares. When appropriate and in conjunction with school personnel who are willing to work with the child and family, a graduated schedule of return to school can be successful. This might involve the child initially attending a single class on a daily basis and gradually increasing the number of classes attended over several weeks or months. Other options may include: rest in the nurses office, use of school elevators, double sets of books one for home and one at school, more time to take tests, home tutoring during ME/CFS flare-ups, online coursework, and restricted or no participation in gym classes.

To enhance the chances of recovery, competitive sports are best avoided. If the patient is subject to stress-related symptom flare-ups, it may be desirable to limit academic examinations to those that are deemed essential. Family counseling may be recommended if family conflicts related to the child's illness are evident.[134] The prognosis for children with ME/CFS is considerably better than for adults, although they may initially be severely ill.[111-113]

6:5 Immunizations

Patients with ME/CFS should consider avoiding all but essential immunizations particularly with live vaccines, as post-vaccination relapse has been known to occur. Usual medical practice is not to vaccinate a normally healthy person when unwell. However, during a flu epidemic, patients should balance the health hazards of becoming ill against the possibility of symptom-worsening due to immunization.

6:6 Blood and Tissue Donation

The American Red Cross requires that blood donors "be healthy", i.e., feel well and be able to perform normal activities.[119] Since people with ME/CFS are not healthy by this definition, they should not donate blood. Furthermore, given the possibility of infectious disease transmission in patients with ME/CFS, many countries have deferred or prohibited blood and tissue donation from patients who have ever suffered from ME/CFS.[119]

6:7 Recommendations Prior to Surgery

For individuals with ME/CFS approaching surgery, discussion with the surgeon and anesthesiologist/ anesthetist about this illness is important. Issues such as depleted blood volume, orthostatic intolerance, pain control, subnormal body temperature, dry eyes and mouth, multiple chemical sensitivities, sensitivity to anesthetic medications and extended recovery and rehabilitation times should be addressed. Further recommendations for persons with ME/CFS who are anticipating surgery are given in Appendix E.

7. REFERENCES

1. Carruthers BM, et al. Myalgic Encephalomyelitis/Chronic Fatigue Syndrome: Clinical Working Case Definition, Diagnostic and Treatment Protocols. *J CFS* 2003; 11(1):7-116.
2. Jason LA, et al. Attitudes regarding chronic fatigue syndrome: The importance of a name. *Journal of Health Psychology* 2001; *6*:61-71.
3. "International Classification of Diseases (ICD)". World Health Organization. http://www.who.int/classifications/icd/en/.
4. Fukuda K, et al. The chronic fatigue syndrome: a comprehensive approach to its definition and study. International Chronic Fatigue Syndrome Study Group. *Ann Intern Med.* 1994 Dec 15; 121(12):953-9.
5. Hyde B. The clinical and scientific basis of Myalgic Encephalomyelitis/Chronic Fatigue Syndrome. Nightingale Research Foundation, 1992. p176-186.
6. Jason LA, et al. A community-based study of chronic fatigue syndrome. *Arch Intern Med.* 1999. Oct 11; 159(18):2129-37.
7. Carruthers, BM et al. (2011). Myalgic Encephalomyelitis: International Consensus Criteria. *J Intern Med.* 2011 Oct;270(4):327-38.
8. Reyes M, et al. Prevalence and incidence of chronic fatigue syndrome in Wichita, Kansas. *Arch Intern Med*. 2003 Jul 14; 163(13):1530-6.
9. Van der Werf SP, et al. Identifying physical activity patterns in chronic fatigue syndrome using actigraphic assessment. *J Psychosomatic Research* 2000 Nov; 49(5):373-9.
10. Cairns R, Hotopf MA. Systematic review describing the prognosis of chronic fatigue syndrome. *Occup Med (Oxford, England)*. 2005 Jan; 55(1):20-31.
11. Hohn O, Bannert N. Origin of XMRV and its demise as a human pathogen associated with chronic fatigue syndrome. *Viruses* 2011 Aug; 3(8):1312-9.
12. Underhill R, O'Gorman R. The prevalence of Chronic Fatigue Syndrome and chronic fatigue among family members of CFS patients. *J CFS* 2006; 13(1):3-13.
13. Buchwald MD et al. A twin study of chronic fatigue. *Psychosomatic Med* 2001; 63:936-943.
14. Albright F, et al. Evidence for a heritable predisposition to Chronic Fatigue Syndrome. *BMC Neurol.* 2011 May 27; 11:62.
15. Bansal AS, et al. Chronic fatigue syndrome, the immune system and viral infection. [Rev] *Brain, Behavior, & Immunity* 2012 Jan; 26(1):24-31.
16. Salit IE. Precipitating factors for the chronic fatigue syndrome. *J Psychiatric Research* 1997 Jan-Feb; 31(1):59-65.
17. Hickie I, et al. Group DIOS. Post-infective and chronic fatigue syndromes precipitated by viral and non-viral pathogens: prospective cohort study. *BMJ* 2006; 333:575.
18. VanNess JM, et al. Diminished Cardiopulmonary Capacity During Post-Exertional Malaise in Chronic Fatigue Syndrome. *J CFS* 2007; 14(2):77-86.
19. Jones DE, et al. Loss of capacity to recover from acidosis on repeat exercise in chronic fatigue syndrome: a case-control study. *Eur J Clin Invest.* 2012 Feb; 42(2):186-94.
20. Wong R, et al. Skeletal muscle metabolism in the chronic fatigue syndrome. In vivo assessment by 31P nuclear magnetic resonance spectroscopy. *Chest* 1992 Dec; 102(6):1716-22
21. Light AR, et al. Moderate exercise increases expression for sensory, adrenergic, and immune genes in chronic fatigue syndrome patients but not in normal subjects. *J Pain* 2009 Oct; 10(10):1099-112.
22. Light AR, et al, Gene expression alterations at baseline and following moderate exercise in patients with Chronic Fatigue Syndrome and Fibromyalgia Syndrome. *J Intern Med*. 2012 Jan; 271(1):64-81.
23. Vermeulen RC, et al. Patients with chronic fatigue syndrome performed worse than controls in a controlled repeated exercise study despite a normal oxidative phosphorylation capacity. *J Transl Med*. 2010 Oct 11; 8:93.
24. Torres-Harding S, et al. Evidence for T-helper 2 shift and association with illness parameters in chronic fatigue syndrome (CFS). *Bull IACFS ME* 2008 Fall; 16(3):19-33.
25. Fletcher MA, et al. Plasma cytokines in women with chronic fatigue syndrome. *J Transl Med*. 2009 Nov 12; 7:96.
26. Klimas NG, et al. Immunologic abnormalities in chronic fatigue syndrome. *J Clin Microbiol.* 1990 Jun; 28(6):1403-10.
27. Suhadolnik R, et al. Biochemical evidence for a novel low molecular weight 2-5A dependent RNase L in chronic fatigue syndrome. *J Interferon and Cytokine res*. 1997; 17: 377-385.
28. Bell DS. The Doctor's Guide to Chronic Fatigue Syndrome. Addison-Wesley. 1995; p 11.
29. Patarca R. Cytokines and chronic fatigue syndrome. *Annals of the New York Academy of Sciences* 2001 Mar; 933:185-200.
30. Papadopoulos AS, Cleare AJ. Hypothalamic-pituitary-adrenal axis dysfunction in chronic fatigue syndrome. *Nat Rev Endocrinol.* 2011 Sep 27; 8(1):22-32.
31. Fuite J, et al. Neuroendocrine and immune network re-modeling in chronic fatigue syndrome: an exploratory analysis. *Genomics* 2008 Dec; 92(6):393-9.

32. De Becker P, et al. Dehydroepiandrosterone (DHEA) response to i.v. ACTH in patients with chronic fatigue syndrome. *Horm Metab Res.* 1999 Jan; 31(1):18-21.
33. Allain TJ, et al. Changes in growth hormone, insulin, insulin like growth factors (IGFs), and IGF-binding protein-1 in chronic fatigue syndrome. *Biol Psychiatry.* 1997 Mar 1; 41(5):567-73.
34. Chaudhuri A, et al. Chronic fatigue syndrome: A disorder of central cholinergic transmission. *J CFS* 1997; 3:3-16
35. Sharpe M, et al. Increased prolactin response to Buspirone in chronic fatigue syndrome. *J Affect Disord.* 1996 Nov 4; 41(1):71-6.
36. Bakheit AM, et al. Abnormal arginine-vasopressin secretion and water metabolism in patients with postviral fatigue syndrome. *Acta Neurol Scand* 1993; 87(3):234-8.
37. Boneva RS, et al. Higher heart rate and reduced heart rate variability persist during sleep in chronic fatigue syndrome: a population-based study. *Auton Neurosci.* 2007 Dec 30; 137(1-2):94-101.
38. Fletcher MA, et al. Plasma neuropeptide Y: a biomarker for symptom severity in chronic fatigue syndrome. *Behav Brain Funct* 2010; 6:76.
39. De Lange FP, et al. Gray matter volume reduction in the chronic fatigue syndrome. *Neuroimage* 2005 Jul 1; 26(3):777-81.
40. Buchwald MD, et al. A chronic illness characterized by fatigue, neurologic and immunologic disorders, and active human herpes type 6 infection. *Annals Int Med.* 1992; 116:103-13.
41. Lange G, et al. Brain MRI abnormalities exist in a subset of patients with chronic fatigue syndrome. *J Neurol Sci.* 1999 Dec 1; 171(1):3-7.
42. Yoshiuchi K, et al. Patients with chronic fatigue syndrome have reduced absolute cortical blood flow. *Clin Physiol Func Imag* 2006; 26(2):83-6.
43. Costa DC, et al. Brainstem perfusion is impaired in chronic fatigue syndrome. *QJM.* 1995 Nov; 88(11):767-73.
44. De Lange FP, et al. Neural correlates of the chronic fatigue syndrome. *Brain* 2004; 127:1948-49.
45. Tanaka M, et al. Reduced responsiveness is an essential feature of chronic fatigue syndrome: A fMRI study. *BMC Neurol.* 2006; 6:9.
46. Mathew SJ, et al. Ventricular cerebrospinal fluid lactate is increased in chronic fatigue syndrome compared with generalized anxiety disorder: an in vivo 3.0 T (1)H MRS imaging study. *NMR Biomed* 2009; 22(3):251-8.
47. Shungu DC, et al. Increased ventricular lactate in chronic fatigue syndrome. III. Relationships to cortical glutathione and clinical symptoms implicate oxidative stress in disorder pathophysiology. *NMR Biomed.* 2012 Jan 27.
48. Van Hoof E, et al. Defining the occurrence and influence of alpha-delta sleep in chronic fatigue syndrome. *Am J Med Sci.* 2007 Feb; 333(2):78-84.
49. Natelson BH, et al. Spinal fluid abnormalities in patients with chronic fatigue syndrome. *Clin Diag Lab Immunol.* 2005; 12(1):52-5.
50. Glass JM. Cognitive dysfunction in fibromyalgia and chronic fatigue syndrome: new trends and future directions. *Curr Rheumatol Rep.* 2006 Dec; 8(6):425-9.
51. Michiels V, Cluydts R. Neuropsychological functioning in chronic fatigue syndrome: a review. *Acta Psychiatr Scand.* 2001 Feb; 103(2):84-93.
52. Constant EL, et al. Cognitive deficits in patients with chronic fatigue syndrome compared to those with major depressive disorder and healthy controls. *Clinical Neurology & Neurosurgery* 2011 May; 113(4):295-30.
53. LaManca J, et al. Influence of exhaustive treadmill exercise on cognitive functioning in chronic fatigue syndrome. *Am J Med*. 1998; 105: 59S-65S
54. Lerner AM, et al. Repetitively negative changing T waves at 24-h electrocardiographic monitors in patients with the chronic fatigue syndrome. Left ventricular dysfunction in a cohort. *Chest* 1993 Nov; 104(5):1417-21.
55. Miwa K, Fujita M. Small heart syndrome in patients with chronic fatigue syndrome. *Clin Cardiol.* 2008 Jul; 31(7):328-33.
56. Streeten DH, et al. The roles of orthostatic hypotension, orthostatic tachycardia and subnormal erythrocyte volume in the pathogenesis of the chronic fatigue syndrome. *Am J Med* 2000; 320:1-8.
57. Myhill S, et al. Chronic fatigue syndrome and mitochondrial dysfunction. *Int J Clin Exp Med* 2009; 2(1): 1–16.
58. Whistler T, et al. Exercise responsive genes measured in peripheral blood of women with chronic fatigue syndrome and matched control subjects. *BMC Physiol.* 2005 Mar 24; 5(1):5.
59. Behan WM, et al. Mitochondrial abnormalities in the postviral fatigue syndrome. *Acta Neuropathol.* 1991; 83(1):61-5.
60. Murrough JW, et al. Increased ventricular lactate in chronic fatigue syndrome measured by 1H MRS imaging at 3.0 T. II: comparison with major depressive disorder. *NMR Biomed.* 2010 Jul; 23(6):643-50.
61. Kerr JR. Gene profiling of patients with chronic fatigue syndrome/myalgic encephalomyelitis. *Curr Rheumatol Rep.* 2008 Dec; 10(6):482-91.

62. Kerr JR, et al. Gene expression subtypes in patients with chronic fatigue syndrome/myalgic encephalomyelitis. *J Infect Dis.* 2008 Apr 15; 197(8):1171-84.
63. Berkovitz S, et al. Serum 25-hydroxy vitamin D levels in chronic fatigue syndrome: a retrospective survey. *Int J Vitam Nutr Res.* 2009 Jul; 79(4):250-4.
64. Friedberg F & Jason LA. Understanding chronic fatigue syndrome: An empirical guide to assessment and treatment. Washington, D.C.: American Psychological Association; 1998. Chapter 7: Differential diagnosis in CFS; p. 99-118.
65. Hawk C, et al. Differential diagnosis of chronic fatigue syndrome and major depressive disorder. *Int J Behavioral Medicine* 2006; 13(3):244-51.
66. Taylor DJ, Roane BM. Treatment of insomnia in adults and children: a practice-friendly review of research. *J Clinical Psychology* 2010 Nov; 66(11): 1137-47.
67. Carrier J, Dumont M. Sleep propensity and sleep architecture after bright light exposure at three different times of day. *J Sleep Res.* 1995 Dec; 4(4):202-211.
68. Sim J, Adams N. Systematic review of randomized controlled trials of non-pharmacological interventions for fibromyalgia. *Clin J Pain.* 2002 Sep-Oct; 18(5):324-36.
69. Turk DC, et al. Fibromyalgia: combining pharmacological and non-pharmacological approaches to treating the person, not just the pain. *J Pain* 2008 Feb; 9(2):99-104.
70. Jason, LA, et al. Classification of myalgic encephalomyelitis/chronic fatigue syndrome by types of fatigue. *Behavioral Medicine* 2010 Jan-Mar; 36(1):24-31.
71. Jason L.A, et al. The impact of energy modulation on physical functioning and fatigue severity among patients with ME/CFS. *Patient Education and Counseling* 2009; *77,* 237–241.
72. Davenport TE, et al. Conceptual model for physical therapist management of chronic fatigue syndrome/myalgic encephalomyelitis. *Phys Ther*. 2010 Apr; 90(4):602-14.
73. Stevens SR, Davenport TE. Functional outcomes of anaerobic rehabilitation in an individual with chronic fatigue syndrome: case report with 1-year follow-up. Bulletin of the International Association for Chronic Fatigue Syndrome/Myalgic Encephalomyelitis 2010. 18(3): 93-98.
74. Friedberg F, Sohl S. Cognitive-behavior therapy in chronic fatigue syndrome: is improvement related to increased physical activity? *J Clin Psychol.* 2009 Apr; 65(4):423-42.
75. Ray C et al. Life-events and the course of chronic fatigue syndrome. *British Journal of Medical* Psychology 1995; 68:323–331,
76. Friedberg F. Chronic fatigue syndrome, fibromyalgia, and related illnesses: A clinical model of assessment and intervention. *J Clinical Psychology* 2010; 6:641-665.
77. Friedberg, 2006. Fibromyalgia and chronic fatigue syndrome: Seven proven steps to less pain and more energy. Oakland, CA: New Harbinger.
78. Price JR, et al. Cognitive behavioural therapy for chronic fatigue syndrome in adults. Cochrane Database Syst Rev. 2008 Jul 16; (3):CD001027.
79. White PD, et al. Comparison of adaptive pacing therapy, cognitive behaviour therapy, graded exercise therapy, and specialist medical care for chronic fatigue syndrome (PACE): a randomised trial. *Lancet* 2011 Mar 5; 377(9768):823-36.
80. Whitehead L, & Campion P. Can general practitioners manage chronic fatigue syndrome? A controlled trial. *J CFS* 2002; 10:55-64.
81. Huibers MJ, et al. Efficacy of cognitive-behavioural therapy by general practitioners for unexplained fatigue among employees: Randomised controlled trial. *Brit J Psychiatry* 2004; 184:240-246.
82. Bleijenberg G. et al. (2003). Cognitive-Behavioral therapies. In L. A. Jason, P.A. Fennell & R.R. Taylor (Eds.). *Handbook of Chronic Fatigue Syndrome*. Hoboken, NJ: Wiley.
83. Kindlon T. Reporting of Harms Associated with Graded Exercise Therapy and Cognitive Behavioural Therapy in Myalgic Encephalomyelitis/Chronic Fatigue Syndrome. *Bulletin of the IACFS/ME* 2011; 19(2)
84. Magill MK, Suruda A. Multiple chemical sensitivity syndrome. *American Family Physician* 1998 Sep 1; 58(3):721-8.
85. Kerr JR, et al. Successful intravenous immunoglobulin therapy in 3 cases of parvovirus B19-associated chronic fatigue syndrome. *Clin Infect Dis.* 2003 May 1; 36(9).
86. Kogelnik AM et al. Use of valganciclovir in patients with elevated antibody titers against Human Herpesvirus-6 (HHV-6) and Epstein-Barr Virus (EBV) who were experiencing central nervous system dysfunction including long-standing fatigue. *J Clin Virol* 2006; 37 Suppl 1:S33-8.
87. Lunn MR, et al. Antiviral Therapy Induces Viral and Clinical Response in Patients with Central Nervous System Dysfunction and Chromosomally Integrated Human Herpesvirus-6. In: 6th International Conference on human herpes viruses 6 and 7 (HHV-6 & 7); 2008 June 21-25; Baltimore.

88. Chia J, Chia A. Rifampin augments the effects of oxymatrine in patients with myalgic encephalitis/chronic fatigue syndrome. In: International Association for Chronic Fatigue Syndrome/Myalgic Encephalitis (IACFS/ME) - Biennial International Conference; 2011; Ottawa, Canada; 2011.
89. Lerner AM, et al. Subset-directed antiviral treatment of 142 herpesvirus patients with chronic fatigue syndrome. *Virus Adaptation and Treatment* 2010; 2:47-57.
90. Strayer DR, et al. A controlled clinical trial with a specifically configured RNA drug, poly(I).poly(C12U), in chronic fatigue syndrome. *Clin Infect Dis.* 1994 Jan; 18 Suppl 1:S88-95.
91. Strayer DR, et al. A double-blind, placebo-controlled, randomized, clinical trial of the TLR-3 agonist rintatolimod in severe cases of chronic fatigue syndrome. PLoS ONE 2012; 7(3):e31334. doi:10.1371/journal.pone.0031334
92. Maes M, Leunis JC. Normalization of leaky gut in chronic fatigue syndrome (CFS) is accompanied by a clinical improvement: effects of age, duration of illness and the translocation of LPS from gram-negative bacteria. *Neuro Endocrinol Lett.* 2008 Dec; 29(6):902-10.
93. Moreno LA et al. Health effects related to low vitamin D concentrations: beyond bone metabolism. *Ann Nutr Metab.* 2011; 59(1):22-7.
94. Regland B, et al. Increased concentrations of homocysteine in the cerebrospinal fluid in patients with fibromyalgia and chronic fatigue syndrome. *Scand J Rheumatol.* 1997; 26(4):301-7.
95. Puri BK. The use of eicosapentaenoic acid in the treatment of chronic fatigue syndrome. *Prostaglandins Leukot Essent Fatty Acids.* 2004 Apr; 70(4):399-401.
96. Puri BK. Long-chain polyunsaturated fatty acids and the pathophysiology of myalgic encephalomyelitis (chronic fatigue syndrome*). J Clin Pathol.* 2007; 60:122-124.
97. Prasad AS. Zinc: mechanisms of host defense. *J Nutr.* 2007 May; 137(5):1345-9.
98. Alraek T, et al. Complementary and alternative medicine for patients with chronic fatigue syndrome: a systematic review. *BMC Complementary & Alternative Medicine* 2011; 11:87.
99. Porter N, et al. Alternative medical interventions used in the treatment and management of myalgic encephalomyelitis/chronic fatigue syndrome and fibromyalgia. *J Alternative and Complementary Medicine* 2010; 16(3):235-249.
100. Pinxterhuis I, "Chronic Fatigue Syndrome – Three Case Studies Concerning the Very Severely Ill." Poster presentation, IACFS 10th International Conference, Ottawa Canada, September 2011.
101. Schacterle S, Komaroff AL. A Comparison of Pregnancies that occur before and after the onset of chronic fatigue syndrome. *Arch Intern Med* 2004; 164:401-404.
102. Studd J, Panay N. Chronic fatigue syndrome. *Lancet* 1996; 348: 1384.
103. Harlow BL, et al. Reproductive correlates of chronic fatigue syndrome. *AJM* 1998; 105(3A), 94s-99s.
104. Reyes M, et al. Risk factors for CFS. *J CFS* 1996; 2,(4):17-33.
105. Bhavnani BR, Strickler RC. Menopausal hormone therapy. *J Obstet Gynaecol Can*. 2005 Feb; 27(2):137-62.
106. Kelley KW, Carroll DG. Evaluating the evidence for over-the-counter alternatives for relief of hot flashes in menopausal women. *J Am Pharm Assoc.* 2010 Sep-Oct; 50(5).
107. Cassidy A. Potential risks and benefits of phytoestrogen-rich diets. *Int J Vitam Nutr Res.* 2003 Mar; 73(2):120-6.
108. Jason LA, et al. Examining criteria to diagnose ME/CFS in pediatric samples. *J Behav Health & Med.* 2010; *1(3):*186-195.
109. Bell DS, et al. A Parent's Guide to Cfids: How to Be an Advocate for Your Child With Chronic Fatigue Immune Dysfunction. 1999 Informa Healthcare; London.
110. Jason LA, et al. A Pediatric Case Definition for Myalgic Encephalomyelitis and Chronic Fatigue Syndrome. *J. CFS* 2006; 13(2/3):1-28.
111. Carter BD, Marshall G S. New developments: Diagnosis and management of chronic fatigue in children and adolescents. *Current Problems in Pediatrics* 1995; *25*:281-293.
112. Rangel L, et al. The course of severe chronic fatigue syndrome in childhood. *J R Soc Med*. 2000 Mar; 93(3):129-34.
113. Jordan K M, et al. Research with children and adolescents with chronic fatigue syndrome: Methodologies, designs, and special considerations. *J CFS.* 1997; 3:3-1.
114. Vreeburg SA, et al. Major depressive disorder and hypothalamic-pituitary-adrenal axis activity: results from a large cohort study. *Arch Gen Psychiatry*. 2009 Jun;66(6):617-26
115. Lorenzetti V, et al. Structural brain abnormalities in major depressive disorder: a selective review of recent MRI studies. *J Affect Disord.* 2009 Sep;117(1-2):1-1
116. Komaroff AL. Buchwald DS. Chronic fatigue syndrome: an update [Review]. *Annual Review of Medicine* 1998; 49:1-13.

117. Fluge O, et al. Benefit from B-lymphocyte depletion using the anti-CD20 antibody rituximab in chronic fatigue syndrome. A double-blind and placebo-controlled study. PLoS ONE 2011; 6(10):e26358.
118. Bested A.C. et al. Hope and Help for Chronic Fatigue and Fibromyalgia, 2nd Edition, Sourcebooks.
119. American Red Cross. Eligibility requirements for blood donation 2012. http://www.redcrossblood.org/donating-blood/eligibility-requirements (accessed May 14,2012)
120. Fletcher MA, et al. Biomarkers in chronic fatigue syndrome: evaluation of natural killer cell function and dipeptidyl peptidase IV/CD26. PLoS ONE. 2010 May 25;5(5):e10817.
121. Siegel, DS, et al. Impaired natural immunity, cognitive dysfunction, and physical symptoms in patients with chronic fatigue syndrome: preliminary evidence for a subgroup? *J Psychosom Res.* 2006; 60(6):559-66.
122. Maes M, et al. Coenzyme Q10 deficiency in myalgic encephalomyelitis/chronic fatigue syndrome (ME/CFS) is related to fatigue, autonomic and neurocognitive symptoms and is another risk factor explaining the early mortality in ME/CFS due to cardiovascular disorder. *Neuro Endocrinol Lett.* 2009; 30(4):470-6.
123. Scott LV, Dinan TG. Urinary free cortisol excretion in chronic fatigue syndrome, major depression and in healthy volunteers. *J Affect Disord.* 1998 Jan; 47(1-3):49-54.
124. Peuhkuri K et al. Diet promotes sleep duration and quality. *Nutr Res.* 2012 May; 32(5):309-19.
125. Wiborg, JF, et al. Being homebound with chronic fatigue syndrome: A multidimensional comparison with outpatients. *Psychiatry Res.* 2010 May 15; 177(1-2):246-9.
126. Brown MM, et al. Understanding long-term outcomes of chronic fatigue syndrome. *J Clin Psychol.* 2012 Sep; 68(9):1028-35.
127. Pheby D and Saffron L. Risk factors for severe ME/CFS. *Biology and Medicine* (2009); 1 (4): 50 – 74.
128. Bell DS. Twenty-five year follow-up in chronic fatigue syndrome: Rising Incapacity. Mass CFIDS Assoc. Continuing Education Lecture April 16, 2011.
129. Ciccone DS, et al. Illness trajectories in the chronic fatigue syndrome: a longitudinal study of improvers versus non-improvers. *J Nerv Ment Dis*. 2010 Jul; 198(7):486-93.
130. Joyce J, et al. The prognosis of chronic fatigue and chronic fatigue syndrome: a systematic review. *QJM* 1997; 90 (3): 223–33.
131. Smith WR et al, Mortality in a cohort of chronically fatigued patients. *Psychological Medicine* 2006; 36 (9): 1301–6.
132. Jason LA. Causes of death among patients with chronic fatigue syndrome. *Health Care Women Int.* 2006 Aug;27; (7):615-26.
133. Boneva RS, et al. Gynecological history in chronic fatigue syndrome: a population-based case-control study. *J Womens Health (Larchmt)*. 2011 Jan; 20(1):21-8.
134. John J, Oleske J. (Eds.). A Consensus Manual for the Primary Care and Management of Chronic Fatigue Syndrome. (1st ed.) Lawrenceville, NJ: Academy of Medicine of NJ. 2002; p45-48, p51-56. Also online at http://www.nj.gov/health/fhs/documents/cfs_consensus_manual.pdf.
135. Morris G, et al. Coenzyme Q10 Depletion in Medical and Neuropsychiatric Disorders: Potential Repercussions and Therapeutic Implications. *Mol Neurobiol.* 2013 Jun 13. [Epub ahead of print]
136. Allen PR. Chronic Fatigue Syndrome: Implications for women and their health care providers during the childbearing years. *J of Midwifery & Women's Health* 2008; 53 (4): 289-301

APPENDICES

A 1994 International research case definition (Fukuda K, et al.[4]) worksheet
B Pediatric case definition (Jason LA, et al.[110]) worksheet
C Functional capacity scale
D Activity log
E Recommendations prior to surgery
F Fact sheet on ME/CFS (handout)

APPENDIX A

1994 RESEARCH CASE DEFINITION CRITERIA FOR CHRONIC FATIGUE SYNDROME WORKSHEET*

Patient name -------------------------------- Date --------------------------------

Major Criteria
-------- Significant fatigue, relapsing or chronic, insidious or abrupt, of at least six months duration
-------- Exclusion of other clinical conditions that plausibly explain this fatigue

Minor Criteria (A minimum of 4 out of 8)
Symptoms (must be concurrent, persisting or relapsing; and symptoms must NOT precede the onset of fatigue)

-------- Sore throat
-------- Painful lymph nodes (cervical, axillary, inguinal, or supraclavicular)
-------- Generalized, new headaches
-------- Myalgia or muscle discomfort
-------- Migratory arthralgia
-------- Fatigue worsens with exertion, plus post-exertional malaise
-------- Neuropsychological (cognitive) complaints
-------- Sleep disturbance

Fulfills:
-------- Major Criteria -------- Four or More Minor Symptom Criteria
----
Assessment:
-------- Fits CFS criteria -------- Does Not Meet CFS criteria
-------- Fits Idiopathic Chronic Fatigue (ICF) Criteria --Significant fatigue not meeting full CFS criteria
-------- Does not fit CFS or ICF criteria
-------- Has atypical features of CFS/ICF or is unclear

APPENDIX B

PEDIATRIC ME/CFS CASE DEFINITION WORKSHEET[110]

Subject: **Date:** **Examiner:**

To meet criteria for pediatric ME/CFS the subject must have had 3 months of medically unexplained fatigue; post-exertional malaise; unrefreshing sleep or sleep disturbance; widespread or migratory myofascial, joint, abdominal or head pain; two or more neuro-cognitive manifestations (such as impaired memory, difficulty focusing or slowness of thought); and at least one symptom from two of three categories: autonomic, neuroendocrine, or immune. Symptoms must be moderate or severe to meet criteria

I. **Symptoms**: ME/CFS symptoms must have persisted or recurred during the past **three months** of illness

II. **Post-exertional malaise**: With even non-strenuous activity there must be a loss of physical or mental stamina, rapid/sudden muscle or cognitive fatigability, post-exertional malaise and/or fatigue and a tendency for other associated symptoms within the patient's cluster of symptoms to worsen. The recovery is slow, often taking 24 hours or longer

III. **Sleep**: Unrefreshing sleep or disturbance of sleep quantity or rhythm disturbance

IV. **Pain**. At least one symptom from any of the following:

- ☐ Myofascial and/or joint pain
- ☐ Abdominal and/or head pain

V. Two or more **neurocognitive manifestations**:

Impaired memory	Slowness of thought
Difficulty focusing	Need to focus on one thing at a time
Difficulty finding the right word	Trouble expressing thought
Frequently forget what wanted to say	Difficulty comprehending information
Absent mindedness	Frequently lose train of thought
Difficulty recalling information	New trouble with math or other educational subjects

VI. At least one symptom from two of the following three categories:

☐ **Autonomic manifestations**: Neurally mediated hypotension, postural orthostatic tachycardia, delayed postural hypotension, palpitations with or without cardiac arrhythmias, dizziness, disturbed balance, shortness of breath

☐ **Neuroendocrine manifestations** Recurrent feelings of feverishness and cold extremities, subnormal body temperature and marked diurnal fluctuations, sweating episodes, intolerance of extremes of heat and cold, marked weight change-loss of appetite or abnormal appetite, worsening of symptoms with stress

☐ **Immune manifestations**: Recurrent flu-like symptoms, non-exudative pharyngitis, repeated fevers and sweats, lymph nodes tender to palpation, new sensitivities to food, odors, or chemicals

Exclusionary conditions:

- ☐ **Active disease processes** that could explain chronic fatigue
- ☐ **Active psychiatric conditions** that may explain the presence of chronic fatigue, such as:
  1. Childhood schizophrenia or psychotic disorders
  2. Bipolar disorder
  3. Active alcohol or substance abuse
  4. Active anorexia nervosa or bulimia nervosa
  5. Severe depressive disorders

Subjects may have concomitant disorders that do not adequately explain fatigue such as school phobia, separation anxiety, anxiety disorders, somatoform disorders, milder depressive disorders, multiple chemical sensitivities, and fibromyalgia

DIAGNOSIS:

- ☐ Severe ME/CFS (meets criteria for categories I, II, III, IV, V and VI)
- ☐ Moderate ME/CFS (meets 5 of the 6 categories; also only one symptom is needed for VI)
- ☐ Atypical ME/CFS (meets four or fewer criteria categories)

© Charles W. Lapp, MD, 2009. May be copied for individual use.

APPENDIX C

FUNCTIONAL CAPACITY SCALE	
The Functional Capacity Scale incorporates energy rating, symptom severity, and activity level. The description after each scale number can be used to rate functional capacity	
0 =	No energy, severe symptoms including very poor concentration; bed ridden all day; cannot do self-care (e.g. need bed bath to be given)
1 =	Severe symptoms at rest, including very poor concentration; in bed most of the day; need assistance with self-care activities (bathing)
2 =	Severe symptoms at rest, including poor concentration; frequent rests or naps; need some assistance with limited self-care activities (can wash face at the sink) and need rest afterwards for severe post exertional fatigue
3 =	Moderate symptoms at rest, including poor concentration; need frequent rests or naps; can do independent self-care (can wash standing at the sink for a few minutes) but have severe post exertion fatigue and need rest
4 =	Moderate symptoms at rest, including some difficulty concentrating; need frequent rests throughout the day; can do independent self-care (can take a shower) and limited activities of daily living (e.g. light housework, laundry); can walk for a few minutes per day
5 =	Mild symptoms at rest with fairly good concentration for short periods (15 minutes); need a.m. and p.m. rest; can do independent self-care and moderate activities of daily living, but have slight post exertion fatigue; can walk 10-20 minutes per day
6 =	Mild or no symptoms at rest with fairly good concentration for up to 45 minutes; cannot multitask; need afternoon rest; can do most activities of daily living except vacuuming; can walk 20-30 minutes per day; can do volunteer work – maximum total time 4 hours per week, with flexible hours
7 =	Mild or no symptoms at rest with good concentration for up to ½ day; can do more intense activities of daily living (e.g. grocery shopping, vacuuming), but may get post exertion fatigue if ‘overdo’; can walk 30 minutes per day; can work limited hours, less than 25 hours per week; no or minimal social life
8 =	Mild intermittent symptoms with good concentration; can do full self-care, work 40 hours per week, enjoy a social life, do moderate vigorous exercise three times per week
9 =	No symptoms; very good concentration; full work and social life; can do vigorous exercise three to five times a week
10 =	No symptoms; excellent concentration; over achiever (sometimes may require more sleep than average person)

Dr. Alison Bested © Dr. Lynn Marshall. May be copied for individual use.

APPENDIX D

ACTIVITY LOG

Name: ___________________________________ **Date Commencing:** _______________________

DAY	Monday	Tuesday	Wednesday	Thursday	Friday	Saturday	Sunday
**SLEEP**: Write number of hours slept and quality 1 = very poor 2 = poor 3 = fair 4 = good 5 = very good							
**Functional Capacity Scale**: Record your energy rating every hour using the scale 1 - 10.<br>**Activities** (please specify)							
6 a.m.							
7 a.m.							
8 a.m.							
9 a.m.							
10 a.m.							
11 a.m.							
12 p.m.							
1 p.m.							
2 p.m.							
3 p.m.							
4 p.m.							
5 p.m.							
6 p.m.							
7 p.m.							
8 p.m.							
9 p.m.							
10 p.m.							
11 p.m.							
# of minutes walked							
# of usable hours / day							

NUMBER OF USABLE HOURS / DAY = Number of hours NOT asleep or resting/meditating with eyes closed.

Dr. Alison Bested © Dr. Rosemary Underhill. May be copied for individual use.

Activity log:

- Keep it in a handy place
- Complete it every day
- Take your completed logs to your doctor/other health care provider at follow-up visits
- Your logs assist your doctor/other health care provider to adjust your treatment plan as needed
- Completed logs may reassure your insurance company of your active ongoing participation in your treatment

Completing activity log:

- You may change the times on the left hand side of the log to suit your usual schedule (e.g. if you usually get up at 10:00 a.m. and go to bed at 2:00 a.m., write 10:00 a.m. in as the first time, and adjust the other times accordingly)
- Please note your activities with one or two word(s) in the appropriate time slots (e.g. dressed, made bed, nap).
- Rest is defined as lying down, eyes shut, meditating or sleeping
- To better identify activity patterns coloring the log based on activity levels e.g. red for exercise, yellow for sedentary activity, blue for sleep, will help patients identify which activity pattern works best for them

APPENDIX E

RECOMMENDATIONS FOR PATIENTS PRIOR TO SURGERY

Anticipating Surgery? Recommendations for Persons with ME/CFS

Dr. Charles W. Lapp

CFS is a disorder characterized by severe debilitating fatigue, recurrent flu-like symptoms, muscle pain, and neurocognitive dysfunction such as difficulties with memory, concentration, comprehension, recall, calculation and expression. A sleep disorder is not uncommon. All of these symptoms are aggravated by even minimal physical exertion or emotional stress, and relapses may occur spontaneously.

Although mild immunological abnormalities (T-cell activation, low natural killer cell function, dysglobulinemias, and autoantibodies) are common in CFS, subjects are not immunocompromised and are no more susceptible to opportunistic infections than the general population. The disorder is not thought to be infectious, but it is ***not recommended that the blood or harvested tissues of patients be used in others.***

Intracellular magnesium and potassium depletion has been reported in CFS. For this reason, serum magnesium and potassium levels should be checked pre-operatively and these minerals replenished if borderline or low. Intracellular magnesium or potassium depletion could potentially lead to cardiac arrhythmias under anesthesia.

Up to 97% of persons with CFS demonstrate vasovagal syncope (neurally mediated hypotension) on tilt table testing, and a majority of these can be shown to have low plasma volumes, low RBC mass, and venous pooling.

Syncope may be precipitated by cathecholamines (epinephrine), sympathomimetics (isoproterenol), and vasodilators (nitric oxide, nitroglycerin, a-blockers, and hypotensive agents). Care should be taken to hydrate patients prior to surgery and to avoid drugs that stimulate neurogenic syncope or lower blood pressure.

Allergic reactions are seen more commonly in persons with CFS than the general population.

For this reason, histamine-releasing anesthetic agents (such as pentothal) and muscle relaxants (curare, Tracrium, and Mevacurium) are best avoided if possible. Propofol, midazolam, and fentanyl are generally well-tolerated. Most CFS patients are also extremely sensitive to sedative medications—including benzodiazepines, antihistamines, and psychotropics—which should be used sparingly and in small doses until the patient's response can be assessed.

Herbs and complementary and alternative therapies are frequently used by persons with CFS and FM. Patients should inform the anesthesiologist of any and all such therapies, and they are advised to withhold such treatments for at least a week prior to surgery, if possible.

Of most concern are:

- Garlic, gingko, and ginseng (which increase bleeding by inhibiting platelet aggregation)
- Ephedra or ma huang (may cause hemodynamic instability, hypertension, tachycardia, or arrhythmia)
- Kava and valerian (increase sedation)
- St. John's Wort (multiple pharmacological interactions due to induction of Cytochrome P450 enzymes), and Echinacea (allergic reactions and possible immunosuppression with long term use)

The American Society of Anesthesiologists recommends that all herbal medications be discontinued 2-3 weeks before an elective procedure. Stopping kava may trigger withdrawal, so this herbal (also known as awa, kawa, and intoxicating pepper) should be tapered over 2-3 days.

Finally, HPGA Axis Suppression is almost universally present in persons with CFS, but rarely suppresses cortisol production enough to be problematic. Seriously ill patients might be screened, however, with a 24 hour urine free cortisol level (spot or random specimens are usually normal) or Cortrosyn stimulation test, and provided cortisol supplementation if warranted. Those patients who are being supplemented with cortisol should have their doses doubled or tripled before and after surgery.

Summary Recommendations

- Insure that serum magnesium and potassium levels are adequate
- Hydrate the patient prior to surgery
- Use catecholamines, sympathomimetics, vasodilators, and hypotensive agents with caution
- Avoid histamine-releasing anesthetic and muscle-relaxing agents if possible
- Use sedating drugs sparingly
- Ask about herbs and supplements, and advise patients to taper off such therapies at least one week before surgery
- Consider cortisol supplementation in patients who are chronically on steroid medications or who are seriously ill
- Relapses are not uncommon following major operative procedures, and healing is said to be slow but there is no data to support this contention

I hope that you have found these comments useful, and that they will serve to reduce the risk of surgical procedures.

Charles W. Lapp, MD
Director, Hunter-Hopkins Center
Assistant Consulting Professor at Duke University Medical Center
Diplomate, American Board of Internal Medicine
Fellow, American Board of Pediatrics
American Board of Independent Medical Examiners

BIBLIOGRAPHY

1. Bates DW, Buchwald D, et al., "Clinical laboratory findings in patients with CFS," 1995 Jan 9, Arch Int Med 155:97-103
2. Klimas NG, Salvato FR, et al., "Immunologic abnormalities in CFS," 1990 Jun, J Clin Microbiol 28(6): 1403-1410
3. Caligiuri M, Murray C, Buchwald D, et al., "Phenotypic and functional deficiency of natural killer cells in patients with CFS," 1987 Nov 15, J Immunol.;139(10):3306-13
4. Cox IM, Campbell MJ, Dowson D, "Red blood cell magnesium and CFS," 1991 Mar 30, Lancet 337: 757-760.
5. Burnet RB, Yeap BB, Chatterton BE, Gaffney RD, "Chronic fatigue syndrome: is total body potassium important?" Med J Aust. 1996 Mar 18;164(6):384.
6. Bou-Houlaigah I et alia, "The relationship between neurally mediated hypotension and the chronic fatigue syndrome," JAMA 1995; 274:961-967
7. Streeten D & Bell DS, "Circulating blood volume in CFS," J of CFS 1998; 4(1):3-11
8. Kowal K, Schacterele RS, Schur PH, Komaroff AL, DuBuske LM, "Prevalence of allergen-specific IgE among patients with chronic fatigue syndrome," Allergy Asthma Proc. 2002 Jan-Feb;23(1):35-39
9. Ang-Lee MK, Moss J, Yuan CS, "Herbal medications and perioperative care," 2001 Jul 11, JAMA 286(2):208-216
10. Demitrack MA, Dale JK, Straus SE et alia,"Evidence for impaired activation of the hypothalamic-pituitary-adrenal axis in patients with chronic fatigue syndrome," J Clin Endocrinol Metab. 1991 Dec;73(6):1224-34

Appendix F ME/CFS - Chronic Fatigue Syndrome/ Myalgic Encephalomyelitis

Fact Sheet

What is ME/CFS?

Chronic fatigue syndrome also known as myalgic encephalomyelitis or ME/CFS is a serious, complex and disabling illness, which causes incapacitating fatigue coupled with pain, cognitive problems, sleep dysfunction and other immune, neurological and autonomic symptoms. The patient's ability to function is substantially reduced. The key feature of the syndrome, post-exertional malaise, is a worsening of symptoms following minimal physical or mental activity which can persist for hours, days or even weeks and is not relieved by rest. The pathological loss of energy of ME/CFS is very different from the fatigue felt by healthy people following strenuous exercise or who are living under stress.

Chronic fatigue syndrome (CFS) is an inappropriate name for this serious illness. Fatigue is universal in healthy people who have undergone too much activity or who have had insufficient rest. Chronic fatigue is also a frequent symptom in numerous other illnesses. The name ignores other abnormalities found in the illness and trivializes the severity of the symptoms. CFS is a global illness and in many parts of the world it is known as myalgic encephalomyelitis (ME). The acronym ME/CFS is currently gaining in popularity worldwide. The illness is sometimes also known as chronic fatigue immune dysfunction syndrome (CFIDS).

Who gets ME/CFS?

At least one million Americans have ME/CFS, but less than 20% have been diagnosed. ME/CFS affects all races, all socio-economic groups and all ages. The most common onset is between 20 and 50 years of age. Four times as many women have ME/CFS as men, but the gender ratio is almost equal in affected children.

What causes ME/CFS?

The cause of ME/CFS is uncertain. Several factors may be involved. ME/CFS usually occurs as sporadic (isolated) cases, but clusters of cases have occurred worldwide. Some outbreaks have affected large numbers of individuals in a particular community, hospital, or school. In sporadic cases, 20% of patients have another family member with the illness. These facts suggest that both genetic and environmental factors may contribute to the illness.

ME/CFS frequently starts with acute, "flu-like" symptoms and immune system changes found in ME/CFS are similar to immune system changes found in some viral infections. A number of infectious agents have been found more frequently in patients with ME/CFS than in the general population, but no infectious agent has been proven to be the cause. To avoid any possible contamination of the blood supply, patients with ME/CFS should refrain from donating blood. Occasionally, ME/CFS has been triggered by environmental toxins, the receipt of an immunizing injection, or surviving a major trauma. Although depression and anxiety may occur secondary to the illness, research studies have shown that ME/CFS and major depressive disorder can be distinguished by behavioral, immunological and hormonal testing. The Centers for Disease Control and Prevention (CDC) recognizes CFS as an organic syndrome, not a psychiatric disorder.

Main symptoms and diagnosis

Diagnosing ME/CFS is difficult. There is currently no specific diagnostic test for the illness. The diagnosis is made clinically from the pattern of symptoms and the exclusion of other fatiguing illnesses. The diagnosis depends upon the patient's symptoms meeting the criteria of one of several "case definitions." The 1994 Fukuda et al. case definition[1] is the most commonly used. However, this case definition was intended as a research tool and excludes some patients who genuinely have ME/CFS but do not precisely satisfy the case definition's criteria. The newer Canadian clinical case definition[2] was produced to overcome this problem. The Canadian clinical case definition includes more symptoms commonly found in patients with ME/CFS.

Both case definitions require:

1. the new onset of unexplained, persistent, or relapsing physical and mental fatigue that has lasted for at least six months, and has substantially reduced activity levels. The fatigue is not the result of ongoing exertion and is not relieved by rest
2. that the patient be clinically evaluated by taking a medical history, performing a clinical examination, and arranging appropriate medical testing to ex-

clude other fatiguing illnesses

The Fukuda et al. case definition:
also requires the presence of four of the following eight symptoms:
Post-exertional malaise lasting more than 24 hours; non-refreshing sleep; muscle pain; painful joints without redness or swelling; a new type or dramatically increased severity of headache; difficulty in concentration or short-term memory; sore throats; tender lymph glands in the neck or armpits.

The Canadian clinical case definition:
also includes:
1. Malaise and worsening of other symptoms occurring after exertion. Recovery is delayed by more than 24 hours
2. Unrefreshing sleep, nighttime insomnia, and/or daytime hypersomnia (excessive sleep)
3. Widespread, migratory or localized pain in muscles or joints (without swelling), or headache of a new type or increased severity
4. At least two neuro-cognitive symptoms, including: confusion; impaired concentration and short-term memory; difficulty finding words and/or numbers; disorientation; hypersensitivity to light, noise, or emotional overload; disturbed balance
5. At least one symptom from two of the following three subcategories:
a. Autonomic manifestations such as: orthostatic intolerance (OI), neurally mediated hypotension (NMH), postural orthostatic tachycardia (POTS), dizziness, facial pallor, palpitations, irritable bowel syndrome, urinary frequency, shortness of breath
b. Neuroendocrine manifestations including low body temperature, intolerance to heat and cold, feeling feverish, sweating, abnormal appetite, or symptoms that worsen with stress
c. Immune manifestations including tender lymph nodes, recurrent sore throats, recurrent flu-like symptoms, or new sensitivity to food, medications or chemicals

The pattern of symptoms differs in different patients and symptoms may vary in severity from day to day or during the day.

Progress and Recovery
ME/CFS often starts suddenly with a 'flu-like' illness, but sometimes the onset is gradual, over months or years. Patients may be very ill at illness onset, but the diagnosis is often delayed because by definition, the diagnosis of ME/CFS cannot be established for six months. A provisional diagnosis can often be made earlier. This may lessen the impact of the illness by ensuring that the patient has adequate rest.

Patients with ME/CFS tend to improve slowly and then reach a plateau. At this time, the severity of their illness varies between the extremes of some patients who are homebound, and others who are able to go out to work. ME/CFS patients who work may require some reasonable accommodations and they often need extra rest. Remissions and relapses are common. Relapse is frequently caused by over-exertion or an infectious illness. A few patients slowly get worse. Recovery rates are uncertain and vary from 5% to 30% in the first five years. After five years, recovery is less likely. Recovered patients often find that they need extra rest.

Management and Treatment
Establishing the diagnosis of ME/CFS will usually give the patient much relief. There is no medication, which will cure the illness. Management includes treatment for the relief of individual symptoms; encouraging patients to adapt their lifestyle to live within their existing capabilities (pacing of activities); giving advice on nutrition. Joining a support group for patients with ME/CFS can be helpful.

In some patients, there is an overlap of symptoms between ME/CFS and the following syndromes: fibromyalgia (FMS); multiple chemical sensitivities (MCS); and gulf war syndrome (GWS). It can also be difficult to distinguish ME/CFS from Lyme disease, rheumatoid arthritis, lupus, multiple sclerosis, psychiatric disorders and other illnesses.

References
Fukuda K, et al. The Chronic Fatigue Syndrome: A comprehensive approach to its definition and study. *Annals Int. med.* 1994; 121:953-959. 2. Carruthers BM, et al. ME/CFS Clinical working case definition, diagnostic and treatment protocols. *J. C F S* 2003; 11(1):7-115.

Modified from the NJCFSA fact sheet on ME/CFS with permission.

ME/CFS: A Primer for Clinical Practitioners

Index

A
academic examinations for children, 29, 30
accommodations for patients, 7,30,44
acknowledging ME/CFS is real, 18
ACTH injection, 10,15
activities of daily living, 11,20,21,22,38,45
activity and exercise, 10,17,20,21,22,24,28,38,40,43
activity log, 21,36,39,40
activity management, 7,28
activity plans, 21
acupuncture, 19,26
acute infection, 3,6,7,8,9,15,24,41,43
ADHD as a mis-diagnosis, 29
adolescents, 7,12,29,30,
adrenal function, 9,26
adults, 7,8,12,26,29,30
aerobic metabolism, 10,11,20
age, 6,26,29,43
age of onset, 6,43
alanine transaminase (ALT), 14
alcohol use, 15,25,37
aldosterone, 10,15
allergies, 13,15,16,24,25,41
ALT (alanine transaminase), 14
Ampligen, 25,28
anaerobic metabolism, 10,11,20
analgesics, 19
anemia, 13,15
anger, 16,23,27
anovulatory cycles 29
antinuclear antibodies, 9,14,15
anxiety disorders, 9,15,16,17,19,23,37,43
apoptosis, 11
arginine vasopressin, 10
arthritis, 13,15,19,44
assessment of function, 7,11,27,38-40
ATP/ADP cycling, 11,20 attention deficit hyperactivity disorder (ADHD), 29
attention span, 10
autoimmune disease, 13,15,16
autonomic nervous system disturbances, 3,6,8,9,10,12,14, 15,16,17,23,29,37,43,4

B
B complex, 25
B. burgdorferi, 24
bacterial infections, 3,8,24
baths, therapeutic, 19
bedridden, 7,17,22,45
behavioral disorders as misdiagnoses in children, 29
biofeedback, 26
biological abnormalities, 3
biomarkers, 11
biotin, 25
bladder dysfunction, 9,16,24
bloating, gastrointestinal, 24
blood count, 14
blood donation, 30
brain abnormalities 8,10,14
brain imaging, 3,10
brain perfusion, 10
breast-feeding - see lactation, 9
buspirone, 10

C
caffeine reduction or elimination, 18,22,23,35
calcium, 14
Canadian clinical case definition, 7,11,29,43,44
Candidiasis, 25
Cardio-pulmonary exercise testing, 11
Cardio-vascular dysfunction and ME/CFS, 8,10,15,16,24
Care-givers, role, 27,28
case definitions, 6,7,8,11,17,29,36,43,44
causal factors, 8
celiac disease, 13,14,15,16
cell-mediated immunity, 9,26
Centers for Disease Control and Prevention (CDC), 3,43
cerebral activity, 10
cerebral circulation, 29
cerebrospinal fluid, 10,25
CFIDS (chronic immune dysfunction syndrome), 6,43
chamomile tea, 26
chest pain, 14
chest X-ray, 14,15
child rearing, 29
childbirth, 28,29
children, 7,12,26,29,30,43
chiropractic, 19,26
chronic B Cell activation, 3
chronic Epstein Barr virus infection, 6,8
chronic fatigue immune dysfunction syndrome (CFIDS), 3,6,36,43
chronic immune activation, 3,9
chronic T Cell activation, 3,9
chronic infection, 3,6,8,9,24,31,43
chronicity of illness, 7
CK (creatinine kinase), 14
clinical care, 7,17
clinical diagnosis, 7,12,14,15,18,24,26,27,28,29,37,43,44
clinical worksheet, 12,13,36,37
cluster outbreaks, 6,43
co-existing medical conditions, 7,16
cognition, 29
cognitive behavioral therapy, 23
cognitive testing, 3,10
cognitive problems, 6,18,20,22,23,43
co-morbidity, 11,13,17,27
competitive sports, 30
complementary medicine, 26
concentration, 9,10,12,17,38,41,44
confusion, 12,17,44
core symptom, 8,11

correspondence schooling, 30
cortisol variation, 9,14,16,41
course of illness, 7
C-reactive protein, 14
creatinine kinase (CK), 14
crimson crescents, 14
cure,20,23,26,44
current living circumstances, 11
cytokines, 9
cytotoxic T cells, 3

D
darkness, importance for sleep, 18
depression, 3,9,13,17,23,28,43
depressive disorders, 37
detruser instability, 24
developmental abnormalities in offspring, 29
DHEA response, 10
disability insurance, 7
diagnosis, 3,7,11,12,14,15,17,18,24,26,27 28,29,37,43,44
diastolic dysfunction, 10
diet, 7,24,25,26
dietary supplements, 22,25,28,42
differential blood count, 14
differential diagnosis, 14,15
disability determination, 7,11
distractability, 10
diurnal fluctuations, 9,17
dizygotic twins, 8
dizziness, 24,37,44
dysmenorrhea, 29
dyspareunia, 29
dysuria, 24

E
early diagnosis, importance of, 7
echocardiography, 10
educational materials, 7,18,23
EEG, 3,10
eicosapentanoic acid, 25
EKG/ECG, 14,15
electrolytes, 14,24
electrical massagers, 19
emergence of other illness, 7
emotional distress, 8,9,12,16,18,41
employment, 7
endocrine disorders, 3,9,12,13,14,15,16,37,44
endometriosis, 16,24,2
endoscopy, 14,15
energy envelope, 20,21,28
energy metabolism, 3,20
energy production abnormalities, 8,10
enteroviruses, 8
environmental toxins 8,9,43
epidemiology, 6
Epstein-Barr virus, 6,8
Epworth Sleepiness Scale, 22
erythrocyte sedimentation rate (ESR), 14
essential fatty acids, 25
estrogen, 29
etiology, 8
exacerbation of illness, 6,14,20,29
exclusion of other fatiguing illnesses, 7,11,13,15,16,36,37,43
exercise and activity, 10,11,17,20,21,22,43
exercise provocation, 8
exercise recommendations, 21,22
exertional challenge, 8

F
factitious disorder by proxy as a mis-diagnosis in children, 29
familial ME/CFS, 8
family counseling for families with children, 23,30
family role, 18,23,27
fatigue and post-exertional malaise, 6,9,11,12,17,18,20,21,22, 36,37,38,41,43
fatigue relief, 6,21,2
feeling faint, 10,24
fertility, 28,29
fibroids, 29
fibromyalgia, 13,14,15,16,19,37,44
financial resources, 7
fludrocortisone, 24
fluid metabolism, 3,10,24,25
flu-like illness, 7,8,9,12,17,20,26,37,41,43,44
folic acid, 12,25
follow-up, 26,27,40
food avoidance, 16,18,24,25
food rotation, 25
fructose intolerance, 24
frustration, 16,27
full blood count, 14
full spectrum light, 18
Functional capacity scale - Appendix C, 38
Fukuda criteria, 6,11,36,43,44

G
gammaretrovirus, 8
gastrointestinal symptoms, 14,15,16,24,25
gender distribution, 8,43
gene expression, 8,11,43
gene sequencing, 3
generalized anxiety disorder (GAD), 15,17
genetic inheritance, 8
Giardia lamblia, 15
ginger tea, 26
glucose metabolism in brain, 10
gluten intolerance, 16,24,25
glyconutrients, 26
goal of treatment, 18
gonad function, 29
Gradual onset, 7
gray matter, 10
growth hormone response, 10
guilt, 17
gynecological problems, 18,29

H
health improvement, 7,18,21,22,23,25,26
health-care practitioner's role, 7

heart palpitations, 9,10,12,24,37,44
herbal preparations and pregnancy, 28
herbal remedies, 26
herpes virus, 8,24
HHV (human herpes virus), 8
higher functioning patients, 7,22
HLA antigens, 3
Holter monitoring, 10
home schooling for children, 30
homeopathy, 26
hormone replacement therapy (HRT), 29
hot flashes 29
house-bound, 7,17,27,28,45
HPA axis function, 9,10,16
human herpes virus (HHV), 8
hydrotherapy, 19
hydroxycobalamin, 25
hypersomnia, 12,18,44
hypocortisolism, 9
hypotension, 10,12,14,16, 23,24,37,41,42,44
hypothalamic pituitary axis function, 9,10,16
hysterectomy, 29

I

IACFS/ME International Association for Chronic Fatigue Syndrome/ME, 1,2,3,6
illness management, 7,17,18,24,25,26,27,28,30,44
illness severity, 8,10,11,12,22,26,27,37,43,44
illness worsening factors, 7,12,17,21,22,26,27,28,30,37,43,44
immune activation, 3,9,41
immune dysfunction, 6,26,43
immune function, 8,15,41,42
immune modulators, 25
immune symptoms, 9,17
immune system abnormalities in ME/CFS, 8,9,14,41,42
immune system boosters, 26
immunizations, 7,30
immunoglobulin, 14,15
Imunovir, 25
indigestion, 24
individualized education plan (IEP) for children, 30
Individuals with disabilities education act (IDEA), 30
infections, 8,9,15,24,41,43
infectious diseases, 13,15,30
infectious triggers, 3,9
inheritance, 8
insomnia , 12,19,20,29,44
interferon, 9
interleukins, 9
interstitial cystitis, 16,24,29
intestinal diseases, 13,24
iron studies, 14
irritable bladder syndrome, 16
irritable bowel syndrome, 9,12,13,16,24,44
isoprinosine, 25

L

labor, childbearing, 28,29
laboratory tests, 14,17
lactation, 26
lactic acid, 3,11
lactose intolerance, 16,24,25
laziness as a mis-diagnosis in children, 29
learning difficulties in offspring, 29
learning new information, 10
libido, 29
light-headedness, 12,24
live vaccines, 30
liver function tests, 14
long-term perpetuation, 8
low blood volume, 10,24,30
low IGF1, 10
low somatomedin, 10
low-functioning patients, 27,28
Lyme disease, 3,9,1315,44
lymph nodes, 12,14,17,36,37,44

M

magnesium, 25,41,42
major depression, 3,17
malignancies, 13,15
management in children, 30
massage, 19,26
MCS - see multiple chemical sensitivity, 15,16,24,30,37,44
ME/CFS, 2-4, 6-21, 23-30,36,37,41,43,44
medical documentation, 7,19,30
medical history, 11,13,44
medications during pregnancy, 28
medications for cognitive problems, 23
medications for depression, 22,23
medications for fatigue 7, 22
Medications for sleep, 19,22,26
meditation, 19
menopause, 16,29
mental fogginess, 18
mental overexertion, 7
migraine headache, 16,19
miscarriage, 28
mis-diagnoses in children, 29
mitochondrial dysfunction, 8,10
mitochondrial myopathy, 11
mitral valve prolapse, 16
monitoring, 7,10,22
monozygotic twins, 8
morning stiffness, 18
mortality, 26
mRNA, 11
multidisciplinary team approach, 7
multiple chemical sensitivity, 15,16,24,30,37,44
multiple sclerosis, 3,13,15,44
multisystem dysregulation, 9
muscle liniments, 19
myalgic encephalomyelitis, 1,3,6,43
myalgic encephalopathy, 6
mycoplasma, 24

N

naps, 18,38,40
narcotics, 19,20

National Institutes of Health (NIH), 3
natural killer cells, 9,26,41,42
nausea, 12,16,24,27
neurally-mediated hypotension (NMH), 10,12,14,16,37,42,44
neurocognitive dysfunction, 8,12,13,37,41
neuroendocrine dysfunction, 9,14,37,44
neurological abnormalities, 3,6,13,15,16,43
neuropeptide Y, 10
new infections, 7,8,9,43
niacin, 25
noise, 10,12,17,18,44
nomenclature, 6
non-exclusionary conditions, 11,13,15,16
non-pharmacological interventions for pain, 19
non-pharmacological interventions for sleep, 18,26
non-specific T-wave changes, 10

O

Oligo-ovulatory cycles, 29
olive leaf, 26
opiates, 19,20
opportunistic infections, 8,42
oral contraceptives, 29
organophosphates, 8,15
orthostatic intolerance, OI, 9,12,14,16-19,24,27,29,30,45
osteoporosis, 14,25,29
ovarian cysts, 29
overlapping medical conditions, 13,16
oxidative stress, 11
oxidative system, source of energy, 10,11,20

P

pacing - of activity, 18,19,21,22,44
pain, 6,9,12-14,16,18,19 20,24,26, 30,36,37,41,43,44
pain, treatment of in ME/CFS, 19,20,26,28-30
pallor, 12,14,44
palpitations in ME/CFS, 9,10,12,24,37,44
panic disorder, 17
parasitic infection, 8,24
pathophysiology, 3,8-11,20
patient education, 18,23,30,37
patient impairments, 3,7,10,11,12,20,21,27,30,37, 44
patient self-management 18,22
pediatric case definition Appendix B, 37
pediatric ME/CFS, 30,36,37
pelvic pain patients, 16,29
PEM (post-exertional malaise), 6,8,911,12,14,17,20,36,37,43,44
pepermint tea, 26
pesticides, 8
pharmacological interventions, 7,18,19,20,22,23,25,26,29,30, 41
pharyngitis, 14,37
phosphate, 8,14,15,20
physical examination, 13,14,17,37,44
physical therapy, 19,20,21
physical overexertion, 7,26
phytoestrogens, 29
picnogenol, 26
pine bark, 26
post-exertional malaise, 6,8,911,12,14,17,20,36,37,43, 44
post-partum depression, 28
post-partum recovery, 28
postural orthostatic tachycardia syndrome (POTS), 10,12,14,16,24,37,44
post-viral fatigue syndrome (PVFS), 6
potential for improvement, 7,18,20,21,-26,28,29
precipitating factors, 8,9,24,25,43
predisposing factors, 8,9,24,25,43
pregnancy, 29
pre-illness functioning, 11
premenstrual syndrome, 16,29
pre-morbid health, 7,26
pressure stockings, 24
prevalence, 6,7,27,29
prevalence in children and adolescents, 29
primary psychiatric disorder, 13
processing speed of brain, 10
progesterone treatment, 29
prognosis, 26, 30
prolactin response, 10
prolapsed mitral valve, 16
provocation studies, 8
psychiatric disorders - relationship to ME/CFS, 13,15-17,37,43,44
pulmonary disease, 13,15
punctate white spots, 10
pyridostigmine, 10

Q

Q fever, 8,15
quality of life, 7,20

R

race, 6,43
Raynaud's phenomenon, 16
recommendations for patients prior to surgery - Appendix E, 41
recumbent exercise, 24
reflux, 16,24
relapse,7,13,14,24,26,30,41,42, 44
remissions, 7,13,26,44
renal function, 14,20
rest, 6,7,10,12,18,21,22,26, 28,29, 30,38,39,40,43,44
rheumatoid factor, 9,15
Rintatolimod, 25,28
Rituximab, 3,25
RNase L, 2-5A synthetase, 9
Romberg test, 14
routine laboratory testing, 11,13,14,17

rTEMS (transcranial magnetic stimulation), 19

S
salt, 24
schooling, 30
school phobia as a mis-diagnosis in children, 29,37
sedating medications, 18,19
selenium, 25
serotonin, 19
severely ill ME/CFS patients and exercise, 28
severity of illness, 8,10-12,22,26,27,37,43,44
Sicca syndrome, 16

sleep disturbance in ME/CFS, 16,18,36

sleep dysfunction, 13,17,18, 22,37,43,44,
sleep latency, 10,15
sleep pattern disruption, 18,37, 39
sleep, treatment, 18,19,22
sleepiness, 22
slow gastric emptying, 15,24
social history, 11
socio-economic, 6
spinal fluid studies, 3,10
sporadic ME/CFS, 6,8,43
standard of care, 17
stress, 15,27,28,30,37,41,43,44
stretches, therapeutic, 19,22,28
suborbital dark shadows, 14
suicide evaluation, 17
surgery, 30,41,42
surgical delivery of newborn, 28-29
susceptibility to ME/CFS, 8
symptom flare-up, 11,17,21,30
symptom fluctuation, 17,22,37
symptom reduction, 18
symptom treatment, 17-26
symptom worsening, 7,12,17, 21,22,26-28,30,37,43,44
symptoms, 3,6,7,9-29,36-38,41,43,44

T
tai chi, 19,22
tandem stance test, 14
team approach, 7
temporo-mandibular joint syn-drome, 16
tender point examination, 14
TH2 response, 9
thyroid antibodies, 9
thyroid function, 9,14
thyroiditis, 16
tilt table testing, 10,15,41
tissue donation, 30
T-lymphocyte activation, 9
transcranial magnetic stimula-tion (rTMS), 19
transcutaneous electrical nerve stimulation (TENS) 19
treating fatigue, 20,27
treating post-exertional ma-laise, 20
treatment for ambulatory pa-tients, 17-27
treatment of bedridden pa-tients, 7,27-28
treatment of housebound pa-tients, 7,27-28
treatment, 17-30,41-42,44
treatment in children, 30
treatment of wheelchair bound patients, 7,27-28
tutoring for children, 30
T-wave flattening, 10
T-wave inversions, 10
twin studies, 3,8

U
urethral syndrome, 24
urinalysis, 14
urinary problems, 12,24,44

V
ventricular lactate, 10,11
viral antibodies, 8
viral infection, 3,6,8,9,24,43
visual imagery, 10
Vitamin B12, 25
vitamin B6, 25
vitamin C, 25
Vitamin D, 25-hydroxy-cholecalciferol 14,25,27
vomiting in pregnancy, 28
vulvodynia 16,29

W
weight change, 12,17,37
wheelchair dependent, 27
widespread pain, 12,14,19,37,44
wind down activities for sleep, 18
word retrieval, 10,12
working memory, 10
worksheet, 7,11,12-13,36,37
World health organization, 6
worsening, 7,12, 17,21,22,27,28,30,37,43,44

X
XMRV, 8

Y
yam therapy, 29
Yoga therapy, 19,22

Z
zinc, 25,26

About the Artist

Renée Rabache started her journey as a professional visual effects artist, mural painter, world traveler, polyglot, yoga teacher, counselor, and dive master. Having contracted ME/CFS, she is now mostly housebound and bedridden. Even the simplest of daily tasks break her energy bank account. Options for accessing her skills and talents, and for sharing them, have been much diminished.

Consequently, it is with great pride and joy that she offers her artwork for the cover of the *Physicians' Primer*. The image shows the patients - who hold the answers - imprisoned in their bodies, homes, and beds.

Ms. Rabache writes: *"In trying to unlock the puzzle of this complex, multi-system illness, it is essential that the medical community listen to what the patients are saying. We can show science and medicine where to start looking, where to keep looking and where the cracks in the foundation are. Every day we live in a most intimate relationship with this illness, a cruel tormentor that has isolated us, erased our former lives, and left us just a shadow of our former selves. Moment by moment in unpredictable, punishing waves, patients live through something private and terrible, which is as yet incomprehensible to those who are not at the mercy of this invisible neuro-immune maelstrom.*

When doctors and patients begin to collaborate and emerge from their respective cages of conditioned thought, conventional doctrine, and academic abstraction, we will grab hold of the key, which we collectively forge, and use it for the benefit of all: - physicians, patients, and the whole global community.

… Let us come together and open the door to hope, health, and a vibrant life."
