# Supplementary material for: The Qigong of Prolong Life With Nine Turn Method Relieve Fatigue, Sleep, Anxiety and Depression in Patients With Chronic Fatigue Syndrome: A Randomized Controlled Clinical Study
Source: Front Med (Lausanne). 2022 Jun 30;9:828414. doi: 10.3389/fmed.2022.828414 (PMC9280429; doi:10.3389/fmed.2022.828414)
Supplement: Supplementary file 2 [file Data_Sheet_2.docx]

|  | CBT（n=45） | |  | PLWNT（N=45） | |  | *PLWNT VS. CBT* |
| --- | --- | --- | --- | --- | --- | --- | --- |
|  | T_0_ (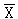S) | T_1_ (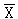S) | *Within group p* | T_0_  (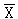S) | T_1_  (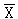S) | *Within group P* | *Between group P* |
| **Primary outcome: MFI-20** | | | | | | |  |
| general fatigue | 10.931±  2.840 | 7.340±  3.363 | 0.000* | 10.560±2.896 | 6.511±  2.897 | 0.000* | 0.701 |
| physical fatigue | 10.620±  3.632 | 6.727±  3.136 | 0.000* | 9.067±  3.962 | 4.511±  2.785 | 0.000* | 0.055 |
| reduced activity | 9.870±  3.584 | 6.545±  3.201 | 0.000* | 8.622±  3.6200 | 4..911±2.502 | 0.000* | 0.891 |
| reduced motivation | 8.092±  3.630 | 5.114±  2.479 | 0.000* | 8.200±  4.257 | 4.644±  2.854 | 0.000* | 0.171 |
| mental  fatigue | 9.881±  3.318 | 6.318±  2.720 | 0.000* | 10.022±4.104 | 5.578±  2.759 | 0.000* | 0.085 |
| **Secondary outcome: PSQI** | | | | | | |  |
| Total score | 7.708±  2.920 | 5.295±  2.351 | 0.000* | 6.756±  3.523 | 4.200±  2.085 | 0.000* | 0.088 |
| subject sleep quality | 1.560±  0.693 | 1.159±  0.079 | 0.001* | 1.444±  0.724 | 0.956±  0.424 | 0.000* | 0.995 |
| sleep latency | 1.712±  0.895 | 1.386±  0.803 | 0.017* | 1.333±  1.066 | 0.933±  0.720 | 0.011* | 0.727 |
| sleep duration | 1.021±  0.783 | 0.409±  0.612 | 0.000* | 0.911±  0.821 | 0.200±  0.405 | 0.000* | 0.645 |
| habitual sleep efficiency | 0.442±  0.725 | 0.364±  0.710 | 0.483 | 0.644±  1.171 | 0.378±  0.912 | 0.209 | 0.103 |
| sleep disturbance | 1.621±  0.614 | 1.204±  0.456 | 0.000* | 1.467±  0.625 | 1.022±  0.452 | 0.000* | 0.771 |
| sleep medicine using | 0.111±  0.487 | 0.091±  0.287 | 0.803 | 0.200±  0.757 | 0.089±  0.468 | 0.168 | 0.702 |
| daytime dysfunction | 1.273±  0.618 | 0.681±  0.594 | 0.000* | 1.022±  0.657 | 0.356±  0.609 | 0.000* | 0.543 |
| **Secondary outcome: HADS** | | | | | | |  |
| anxiety | 7.685±  3.924 | 5.727±  3.047 | 0.000* | 7.000±  3.855 | 4.111±  2.113 | 0.000* | 0.507 |
| depression | 6.706±  3.882 | 4.704±  3.034 | 0.001* | 6.844±  4.033 | 3.444±  2.563 | 0.000* | 0.056 |

Supplementary file 2 shows the changes primary and secondary outcomes in fatigue, sleep, anxiety and depression scores measured by MFI-20, PSQI, and HADS scales with ITT analysis.
